# Visualizing endogenous Rho activity with an improved localization-based, genetically encoded biosensor

**DOI:** 10.1242/jcs.258823

**Published:** 2021-09-08

**Authors:** Eike K. Mahlandt, Janine J. G. Arts, Werner J. van der Meer, Franka H. van der Linden, Simon Tol, Jaap D. van Buul, Theodorus W. J. Gadella, Joachim Goedhart

**Affiliations:** 1 Swammerdam Institute for Life Sciences, Section of Molecular Cytology, van Leeuwenhoek Centre for Advanced Microscopy, University of Amsterdam, Science Park 904, 1098 XH Amsterdam, The Netherlands; 2 Molecular Cell Biology Lab at Dept. Molecular Hematology, Sanquin Research and Landsteiner Laboratory, Plesmanlaan 125, 1066CX Amsterdam, The Netherlands

**Keywords:** Biosensor, Rho GTPase, Endothelial cell, Thrombin, Rho

## Abstract

Rho GTPases are regulatory proteins, which orchestrate cell features such as morphology, polarity and movement. Therefore, probing Rho GTPase activity is key to understanding processes such as development and cell migration. Localization-based reporters for active Rho GTPases are attractive probes to study Rho GTPase-mediated processes in real time with subcellular resolution in living cells and tissue. Until now, relocation Rho biosensors (sensors that relocalize to the native location of active Rho GTPase) seem to have been only useful in certain organisms and have not been characterized well. In this paper, we systematically examined the contribution of the fluorescent protein and Rho-binding peptides on the performance of localization-based sensors. To test the performance, we compared relocation efficiency and specificity in cell-based assays. We identified several improved localization-based, genetically encoded fluorescent biosensors for detecting endogenous Rho activity. This enables a broader application of Rho relocation biosensors, which was demonstrated by using the improved biosensor to visualize Rho activity during several cellular processes, such as cell division, migration and G protein-coupled receptor signaling. Owing to the improved avidity of the new biosensors for Rho activity, cellular processes regulated by Rho can be better understood.

This article has an associated First Person interview with the first author of the paper.

## Abstract (precis)

**Summary:** The dT-2xrGBD location-based Rho biosensor relocalizes more efficiently than other sensors of this type, and this sensor enables the observation of endogenous Rho activity in cultured cells.

## INTRODUCTION

Rho GTPases function as a molecular switch; they are turned on when guanosine triphosphate (GTP) is bound and turned off when GTP is hydrolyzed to guanosine diphosphate (GDP) (Bos et al., 2009; Ridley, 2015). The conversion from inactive, GDP-bound Rho GTPase, to active, GTP-bound Rho GTPase, requires Rho guanine exchange factors (Rho GEFs) (Rossman et al., 2005). The bound GTP is hydrolyzed to GDP by the intrinsic but slow GTPase activity, thereby inactivating the Rho GTPase. This process is enhanced by GTPase-activating proteins (GAPs) (Bos et al., 2009). RhoA (Ras homology family member A) is one of 20 Rho GTPases in humans, and has two closely related homologs, RhoB and RhoC (Burridge and Wennerberg, 2004; Wheeler and Ridley, 2004). We will use ‘Rho’ throughout the article, which refers to all three isoforms. Active Rho mainly localizes at the plasma membrane, due to its prenylated C-terminus (Garcia-Mata et al., 2011). However, a fraction of Rho has been found at the Golgi. Inactive Rho, in comparison, can be extracted from the plasma membrane by Rho-specific guanine nucleotide dissociation inhibitors (RHOGDIs) (Garcia-Mata et al., 2011). Rho and other Rho GTPases orchestrate the cytoskeleton dynamics and thereby cell features, such as adhesion, cell migration, cell division, cell morphology and polarity (Lawson and Ridley, 2018). Therefore, they are involved in complex processes like transendothelial migration and wound healing (Heemskerk et al., 2014). Rho GTPase signaling occurs in a spatial and temporally defined manner. While biochemical assays are well established and sensitive, they only show the average of a population with no spatial resolution and a poor time resolution, in the order of minutes (Pertz, 2010). To address this issue, genetically encoded fluorescent biosensors have been engineered. These tools enable the visualization of protein activities in single living cells with micrometer spatial and sub-second temporal resolution (Greenwald et al., 2018; Mehta and Zhang, 2011; Miyawaki and Niino, 2015).

Several genetically encoded biosensors are available to visualize active (GTP-bound) Rho GTPase. These sensors can broadly be divided in two classes, namely, Förster resonance energy transfer (FRET)-based biosensors and localization-based biosensors (Pertz and Hahn, 2004). Each class has its own advantages and disadvantages (Pertz, 2010). Regardless of the type, the sensors use a G protein-binding domain (GBD), which has a higher affinity for the active GTP-bound state of the Rho GTPase relative to the inactive, GDP-bound Rho GTPase. Unimolecular Rho GTPase FRET-based biosensors consist of the Rho GTPase itself, a GBD and a FRET pair, which is commonly a cyan (CFP) and a yellow fluorescent protein (YFP). The sequence of the domains is an important aspect of the optimization of these sensors (Fritz et al., 2013). The dimerization optimized reporter for activation (DORA) Rho-based FRET sensor consists of Rho and the GBD of protein kinase N1 (PKN1) (Van Unen et al., 2015). Upon Rho GTPase activation, the binding domain binds the GTP-bound Rho GTPase. This conformation change leads to a FRET ratio change with a relatively small dynamic range. By design, these FRET sensors report on the balance between activating guanine exchange factors (GEFs) and inactivating GTPase-activating proteins, instead of visualizing endogenous Rho-GTP. In contrast, localization-based sensors solely consist of a fluorescent protein fused to a GBD, which has a high affinity for the active GTP-bound state. These sensors visualize endogenous Rho-GTP. For instance, when Rho GTPase activation occurs locally at the plasma membrane, the sensor will accumulate at that location. A potential drawback is that background signal of the unbound biosensor in the cytosol, which may occlude the bound pool and reduce the dynamic range. In addition, most of the Rho GTPase-binding domains are able to bind different Rho GTPases, so relocation sensors (sensors that relocalize to the native location of active Rho GTPase) tend to be less specific than FRET sensors (Pertz and Hahn, 2004). Unlike the relocation sensor, the FRET-based biosensor also contains the Rho GTPase and thereby its specificity is not only determined by the binding specificity of the GBD. For example, by changing the Rho GTPase homolog, a certain level of specificity can be achieved for RhoA, RhoB and RhoC (Reinhard et al., 2016). A key advantage of relocation probes is their simple design, utilizing only a GBD and a single fluorescent protein. Usage of a single fluorescent protein simplifies multiplexing of biosensors or the combination with optogenetics. However, the greatest advantage of the localization-based biosensor is the visualization of endogenous Rho activity at its unaltered location in the cell.

The Rho FRET sensors achieve subcellular resolution to a certain extent, but due to their design they may not localize in the manner as endogenous Rho (Michaelson et al., 2001).

Since temporal and spatially tightly regulated Rho GTPase activity is important for their functionality, we set out to test Rho relocation biosensors for their ability to visualize endogenous Rho activity with a high spatial resolution.

Thus far, two Rho relocation biosensors have been published. First, the relocation Rho sensor based on anillin (also known as ANLN), consisting of a GBD, C2 and pleckstrin homology (PH) domain, called anillin-homology domain (AHD)+PH-GFP or anillin Rho binding domain (AniRBD), which was first described in 2000 (Munjal et al., 2015; Piekny and Glotzer, 2008). Secondly, the rhotekin G protein binding domain (rGBD)-based eGFP-rGBD Rho sensor, which was reported in 2005 (Benink and Bement, 2005), and for which different versions appeared over the years, such as Venus-rGBD (O'Neill et al., 2018), mCherry 2xrGBD (Davenport et al., 2016), delCMV-EGFP-rGBD (Graessl et al., 2017) and 3xGFP-rGBD (Bement et al., 2015), but with no comparison was published.

In order to understand and to expand the potential of relocation-based Rho sensors, we first systematically compared and subsequently optimized several Rho relocation sensors in cell-based assays. We quantified their relocation efficiency, checked their specificity for Rho in comparison to Cdc42 and Rac1 and finally, we showcased their potential by visualizing endogenous Rho activity in human endothelial cells.

## RESULTS

### Optimizing the rGBD relocation Rho sensor

**Fig. 1.**
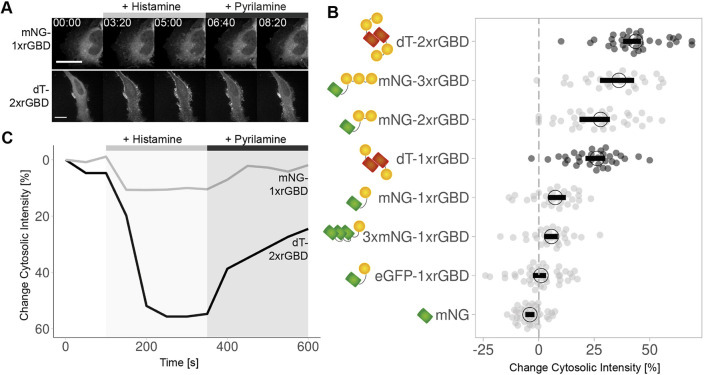
**The optimized dimericTomato-2xrGBD Rho sensor has the best location efficiency.** (A) Spinning disk still images of a HeLa cells expressing the CMVdel-mNeonGreen-1xrGBD Rho sensor (upper panel) or CMVdel-dimericTomato-2xrGBD Rho sensor (lower panel) and H1R (not shown), which were stimulated with 100 μM histamine after 150 s and 10 μM pyrilamine after 350 s. The images match with the perturbation that is indicated for the plot in C. Times are min:s from the start of the recording. Scale bars: 25 µm. (B) Change in cytosolic intensity for CMV-mNeonGreen, CMV-eGFP-1xrGBD, CMVdel-3xmNeonGreen-1xrGBD, CMVdel-mNeonGreen-1xrGBD, -2xrGBD and -3xrGBD, and CMVdel-dimericTomato-1xrGBD and -2xrGBD (schematics on left) in HeLa cells expressing H1R, upon stimulation with 100 μM histamine. Each dot represents an individual cell. The median of the data is shown as a black circle and the 95% confidence interval for each median, determined by bootstrapping, is indicated by the bar. The gray dashed line indicates no change in cytosolic intensity. The data is from two biological replicates based on two independent transfections. The number of cells per condition is: 3xmNG-1xrGBD=27, dT-1xrGBD=32, dT-2xrGBD=33, eGFP-1xrGBD=40, mNG=39, mNG-1xrGBD=28, mNG-2xrGBD=34, mNG-3xrGBD=26. (C) Time traces of the change in cytosolic intensity for the displayed cells for the mNeonGreen-1xrGBD sensor in gray and for the dimericTomato-2xrGBD sensor in black. mNG, mNeonGreen; dT, dimericTomato; rGBD, rhotekin G protein binding domain.

To optimize the relocation Rho biosensor, we tested it in a cell-based assay. The eGFP-rGBD biosensor consists of an enhanced green fluorescent protein (eGFP) and a rhotekin G protein-binding domain (rGBD). It reports active (i.e. GTP-bound), endogenous Rho at the plasma membrane of *Xenopus* oocyte during wound healing (Benink and Bement, 2005). We verified the performance in HeLa cells, overexpressing the histamine 1 receptor (H1R, also known as HRH1), as we have previously demonstrated, activation of H1R by histamine activates Rho in HeLa cells (Van Unen et al., 2016). In a resting HeLa cell, the rGBD sensor localizes in the cytosol. Upon histamine addition, it binds the activated, endogenous Rho and thereby relocalizes to the plasma membrane, where active Rho is localized, and it relocalizes to the cytosol when pyrilamine, a histamine antagonist, is added (Fig. 1A,C; Fig. S1A, Movies 1 and 2). However, the relocation of the single rGBD monomeric fluorescent protein sensor is hardly detectable. To optimize the rGBD sensors by increasing the avidity, we constructed single, double and triple rGBD mNeonGreen fusions. A triple mNeonGreen single rGBD version was created, to increase the brightness of a single sensor. A dimericTomato single and double rGBD sensor was generated to study the influence of a dimeric fluorescent protein on the sensor. We compared the change in cytosolic intensity of the sensor upon histamine addition for different versions of the rGBD sensor (Fig. 1B; Fig. S1B). We found that the change in cytosolic intensity increased with each added rGBD domain. The change from a single to a triple mNeonGreen did not change the performance of the sensor. Interestingly, a dimericTomato single rGBD sensor localizes as well as a monomeric fluorescent protein double rGBD sensor. Hence, a dimeric fluorescent protein provides another way to improve the performance of the location sensor by doubling the number of binding domains. However, the change of monomeric fluorescent protein, from eGFP to mNeonGreen, seems to slightly improve the relocalization as well. This effect may be explained by the higher expression level of the eGFP-1xrGBD construct under a regular CMV promoter, where the mNeonGreen-1xrGBD construct is expressed under the low expression CMVdel promoter. Nevertheless, no correlation between change in cytosolic intensity and absolute fluorescence intensity was measured (Fig. S1C,D). We conclude that the dimericTomato-2xrGBD sensor shows the best relocation efficiency, with a median change in cytosolic intensity of close to 45%.

### Coexpression comparison of Rho location sensors

**Fig. 2.**
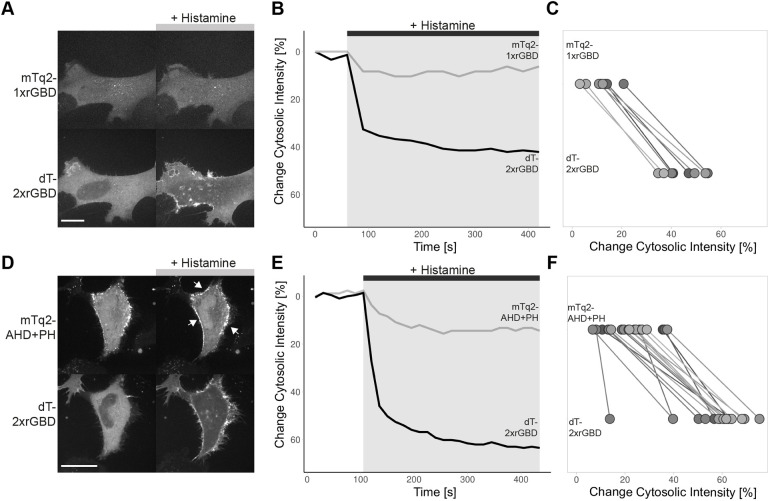
**The optimized dimericTomato-2xrGBD Rho sensor localizes more efficiently than the published single rGBD Rho sensor and anillin AHD+PH Rho sensor.** (A) Spinning disk still images of a HeLa cell expressing H1R (not shown) and CMVdel-mTurquoise2-1xrGBD (upper panel) and CMVdel-dimericTomato-2xrGBD (lower panel) before (left) and 5 min after (right) stimulation with 100 μM histamine. Scale bar: 25 µm. (B) Traces of the change in cytosolic intensity for CMVdel-dimericTomato-2xrGBD (black) and CMVdel-mTurquoise2-1xrGBD (gray) for the displayed cell. (C) Plot of the change in cytosolic intensity for cells expressing both CMVdel-mTurquoise2-1xrGBD (upper row) and CMVdel-dimericTomato-2xrGBD (lower row). Each line connects the values for one cell. Number of cells from a single replicate, *n*=8. (D) Spinning disk still images of a HeLa cell expressing H1R (not shown) and CMV-mTurquoise2-Anillin(AHD+PH) (upper panel) and CMVdel-dimericTomato-2xrGBD (lower panel) before (left) and 5 min after (right) stimulation with 100 μM histamine. Arrows indicate anillin(AHD+PH) sensor clusters. Scale bar: 25 µm. (E) Traces of the change in cytosolic intensity for CMVdel-dimericTomato-2xrGBD (black) and CMV-mTurquoise2-Anillin(AHD+PH) (gray) for the displayed cell. (F) Plot of the change in cytosolic intensity for cells expressing both CMV-mTurquoise2-Anillin(AHD+PH) (upper row) and CMVdel-dimericTomato-2xrGBD (lower row). Each line connects the values for one cell. Number of cells from a single replicate, *n*=18.

We then performed a co-expression experiment, to directly compare the relocalization of the dimericTomato-2xrGBD sensor to the mTurquoise2-1xrGBD sensor in the same HeLa cell (Fig. 2A; Movie 3), avoiding cell-to-cell heterogeneity. Where the single rGBD sensor only shows a 10% drop of cytosolic intensity compared to baseline, the optimized dimericTomato-2xrGBD sensor shows a 40% decrease in cytosolic intensity (Fig. 2B). Comparing multiple cells, it becomes evident that the dimericTomato-2xrGBD sensor consistently relocalizes more efficiently than the mTurquoise2-1xrGBD sensor (Fig. 2C). Additionally, the dimericTomato-2xrGBD sensor was compared to an alternative localization-based sensor for Rho (Fig. 2D; Movie 4), which utilizes the AHD and PH domain of anillin (Munjal et al., 2015; Piekny and Glotzer, 2008). The anillin sensor (AHD+PH) showed a 15% decrease in cytosolic intensity (Fig. 2E), but it also relocalizes to striking punctuate structures upon histamine stimulation. These structures did not seem to represent local high activity of Rho, as the optimized rGBD sensor in the same cell showed no such locally clustered Rho activation, but rather a homogenous activation at the membrane and a 60% drop in cytosolic intensity. Similar punctuate structures were observed in endothelial cells, when stimulated with the strong Rho activator thrombin (Movie 5). The comparison of multiple cells shows that the dimericTomato-2xrGBD sensor also relocalizes more efficiently than the mTurquoise1-AHD+PH sensor (Fig. 2F). Concluding, the optimized dimericTomato-2xrGBD sensor outperforms two existing Rho relocation sensors in a direct comparison.

### Specificity of rGBD for Rho

**Fig. 3.**
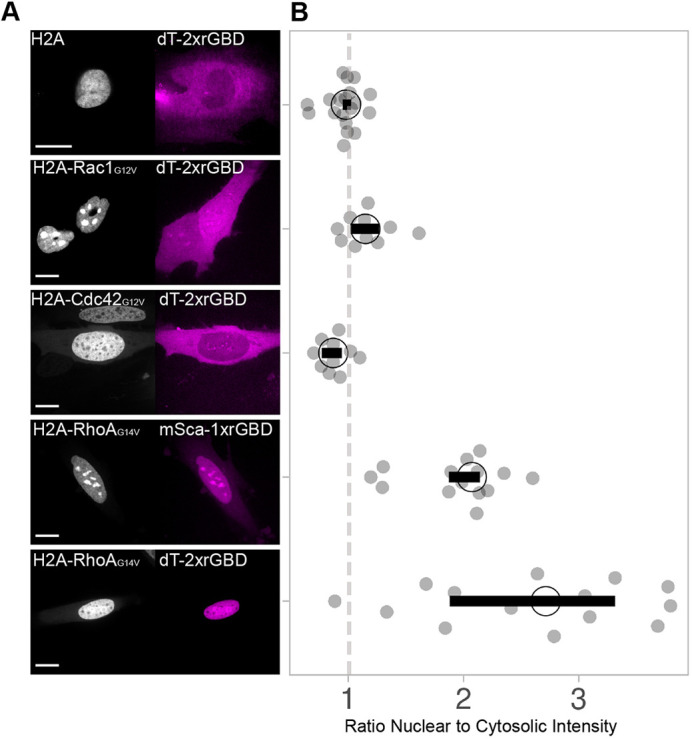
**The dimericTomato-2xrGBD Rho sensor is specific for Rho.** (A) Spinning disk images showing colocalization of dimericTomato-2xrGBD sensor or mScralet-I-1xrGBD sensor (magenta) with H2A-mTurquoise2-Rho GTPase-ΔCaaX (gray) in HeLa cells, for control H2A-mTurquoise2, and the indicated constitutively active forms (G12V,G14V) of the Rho GTPases Rac1, Cdc42 and RhoA. Scale bar: 20 µm. (B) Rho GTPase specificity, represented by the ratio of dimericTomato-2xrGBD or mScarlet-I-1xrGBD intensity in the nucleus to cytosol in H2A-mTurquoise2-Rho GTPase-ΔCaaX expressing HeLa cells. Each dot represents an individual cell. The dashed line indicates a ratio of one. The median of the data is shown as a black circle and the 95% confidence interval for each median, determined by bootstrapping, is indicated by the bar. The number of cells from a single replicate per condition is: H2A-Cdc42-dT2xrGBD=10, H2A-dT2xrGBD=19, H2A-Rac1-dT2xrGBD=10, H2A-RhoA-dT2xrGBD=14, H2A-RhoA-mSca-1xrGBD=14.

Next, we wanted to examine the selectivity of the sensor for Rho in comparison to other Rho GTPases in living cells. To this end, we generated nuclear localized, constitutively active Rho GTPases (H2A-mTurquoise2-RhoAG14V-ΔCaaX, H2A-mTurquoise2-Rac1G12V-ΔCaaX and H2A-mTurquoise2-Cdc42G12V-ΔCaaX), a strategy that was used before (Bery et al., 2019). The H2A histone tag, in combination with the removal of the CaaX box, allows the construct to completely localize in the nucleus, otherwise it is partly directed to the plasma membrane. With this approach, binding affinity can be assessed by colocalization of the location-based sensor with the applicable Rho GTPase. We co-expressed these constitutively active Rho GTPases in HeLa cells with CMVdel-dimericTomato-2xrGBD or CMVdel-mScarlet-I-1xrGBD and measured the intensity of the sensor in the nucleus in comparison to the cytosol (Fig. 3A,B). The rGBD sensors solely colocalized in the nucleus with RhoA but not with Rac1 and Cdc42, indicating that rGBD specifically binds constitutively active Rho. This is in line with previous studies in cell extracts and bacterial lysate (Reid et al., 1996; Ren et al., 1999). Comparing the single rGBD sensor (mScarlet-I-1xrGBD) with the dimericTomato-2xrGBD sensor, a higher nuclear to cytosolic intensity ratio for the multi-domain sensor was detected, supporting its higher affinity for Rho.

### Attempt to utilize GBDs of anillin and PKN1 to create a Rho location sensor

Given the successful improvement of the rGBD-based biosensor by increasing the number of binding domains, we explored whether the same strategy can be applied to the GBDs from PKN1 and anillin. The GBD of PKN1 (pGBD), which is used in the DORA Rho FRET sensor (Van Unen et al., 2015), was used as starting material for a relocation sensor. Moreover, a published relocation sensor AHD+PH based on anillin also contains, next to a GBD, a C2 and a PH domain (Munjal et al., 2015; Piekny and Glotzer, 2008). This sensor localizes in punctuate structures, which do not represent Rho activity (Fig. 2C; Movies 4 and 5). Here, we used only the GBD of anillin (aGBD) as a basis for another sensor.

**Fig. 4.**
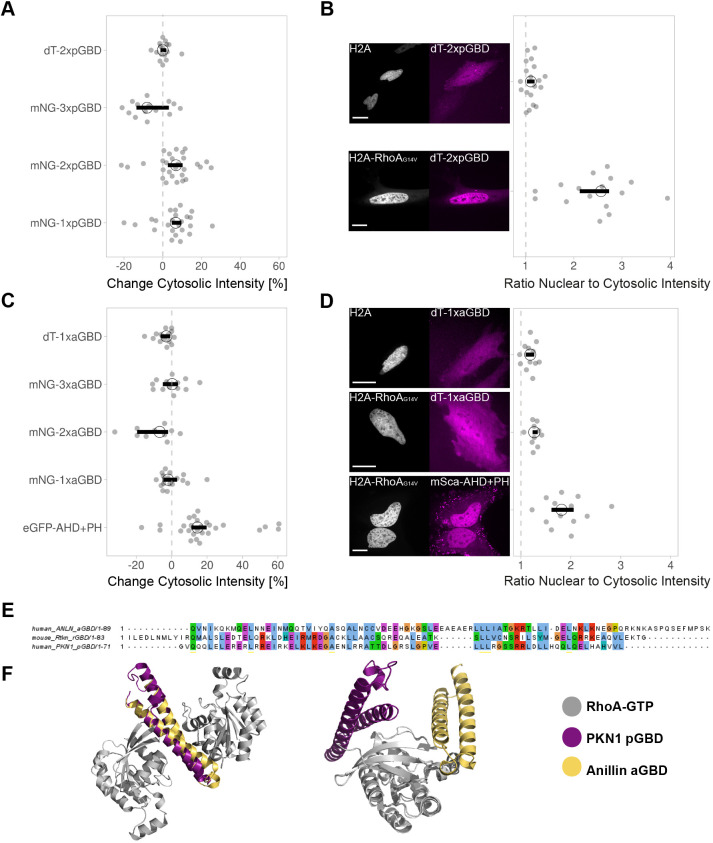
**The GBDs of anillin and PKN1 are not suitable for a relocation Rho sensor.** (A) Change in cytosolic intensity for CMVdel-mNeonGreen-1xpGBD, -2xpGBD and -3xpGBD, and CMVdel-dimericTomato-2xpGBD co expressed with H1R in HeLa cells upon stimulation with 100 μM histamine. The dashed line represents no change in cytosolic intensity. Each dot represents an individual cell. The median of the data is shown as a black circle and the 95% confidence interval for each median, determined by bootstrapping, is indicated by the bar. The number of samples per condition is: dT-2xpGBD=16, mNG-1xpGBD=24, mNG-2xpGBD=28, mNG-3xpGBD=14. (B) Spinning disk images showing colocalization of H2A-mTurquoise2-RhoAG14V-ΔCaaX or control H2A-mTurquoise2 with dimericTomato-2xpGBD in HeLa cells. Scale bars: 20 µm. RhoA binding, represented by the ratio of sensor intensity in the nucleus to cytosol in H2A-mTurquoise2-RhoAG14V-ΔCaaX expressing HeLa cells. The dashed line indicates a ratio of one. Each dot represents an individual cell. The median of the data is shown as a black circle and the 95% confidence interval for each median, determined by bootstrapping, is indicated by the bar. The number of cells from a single replicate per condition is: H2A-dT-2xpGBD=19, H2A-RhoA-dT2xpGBD=16. (C) Change in cytosolic intensity for CMVdel-mNeonGreen-1xaGBD, -2xaGBD and -3xaGBD, CMVdel-dimericTomato-1xaGBD and eGFP-anillin(AHD+PH) coexpressed with H1R in HeLa cells upon stimulation with 100 μM histamine. The dashed line represents no change in cytosolic intensity. Each dot represents an individual cell. The median of the data is shown as a black circle and the 95% confidence interval for each median, determined by bootstrapping, is indicated by the bar. The number of cells per condition is: dT-1xaGBD=14, eGFP-AHD+PH=27, mNG-1xaGBD=17, mNG-2xaGBD=10, mNG-3xaGBD=13. (D) Spinning disk images showing colocalization of H2A-mTurquoise2-RhoAG14V-ΔCaaX or control H2A-mTurquoise2 with dimericTomato-1xaGBD and mScarlet-I-anillin(AHD+PH) in HeLa cells. Scale bars: 20 µm. RhoA binding, represented by the ratio of sensor intensity in the nucleus to cytosol in H2A-mTurquoise2-RhoAG14V-ΔCaaX-expressing HeLa cells. The dashed line indicates a ratio of one. The median of the data is shown as a black circle and the 95% confidence interval for each median, determined by bootstrapping, is indicated by the bar. The number of cells from a single replicate per condition is: H2A-aGBD=12, H2A-RhoA-aGBD=10, H2A-RhoA-AHD+PH=13. (E) Amino acid sequence alignment for aGBD, rGBD and pGBD from MUSCLE and depicted with the clustalX color code, where green is polar, blue is hydrophobic, purple is negative charge, red is positive charge, yellow is prolines, orange is glycines, cyan is aromatic and white is unconserved. (F) Crystal structures of PKN1 G-protein binding domain (purple) and anillin G-protein binding domain (yellow), bound to RhoA GTP (gray). On the left, a structural alignment of PKN1 and anillin by their G-protein binding domains. On the right, a structural alignment of the bound RhoA molecules, showing the two binding positions at the RhoA molecule (PDB: anillin, 4XOI; PKN1, 1CXZ).

Using the same strategy as for the rGBD sensors, single, tandem and triple and dimericTomato versions of the sensor were created (CMVdel-mNeonGreen-1xpGBD, -2xpGBD and -3xpGBD, and CMVdel-dimericTomato-2xpGBD) and tested in H1R-expressing HeLa cells by stimulating endogenous Rho with histamine. None of the pGBD sensors showed a clear membrane localization upon stimulation with histamine (Fig. 4A). The increase in cytosolic intensity observed in some cells seems to be caused by changes in cell shape. Nevertheless, when CMVdel-dimericTomato-2xpGBD is co-expressed with H2A-mTurquoise2-RhoAG14V-ΔCaaX (constitutively active and nuclear located RhoA) in HeLa cells, pGBD accumulated in the nucleus (Fig. 4B), indicating that pGBD does bind constitutively active RhoA.

For the original anillin AHD+PH sensor, a variety of responses can be observed when H1R-expressing HeLa cells are stimulated with histamine. A small pool of cells showed a cytosolic intensity change of between 50% and 60%. A large pool showed a cytosolic intensity change of ∼15%. Plus, the sensor localized in a clustered, non-homogenous manner in all these cells. However, for the aGBD-based sensors (CMVdel-mNeonGreen-1xaGBD/−2xaGBD/−3xaGBD and CMVdel-dimericTomato-1xaGBD), no localization to the membrane upon histamine stimulation was observed (Fig. 4C). Additionally, when CMVdel-dimericTomato-1xaGBD and mScarlet-I-AHD+PH are coexpressed with H2A-mTurquoise2-RhoAG14V-ΔCaaX (constitutively active and nuclear located RhoA) in HeLa cells, only the AHD+PH (consisting of aGBD+C2+PH) construct localizes with the active RhoA (Fig. 4D). The aGBD by itself did not localize with the RhoA in the nucleus, indicating that it is not able to bind RhoA without the C2 and PH domain.

Given the different behavior of aGBD, pGBD and rGBD, we examined their amino acid sequence and structure. The amino acid alignment of the GBDs showed conserved hydrophobic residues (Fig. 4E). It also showed that all three domains contain the leucine repeats, which have been shown to interact with RhoA for pGBD (Maesaki et al., 1999). The superimposed crystal structures of aGBD and pGBD binding to RhoA-GTP showed a good overlap of the GBDs and revealed two different binding sites for RhoA (Fig. 4F). These sites have previously been described for pGBD (Maesaki et al., 1999) and this may be a general feature of GBDs. Unfortunately, no crystal structure is available for rGBD. The amino acid sequence and structure did not provide a clear explanation for the different behavior of the three GBDs.

In conclusion, the attempt to create a Rho location sensor from anillin and PKN1 was not successful.

### Visualizing endogenous Rho at the Golgi

**Fig. 5.**
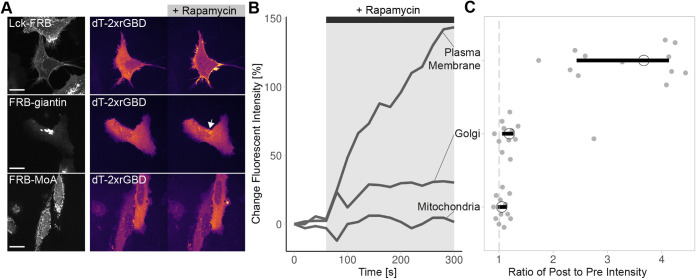
**The optimized dimericTomato-2xrGBD sensor shows organelle-specific Rho activity.** (A) Spinning disk still images of HeLa cells expressing FRB (part of the rapamycin hetero-dimerization system) anchored to the plasma membrane, Golgi and mitochondria (first column; Lck, giantin and MoA, respectively) and FKBP-p63-DH (counterpart of rapamycin hetero-dimerization system, not shown), showing localization of the dimericTomato-2xrGBD sensor pre activation (second column) and post activation with 100 nM rapamycin (third column). Arrow indicates intensity increase at Golgi. Scale bars: 20 µm. (B) Time traces of the dimericTomato-2xrGBD sensor intensity for the displayed cells at the indicated location. (C) Localization of the dimericTomato-2xrGBD sensor represented by the ratio of post to pre rapamycin activation intensity. The median is indicated by a black circle and the 95% confidence interval for each median, determined by bootstrapping, is indicated by the bar. The dashed gray line indicates a ratio of one. The number of cells from one replicate per condition is: Golgi=12, membrane=12, mitochondria=12.

In all of the previous experiments, endogenous Rho activity was detected at the plasma membrane. A subset of Rho is known to localize at the Golgi membrane (Zilberman et al., 2011). We challenged the sensitivity of the optimized Rho biosensor to detect activity of this smaller Rho fraction. To examine this, we used a rapamycin-induced heterodimerization system to recruit the Dbl homology (DH) domain, of the Rho activating GEF p63 (also known as ARHGEF25), to the membrane of the Golgi (Van Unen et al., 2015). We chose the plasma membrane as a positive control and mitochondria as negative control. Recruiting p63-DH to the plasma membrane caused a clear increase in Rho biosensor intensity at the plasma membrane (Fig. 5; Movie 6). Recruiting p63-DH to the Golgi is followed by a slight increase of Rho biosensor intensity at this organelle (Fig. 5; Movie 7). Recruiting p63-DH to mitochondria did not result in a clear increase of the Rho biosensor intensity (Fig. 5; Movie 8). In summary, the dimericTomato-2xrGBD sensor seems able to visualize activity of the small Rho fraction at the Golgi.

### Visualizing endogenous Rho activity in endothelial cells stimulated with thrombin

**Fig. 6.**
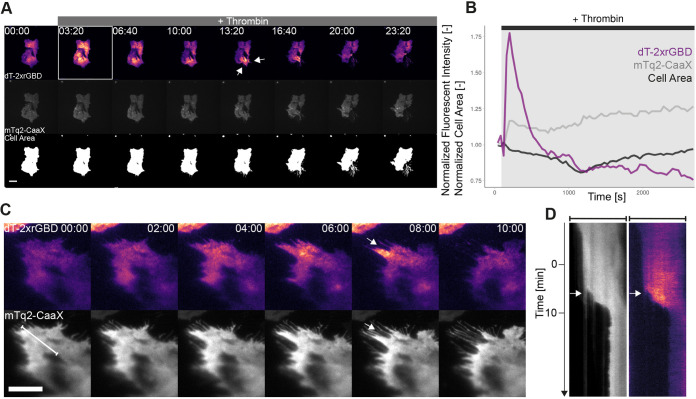
**Endogenous Rho activity in endothelial cells stimulated with thrombin.** (A) TIRF images of a cbBOEC stably expressing the dimericTomato-2xrGBD sensor (upper panel) and mTq2-CaaX (middle panel) stimulated with 1 U/ml human α-thrombin. The lower panel shows the cell area as a binary image. The boxed frame indicates the moment of global dimericTomato-2xrGBD intensity increase. Arrows indicate local dimericTomato-2xrGBD intensity increase followed by local contraction. Times are min:s from the start of the recording. Scale bar: 20 µm. (B) Time trace of the normalized fluorescence intensity for the dimericTomato-2xrGBD sensor (purple) and the mTq2-CaaX membrane label (gray) for the cell depicted in A. The normalized cell area (black) was measured for the white region shown in the lower panel of A. The same region was used as ROI for the fluorescence intensity measurements. (C) TIRF images of a cbBOEC stably expressing dimericTomato-2xrGBD sensor (upper panel) and mTq2-CaaX (lower panel). The cell was stimulated with 1 U/ml human α-thrombin 5 min prior to the imaging. Arrows indicate dimericTomato-2xrGBD intensity increase followed by contraction of the cell edge. Scale bar: 10 µm. (D) Kymograph of the cell depicted in C for along the white line. The fluorescence intensity of mTq2-CaaX is depicted in gray and the fluorescence intensity of dimericTomato-2xrGBD is depicted with the LUT mpl-magma. Arrows indicate dimericTomato-2xrGBD intensity increase followed by contraction. Indicated time correlates with the time of the still images in C (min:s after addition of thrombin).

To image endogenous Rho under physiologically relevant conditions, the dimericTomato-2xrGBD Rho sensor was expressed in primary cells, namely endothelial cells that are devoid of GPCR receptor overexpression, stimulated with thrombin. We turned to total internal reflection fluorescence (TIRF) microscopy to specifically image the dimericTomato-2xrGBD at the basolateral membrane of an endothelial cell. A membrane marker, mTurquoise2-CaaX, to correct for intensity changes unrelated to sensor relocation, was coexpressed. Endothelial cells were stimulated with human α-thrombin to activate endogenous receptors (Fig. 6A; Movie 9). A peak in Rho biosensor intensity with an ∼75% increase globally over the whole basolateral plasma membrane was measured within seconds after stimulation with human α-thrombin (Fig. 6B). The sensor intensity decreased to the base level after ∼6 min; this is in line with previous research (Heemskerk et al., 2016). The cell area decreased ∼20%, reaching the minimum at 20 min after stimulation, followed by recovering of the cell area to the original size over the course of 40 min after stimulation, which is in line with what is known about thrombin-responsive endothelial cells (Timmerman et al., 2015). The membrane marker showed a relatively small increase in intensity after stimulation and the curve did not show the same pattern as the Rho biosensor intensity curve (Fig. 6A,B). Therefore, we conclude that the increase in Rho biosensor intensity is caused by relocalization. The global increase in Rho biosensor intensity followed by cell contraction was robustly observed for multiple cells (Fig. S2A).

Since TIRF microscopy only visualizes Rho activity at the basolateral membrane, we imaged a cross section of the cell using lattice light sheet microscopy (Fig. S2B, Movies 10 and 11). These cross sections showed the increase in Rho biosensor intensity at the basolateral as well as at the apical plasma membrane. The (control) membrane marker showed no visible increase.

Following the first global increase in Rho biosensor intensity upon human α-thrombin stimulation, we also observed local increase followed by retraction in this cell area (Fig. 6C; Movie 12). Kymograph analysis showed that the increase of the Rho biosensor intensity is followed by a retraction of the periphery of the cell, and the increase in intensity is not reflected in the membrane marker (Fig. 6D). The local cell contraction confirmed that the Rho biosensor indeed sensed endogenous active Rho with high spatial resolution. Cell contraction is the expected cellular response upon Rho activation (Ridley and Hall, 1992).

It is important to note that even though the sensor binds endogenous Rho, it allows for sensitive cellular responses such as full cell division and cell contraction. Moreover, our data revealed that the Rho biosensor displays Rho activity at subcellular locations where Rho activity is expected.

### Visualizing endogenous Rho activity in several cellular processes

**Fig. 7.**
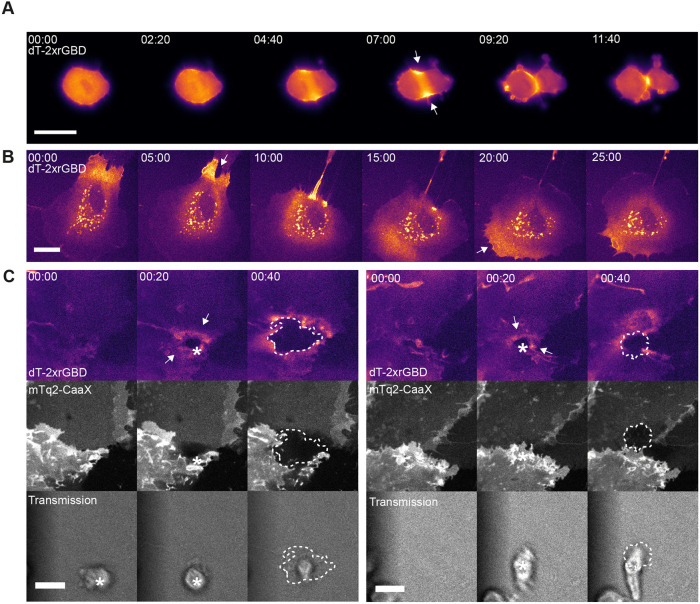
**Visualization of endogenous Rho activity in several cellular processes.** (A) Widefield images of a HeLa cell expressing dimericTomato-2xrGBD sensor going through cell division. Arrows indicate intensity increase at the location that will become the cleavage furrow. Scale bar: 25 µm. (B) Spinning disk images of a BOEC transiently expressing dimericTomato-2xrGBD sensor randomly migrating after cell division. Arrows indicate contracting cell edge with increased sensor intensity. Scale bar: 25 µm. (C) Spinning disk still images of a transendothelial migration event with cbBOECs grown in to a monolayer, expressing the dimericTomato-2xrGBD sensor (upper panel) and the membrane marker mTurquoise2-CaaX (middle panel). The transmigrating neutrophil is visible in the transmission panel; its position is indicated with an asterisk. The dashed line indicates where the neutrophil is underneath the cbBOECs. Arrows indicate increased dimericTomato-2xrGBD sensor intensity around the migration pore. Scale bars: 10 µm. Times are min:s after the start of transmigration.

Finally, we used the optimized biosensor to visualize endogenous Rho activation in several cellular processes that are known to be Rho mediated, with a focus on subcellular relocalization. Therefore, a HeLa cell expressing the dimericTomato-2xrGBD Rho biosensor going through cell division was imaged (Fig. 7A; Fig. S3A, Movie 13). The sensor localized clearly at the cleavage furrow where accumulation of active Rho has been reported (Piekny and Glotzer, 2008). Next, human endothelial cells expressing the dimericTomato-2xrGBD Rho biosensor were imaged during random migration, where localization of the sensor at the cell edge was followed by contraction of that cell edge (Fig. 7B; Fig. S3B, Movie 14). Finally, the process of neutrophil transendothelial migration was studied to see whether the dimericTomato-2xrGBD sensor could detect Rho activity at the migration pore, as it has been reported previously in experiments using the DORA Rho FRET sensor (Heemskerk et al., 2016). Indeed, an intensity increase in dimericTomato-2xrGBD sensor around the migration pore was observed, which was not detected in the mTurquoise-CaaX channel (Fig. 7C; Fig. S3C, Movie 15).

These results confirmed that the Rho biosensor relocalizes to cellular structures where Rho activity is expected.

## DISCUSSION

Rho relocation sensors have been used for more than a decade, but their use has been mostly limited to *Xenopus* oocytes, macrophages and *Drosophila* embryos (Benink and Bement, 2005; Jiang and Harris, 2019; Mason et al., 2016; O'Neill et al., 2018). Using the original eGFP-rGBD sensor in HeLa cells, we observed only a subtle relocalization. The reason for this poor performance in mammalian cell cultures has been unclear. We systematically increased the avidity by increasing the number of binding domains. This resulted in a drastic increase in the relocalization efficiency. Moreover, we demonstrated that the use of a dimerizing fluorescent protein is an efficient strategy to increase the avidity. The improved dimericTomato-2xrGBD sensor relocalizes with higher efficiency than the rGBD and AHD+PH sensors in a direct comparison. Consequently, our data show that this sensor is the preferred relocation sensor for the study of endogenous Rho activity in mammalian cells. We show that the dimericTomato-2xrGBD sensor can visualize endogenous Rho activity with high spatial resolution in expected locations, such as the cleavage furrow of a dividing HeLa cell and prior to and during cell retraction in endothelial cells.

Improving the rGBD probe by increasing the avidity was successful. This strategy, to utilize multiple repeating domains has also been effective for a PH domain-based lipid sensor and a cRAF-derived rat sarcoma (Ras)-binding domain Ras activity sensor (Augsten et al., 2006; Goulden et al., 2019). The avidity of the lipid sensor was increased with each added PH domain, indicating a cooperative effect, which decreased the off-rate of binding the plasma membrane. It is currently not clear how each of the GBDs of the dimericTomato-2xrGBD sensor contribute to Rho binding and the probe may bind between one and four Rho molecules. If the probe is capable of binding multiple Rho proteins, the binding efficiency will depend on the local density of Rho in the membrane.

Increasing the number of binding domains to generate relocation sensors from other Rho binding domains (i.e. aGBD and pGBD) was not successful. The previously reported AHD+PH Rho sensor was derived from anillin, a scaffold protein that connects Rho, actin and myosin, especially during cell division (Piekny and Glotzer, 2008). When we utilized only the aGBD domain and fused it to a fluorescent protein, we obtained a probe that did not colocalize with active RhoA, whereas AHD+PH does. This result is in line with the notion that only the synergistic action of aGBD, C2 and PH enables anillin to bind active Rho and to localize at the plasma membrane (Sun et al., 2015). However, we noticed that the AHD+PH sensor containing the aGBD, C2 and PH domain localizes in a punctate manner. These ‘dots’ were observed in both HeLa cells and endothelial cells, and were only observed with the AHD+PH Rho sensor. As aGBD does not localize in puncta, it seems that the localization is caused by domains other than of the Rho-binding domain, namely, the C2 and/or PH domain.

Strikingly, the pGBD that works in the DORA Rho FRET-based sensor does not work as a relocation sensor. The DORA FRET-based sensor for Rho uses the pGBD domain from PKN1, a member of the protein kinase C-related family of serine/threonine protein kinases, which mediate a Rho GTPase-dependent signaling pathway (Lim et al., 2006). Our data show that the pGBD location sensor binds to active Rho in a living cell assay. However, it does not relocalize upon Rho activation and the addition of multiple binding domains or a dimeric fluorescent protein does not improve its avidity.

Looking at the amino acid sequence of aGBD, pGBD and rGBD and their structures did not give an explanation for why rGBD works in a relocation sensor and the other two do not. The function of rhotekin is not clear, but it seems to lock Rho in the GTP bound state (Ito et al., 2018; Reid et al., 1996). We can only speculate that rhotekin has a stronger binding affinity for active Rho than anillin and PKN1 have. This idea is supported by a mass spectrometry-based study for active Rho binders, where rhotekin scores higher than anillin and PKN1 (Gillingham et al., 2019). This difference in affinity might explain its ability to function as a relocation sensor for Rho. The unimolecular FRET-based sensors that consist of Rho and a GBD probably require a lower binding affinity. This may explain why pGBD can be used in a FRET-based sensor but not as a relocation sensor. Unfortunately, we have not found a ‘recipe’ that would allow us to convert any Rho-binding protein into a relocation sensor.

Comparing relocation sensors to FRET sensors, both have their own advantages and disadvantages. Relocation sensor data lack the semi-quantitative aspect of ratio FRET measurements. Moreover, one has to consider that morphological changes can lead to fluorescence intensity changes (Dewitt et al., 2009). The ratiometric approach of the FRET sensor accounts for these morphological intensity changes, provided the intensity change is equal in both channels. A solution for intensity changes unrelated to the relocation of the biosensor is to co-image an inert plasma membrane-bound fluorescent marker. The optimized Rho biosensor intensity increase was mostly unrelated to membrane fluorescence intensity increase. The specificity of the relocation sensor is determined by the binding specificity of the GBD. The rGBD binds the three homologs RhoA, RhoB and RhoC but not to Rac1 and Cdc42 (Ren et al., 1999). To further improve the binding specificity, one either needs to screen for specific RhoA binders or design an artificial GBD. The latter has been done in the form of an anti RhoA-GTP nanobody (Keller et al., 2019). However, this nanobody did not show a great relocalization potential (Fig. S4).

Furthermore, use of the relocation sensor requires confocal microscopy or TIRF microcopy to spatially separate the bound from unbound probe, whereas FRET measurements are usually performed with widefield microscopes. However, the former mentioned techniques usually offer the higher resolution. Here, we have presented previously unachieved visualization of Rho activity at subcellular resolution. We observed local activation of Rho at the Golgi, which was not possible with the DORA Rho FRET sensor (Van Unen et al., 2015), indicating a higher sensitivity of the relocation sensor.

However, the optimal expression level is crucial for the dynamic range of the relocation sensor. Low concentrations of the sensor will show higher levels of relocalization, as a larger fraction of the sensor molecules binds the limited, active, endogenous Rho molecules. Nevertheless, if the concentration of sensor is too low, the fluorescent signal cannot be detected. To optimize the expression level, the CMVdel promoter, leading to a lower expression level, was applied (Watanabe and Mitchison, 2002). Even though, this minimal promoter improved the performance of the relocation sensor, a variety of expression levels was observed. Cell sorting could be applied to select for cells with the optimal expression level. Moreover, the biosensor concentration of relocation probes affects their performance. Although the diffusion of a soluble probe will not readily lead to differences in local availability in most cell types, this may be an issue in highly polarized cell types.

It is worth noting that the operating principle of the two sensor types is different. Whereas the Rho FRET sensor is a read out of endogenous GEF and GAP activity, the Rho location sensor detects endogenous Rho activity directly. The optimized Rho relocation sensor visualized endogenous Rho activity at its true location in the cell with great spatial detail.

Visualizing the endogenous Rho activity may interfere with the biological role of Rho, as the sensor binds endogenous Rho and may compete with natural effectors of Rho. As an example, the rGBD has been used as Rho inhibitor in zebrafish (Yoo et al., 2010). To limit the perturbation, the sensor should be expressed at a low level to allow Rho signaling. We demonstrate that low expression of the biosensor, through the truncated CMV promotor, did not inhibit cell division and cell edge retraction. Plus, endothelial cells expressing the sensor still show the typical reaction of contracting followed by spreading when stimulated with thrombin. Low expression results in a low fluorescence signal for the sensor. The dimericTomato-2xrGBD sensor has the advantage that, when it forms a dimer, one sensor unit contains two dimerTomato molecules and four rGBDs. To enhance the brightness per sensor molecule, one could introduce a triple fluorescent protein in combination with multiple rGBD, rather than searching for a strongly dimeric fluorescent protein. Using a triple fluorescent protein gives the choice of any characterized fluorescent protein, which will be an advantage for multiplexing. If cells express Rho at a higher level or if their shape allows visualization of a thicker part of the membrane in the *Z*-plane, the relocation sensor will localize more clearly, which might explain the different performance in different cellular systems. Another way to circumvent the influence of the cell shape on the location efficiency is TIRF microscopy. This has been undertaken for the rGBD sensor by two groups in U-2 OS cells and in *Drosophila* Schneider (S2) cells (Graessl et al., 2017; Verma and Maresca, 2019). While we were able to image dimericTomato-2xrGBD relocalization with a spinning disk set up, TIRF microscopy provides a better signal-to-noise ratio and less bleaching. These properties can be of great advantage while working with low expression of the biosensor.

The dimericTomato-2xrGBD genetically encoded, single-color fluorescence biosensor gives the opportunity to measure endogenous Rho activity with high spatial resolution. Single-color relocation sensors are ideal candidates for multiplexing experiments. Plus, the growing field of optogenetics needs single-color biosensors to detect the effect of optogenetic perturbations. The conventional CFP-YFP FRET sensor is incompatible with most blue light-induced optogenetic tools. Another research group has shown that relocation sensors combine well with optogenetics; for example, in experiments where the the Rho GEF leukemia-associated Rho guanine nucleotide exchange factor (LARG; also known as ARHGEF12) with the improved light-induced dimer (iLID) system was recruited to the membrane, driving cell migration, with Rho activity measured with Venus rGBD (O'Neill et al., 2018).

To conclude, we succeeded in visualizing endogenous Rho with high spatial and temporal resolution in living mammalian cells with the improved, single-color rGBD Rho relocation biosensor. We expect that the new probe will be a versatile tool to measure Rho activity in living cells and tissue. Beyond this, we imagine that multiplexing with Rho GTPase relocation sensors will be key to improving the understanding of complex cellular processes.

## MATERIALS AND METHODS

### Plasmid construction

#### rGBD

GFP-rGBD was Addgene plasmid #26732, deposited by William Bement. The reduced expression GFP β-actin plasmid was Addgene plasmid #31502, deposited by Rick Horwitz and Tim Mitchison. In this plasmid the base pairs 91–544 of the enhancer region in the CMV promoter are deleted; herein, this promoter is called CMVdel. The rGBD was excised with BsrGI and XbaI and cloned into a demethylated and likewise digested mCherry-C1 vector (Van Unen et al., 2015). mCherry was replaced with mNeonGreen/3xmNeonGreen using the AgeI and BsrGI restriction sites. The insert mNeonGreen-rGBD and the backbone reduced expression GFP β-actin plasmid were digested with AgeI and MluI. The digested products were ligated to create CMVdel-mNeonGreen-1xrGBD. A tandem and a triple rGBD were created by PCR amplification of rGBD from CMVdel-mNeonGreen with primers shown in Table S1 and digestion with BsrGI and SalI. The backbone CMVdel-mNeonGreen1xrGBD, or CMVdel-mNeonGreen-2xrGBD respectively, were digested with BsrGI and AvaI. Backbone and insert were ligated to generate CMVdel-mNeonGreen-2xrGBD and -3xrGBD.

To create different color fluorescent protein rGBD fusions, CMVdel-mNeonGreen-1xrGBD was digested with AgeI and BsrGI. The inserts dTomato, mScarlet-I and mTurquoise2 were digested with AgeI and BsrGI and ligated to the backbone. CMVdel-mNeonGreen-2xrGBD was digested with AgeI and BsrGI. The inserts dTomato was digested with AgeI and BsrGI and ligated to the backbone.

pLV-dT-2xrGBD was created by digesting the pLV backbone and the insert dT-2xrGBD with the restrictions enzymes EcoRV and ApaI and ligation of the two fragments.

#### aGBD

The anillin AHD+PH domain containing pEGFP-RhoA biosensor plasmid was Addgene plasmid #68026 (deposited by Michael Glotzer). The EGFP was replaced with mTurquoise2 by making use of the AgeI and BsrGI restriction sites.

The anillin G protein Binding Domain (aGBD) was defined as the amino acid sequence from residue 712 to 786, plus a disordered linker from residue 786 to 801 (Sun et al., 2015). The aGBD was amplified by PCR with primers shown in Table S1 from the pEGFP RhoA Biosensor plasmid. The first aGBD (residues 712–786) PCR product was digested with BsrGI and BamHI and ligated into a likewise digested CMVdel-mNeonGreen backbone, creating CMVdel-mNeonGreen-1xaGBD. For the tandem and triple aGBD, the n+1 aGBD (residues 712–801) PCR product was digested with BsrGI and SalI. The backbones CMVdel-mNeonGreen-1xaGBD and -2xaGBD were digested with BsrGI and AvaI. By ligating backbone and insert CMVdel-mNeonGreen-2xaGBD and -3xaGBD were created.

#### pGBD

The GFP-PKN1(full) was a gift from Peter Parker (Protein Phosphorylation Laboratory, London Research Institute, King's College London, UK) (Lachmann et al., 2011). The PKN1 G protein binding domain (pGBD) was defined as residue 30 to 100 as in the DORA biosensor (Van Unen et al., 2015). Owing to its high GC content, the sequence of pGBD was codon optimized with COOL (Chin et al., 2014) and the following gBlock was ordered with Integrated DNA Technologies (IDT):

5′-GA**TGTACA**GC**CTCGAG**GGGGTACAGCAGCAGCTGGAGCTGGAAAGAGAAAGGTTAAGAAGAGAGATCAGAAAAGAATTAAAGCTGAAGGAAGGAGCTGAGAACCTGAGGAGAGCCACCACAGATTTGGGAAGAAGCCTGGGCCCTGTGGAGTTATTATTAAGAGGCAGCAGCAGAAGGCTGGACCTGCTGCACCAGCAGCTGCAGGAGCTTCATGCCCACGTGGTGCTTTAA**GGATCC**GC-3′. Bold nucleotides indicate restriction sites for BsrGI, AvaI and BamHI.

The pGBD was amplified from the pGBD gBlock with primers shown in Table S1. The first pGBD PCR product was digested with BsrGI and BamHI and cloned in the likewise digested CMVdel-mNeonGreen backbone to create CMVdel-mNeonGreen-1xpGBD. For a tandem and triple pGBD, the n+1 pGBD PCR product was digested with SalI and BsrGI. The backbones CMVdel-mNeonGreen-1xpGBD and -2xpGBD were digested with BsrGI and AvaI. By ligating backbone and insert CMVdel-mNeonGreen-2xpGBD and -3pGBD were created.

#### H2A-RhoGTPase-ΔCaaX

Cdc42-G12V, Rac1-G12V and RhoA-G14V, obtained from https://cdna.org/, were PCR amplified with primers shown in Table S1. To remove the CaaX motif, the primer contains a mutation, replacing an A with a T, shown in bold followed by a stop codon. The original amino acid sequence CLVL is thereby changed to a serine followed by a stop codon. The PCR products were digested with NotI and BsrGI and ligated to the likewise digested backbone H2A-mTurquoise2-N1. This results in H2A-mTurquoise2-Cdc42-G12V-ΔCaaX, H2A-mTurquoise2-Rac1-G12V-ΔCaaX and H2A-mTurquoise2-RhoA-G14V-ΔCaaX.

An overview of all plasmids created in this study can be found in Table S2.

#### Other plasmids

The plasmid encoding histamine 1 receptor (H_1_R) was obtained from https://cdna.org/. The plasmids Giantin-FRB-mTurquoise2, mTurquoise2-FKBP12-p63(DH), ECFP(W66A)-FRB-MoA (Addgene plasmid #67904) and Lck-FRB-mTurquoise were described before (Van Unen et al., 2015). pLV mTurquoise2 CaaX was generated using HiFi Gibson cloning (NEB) of mTurquoise2 and CaaX into a pLV vector digested with MluI and XhoI.

#### Plasmid availability

The following plasmids, generated in this study, are available on Addgene (http://www.addgene.org/): 129625, dTomato-2xrGBD (C1 vector); 176098, dimericTomato-2xrGBD (pLV vector); 129624, mNeongreen-2xrGBD; 176091, mNeonGreen-3xrGBD; 129633, mNeonGreen-aGBD (anillin); 129634, mNeonGreen-pGBD (PKN1 codon optimized); 176094, H2A-mTurquoise2-CDC42-G12V-ΔCaaX; 176095, H2A-mTurquoise2-RAC1-G12V-ΔCaaX; and 176097, H2A-mTurquoise2-RHOA-G14V-ΔCaaX.

### Stable cell lines

Lentiviral particles were produced in HEK293T cells (CRL-3216, American Tissue Culture Collection; Manassas, VA, USA) transfected with TransIT (Mirus) using third generation packing plasmids (pHDMG·G VSV ENV, pHDM·HgpM2 GAG/POL, pRC-CMV-Rev1b REV) and pLV-mTurquoise2-CaaX in combination with pLV-dimericTomato-2xrGBD. Supernatant was harvested 2 and 3 days after HEK293T cell transfection, filtered (0.45 μm) and concentrated using Lenti-X Concentrator (TakaraBio cat #631232). Human umbilical vein endothelial cell (HUVECs) and cord blood blood outgrowth endothelial cells (cbBOECs) were transduced. Cells that were double positive for mTurquoise2-CaaX and dimericTomato-2xrGBD were sorted using a BD FACSAria™ cell sorter.

### Cell culture and sample preparation

HeLa cells (CCL-2, American Tissue Culture Collection; Manassas, VA, USA) were cultured in Dulbecco's modified Eagle's medium plus GlutaMAX™ (Gibco) with 10% fetal calf serum (Gibco) (DMEM+FCS) at 37°C in 7% CO_2_. For transfection 25,000 to 50,000 cells were seeded on round 24 mm diameter coverslip (Menzel, Thermo Fisher Scientific) in a 6-well plate with 2 ml DMEM+FCS. The transfection mix contained 1 µl linear polyethylenimine (PEI, pH 7.3, Polysciences) with a concentration of 1 mg/ml per 100 ng DNA and 0.5 to 1 μg plasmid DNA per well and was mixed with 200 μl OptiMEM (Thermo Fisher Scientific) per well. After 15 min incubation at room temperature, the transfection mix was added to the cells at 24 h after seeding.

Blood outgrowth endothelial cells (BOECs) were cultivated from healthy adult donor blood as described previously (Martin-Ramirez et al., 2012) and cbBOECs were cultivated from healthy donor umbilical cord. Cells were cultured in Endothelial Cell Growth Medium-2 BulletKit (CC-3162, Lonza) with 100 U/ml penicillin (Thermo Fisher Scientific) and 100 μg/ml streptomycin (Thermo Fisher Scientific) and 20% FCS (EGM+) at 37°C in 5% CO_2_. Culture dishes and coverslips were coated with 0.1% gelatin (CAS 9000-70-8, Merck) in phosphate-buffered saline (PBS) 30 min prior to cell seeding. Transfection was performed with 2 μg endotoxin free plasmid DNA, using the Neon™ Electroporation Transfection System (MPK5000, Invitrogen) with the associated Neon™ Transfection System 100 μl Kit (MPK10096, Invitrogen) generating a single pulse of 1300 V for 30 ms. Cells were seeded on 24 mm diameter coverslip in a 6-well plate with 2 ml EGM+.

HUVECs (Lonza,P1052, Cat #C2519A) were cultured in Endothelial Cell Growth Medium-2 Bullet Kit (CC-3162, Lonza) with 100 U/ml penicillin (Thermo Fisher Scientific) and 100 μg/ml streptomycin (Thermo Fisher Scientific) at 37°C in 5% CO_2_. Culture dishes and coverslips were coated with fibronectin (30 μg/ml, Sanquin) in PBS 30 min prior to cell seeding.

### Neutrophil transendothelial migration

cbBOECs were cultured in a gelatin coated Ibidi μ-slide (VI0.4 Ibidi). Polymorphonuclear neutrophils were isolated from whole blood, donated by healthy adults, as described previously (Heemskerk et al., 2016). Neutrophils were kept at room temperature for a maximum of 4 h. At 20–30 min prior to the experiment, neutrophils were activated by incubation at 37°C. Sequentially, 10^6^ neutrophils were injected in a perfusion system. The perfusion system with HEPES buffer and a shear flow of 0.8 dyne/cm^2^ was used as described previously (Heemskerk et al., 2016). Spinning disk microscopy was performed as described below.

### Spinning disk microscopy

Cells were imaged with a Nikon Ti-E microscope equipped with a Yokogawa CSU X-1 spinning disk unit, a 60× objective (Plan Apo VC, oil, DIC, NA 1.4), Perfect Focus System and the Nikon NIS elements software. Images were acquired with a Andor iXon 897 EMCCD camera. CFPs were imaged using a 440 nm laser line, a triple dichroic mirror (440, 514, 561 nm) and a 460–500 nm emission filter. GFPs were imaged using a 488 nm laser line, a triple dichroic mirror (405, 488, 561 nm) and a 500 nm long pass emission filter. RFPs were imaged using a 561 nm laser line, a triple dichroic mirror (405, 488, 561 nm) and a 600–660 nm emission filter. HeLa cells were imaged between 24 to 48 h after transfection in an Attofluor cell chamber (Thermo Fisher Scientific) in 1 ml of microscopy medium (20 mM HEPES pH 7.4, 137 mM NaCl, 5.4 mM KCl, 1.8 mM CaCl2, 0.8 mM MgCl2 and 20 mM glucose) at 37°C. To measure the change in cytosolic intensity, HeLa cells were stimulated with 100 μM histamine and, if applicable, with 10 μM pyrilamine. The time trace of the cytosolic intensity of the rGBD sensor showed that the intensity stabilizes ∼1 min after histamine addition. That allowed to compare more cells with a higher throughput than by only comparing the cytosolic intensity before histamine addition to the cytosolic intensity 5 min after stimulation. For chemogenetic experiments, HeLa cells were stimulated with 100 nM rapamycin (LC Laboratories). Neutrophil transmigration time lapse movies were acquired with a 2×2 tile scan at 37°C with 5% CO_2_.

### Widefield microscopy

Dividing cells were imaged at a Nikon Ti-E widefield microscope, equipped with a 60× oil objective (Plan Apo λ 60× oil), a Lumencor Spectra X light source, the Perfect Focus System, a camera (Hamamatsu C11440-22C SN:100256) and Nikon NIS elements software. HeLa cells were imaged in DMEM+FCS at 37°C and 5% CO_2_ in an Attofluor cell chamber in a humidified environment. RFP was imaged with an excitation wavelength of 550/15 nm and emission light was detected at 570–616 nm with an emission filter of 593/46 in combination with a dichroic mirror (transmission at 411–452, 485–541, 567–621 and 656–793 nm).

### TIRF microscopy

Cells were imaged with a Nikon Ti-E microscope equipped with a motorized TIRF Illuminator unit, a 60× TIRF objective (60× Plan Apo, Oil DIC N2, NA=1.49, WD=120 μm) and Perfect Focus System. Images were acquired with an Andor iXon 897 EMCCD camera and the Nikon NIS elements software. mTurquoise2 was imaged using the 440 nm laser line in combination with a tri split dichroic mirror (440, 488 and 561 nm). DimericTomato was imaged using the 561 nm laser line in combination with a quad split dichroic mirror (405, 488, 561 and 640 nm) and a dual band pass emission filter (515–545 nm and 600–650 nm). BOECs stably expressing dimericTomato-2xrGBD and mTurquoise2-CaaX were imaged in an Attofluor cell chamber in 1 ml EGM+ at 37°C and 5% CO_2_. To measure Rho activity in primary cells, BOECs were stimulated with 1 U/ml human α-thrombin (HCT-0020, Haematologic technologies) diluted in PBS.

### Lattice light sheet microscopy

The lattice light sheet microscope located at the Advanced Imaging Center (AIC) at the Janelia Research Campus of the Howard Hughes Medical Institute (HHMI) (Chen et al., 2014) was used. HUVECs stably expressing dTomato-2xrGBD and mTurquoise2-CaaX were cultured on fibronectin-coated 5 mm round glass coverslips (Warner Instruments, Catalog # CS-5R) for 2 days. Cells were imaged at 37°C in the presence of 5% CO_2_ in HEPES buffer (132 mM NaCl_2_, 20 mM HEPES, 6 mM KCl_2_, 1 mM MgSO_4_•7H_2_O and 1.2 mM K_2_HPO_4_•3H_2_O at pH 7.4), supplemented with 1 mM CaCl_2_, 0.5% Albuman (Sanquin Reagents, The Netherlands) and 1 g/l D-glucose. Illumination was undertaken using 445 nm and 560 nm diode lasers (MPB Communications), acousto-optic tunable filter (AOTF) transmittance and 100 mW initial box power and an excitation objective (Special Optics, 0.65 NA, 3.74-mm WD). Fluorescence detection was done via a detection objective (Nikon, CFI Apo LWD 25XW, 1.1 NA) and a sCMOS camera (Hamamatsu Orca Flash 4.0 v2). Point-spread functions were measured using 200 nm tetraspeck beads (Invitrogen cat# T7280) for each wavelength. Data was deskewed and deconvolved as described previously (Chen et al., 2014).

### Data analysis

Raw microscopy images were analyzed in FIJI (Schindelin et al., 2012). Intensity time traces were generated by background correcting all images, drawing a region of interest (ROI) and measuring the mean gray value for this region for each frame. To measure the cell area, a ROI was created based on a binary image generated with Huang background-based thresholding (standard option in ImageJ) for each frame in the RFP channel. The mean gray value or cell area, respectively, was normalized by dividing each value by the value of the first frame, to account for differences in the initial intensity. Plots for Figs 1, 2, 5 and 6 were generated with the PlotTwist web tool (Goedhart, 2020).

The change in cytosolic intensity was measured by background correcting the images, drawing an ROI in the cytosol and measuring the mean gray value in this region. Then the ratio of mean gray value pre histamine stimulation to the mean gray value post histamine stimulation was calculated and plotted as a percentage using PlotsOfData (Postma and Goedhart, 2019). Scatter dot plots displaying the data points and their median were generated in this way for Figs 1, 4 and 6.

To determine the nuclear colocalization of the sensor with active Rho GTPases, an ROI of the nucleus was defined by manually thresholding the signal of H2A-mTurquoise2-RhoGTPase in the CFP channel. The region of interest for the cytosol was created by enlarging the nucleus region of interest by 1 μm and subtracting the region of interest of the nucleus from this. The two regions of interest were used to measure the mean gray value of the sensor in the RFP channel in the nucleus and the cytosol. The ratio of mean nuclear intensity to mean cytosolic intensity was calculated. Plots of these nuclear to cytosolic intensity were generated for Figs 3 and 4 with PlotsOfData (Postma and Goedhart, 2019).

To generate a cross section from the lattice light sheet data, slices 74–77 were averaged and collected in one stack.

The kymograph was created with the multi kymograph function in ImageJ using a linewidth of 1 at the line indicated in the image. The ImageJ LUTs mpl-magma or mpl-inferno were used to depict Rho sensor fluorescence intensity, where brighter yellow colors indicate higher fluorescence intensity.

### Statistical analysis

Datasets that are based on a single biological replicate are representative of multiple experiments. Statistical analysis was performed in the web tool PlotsOfData and PlotTwist (Goedhart, 2020; Postma and Goedhart, 2019). 95% confidence intervals were calculated by bootstrapping. The sample size depends on transfection efficiency and was not predefined. Cells with unusual morphology were excluded from analysis. Each dataset, represented in a dotplot, contains measurements for at least 10 individual cells.

## Supplementary Material

Supplementary information

Reviewer comments

## References

[JCS258823C1] Augsten, M., Pusch, R., Biskup, C., Rennert, K., Wittig, U., Beyer, K., Blume, A., Wetzker, R., Friedrich, K. and Rubio, I. (2006). Live-cell imaging of endogenous Ras-GTP illustrates predominant Ras activation at the plasma membrane. *EMBO Rep.* 7, 46-51. 10.1038/sj.embor.7400560 16282985 PMC1369223

[JCS258823C2] Bement, W. M., Leda, M., Moe, A. M., Kita, A. M., Larson, M. E., Golding, A. E., Pfeuti, C., Su, K.-C., Miller, A. L., Goryachev, A. B. et al. (2015). Activator – inhibitor coupling between Rho signalling and actin assembly makes the cell cortex an excitable medium. *Nat. Cell Biol.* 17, 1471-1483. 10.1038/ncb3251 26479320 PMC4849138

[JCS258823C3] Benink, H. A. and Bement, W. M. (2005). Concentric zones of active RhoA and Cdc42 around single cell wounds. *J. Cell Biol.* 168, 429-439. 10.1083/jcb.200411109 15684032 PMC2171735

[JCS258823C4] Bery, N., Keller, L., Soulié, M., Gence, R., Iscache, A.-L., Cherier, J., Cabantous, S., Sordet, O., Lajoie-Mazenc, I., Pedelacq, J.-D. et al. (2019). A targeted protein degradation cell-based screening for nanobodies selective toward the cellular RHOB GTP-bound conformation. *Cell Chem. Biol.* 26, 1544-1558.e6. 10.1016/j.chembiol.2019.08.009 31522999

[JCS258823C5] Bos, J. L., Rehmann, H. and Wittinghofer, A. (2009). GEFs and GAPs: critical elements in the control of small G proteins. *Cell* 16, 374-383. 10.1016/j.cell.2007.05.018 17540168

[JCS258823C6] Burridge, K. and Wennerberg, K. (2004). Rho and rac take center stage. *Cell* 116, 167-179. 10.1016/S0092-8674(04)00003-0 14744429

[JCS258823C7] Chen, B.-C., Legant, W. R., Wang, K., Shao, L., Milkie, D. E., Davidson, M. W., Janetopoulos, C., Wu, X. S., Hammer, J. A., III, Liu, Z. et al. (2014). Lattice light-sheet microscopy: Imaging molecules to embryos at high spatiotemporal resolution. *Science* 346, 1257998. 10.1126/science.1257998 25342811 PMC4336192

[JCS258823C8] Chin, J. X., Chung, B. K.-S. and Lee, D.-Y. (2014). Codon Optimization OnLine (COOL): a web-based multi-objective optimization platform for synthetic gene design. *Bioinformatics* 30, 2210-2212. 10.1093/bioinformatics/btu192 24728853

[JCS258823C9] Davenport, N. R., Sonnemann, K. J., Eliceiri, K. W., Bement, W. M. and Drubin, D. G. (2016). Membrane dynamics during cellular wound repair. *Mol. Biol. Cell* 27, 2272-2285. 10.1091/mbc.E16-04-0223 27226483 PMC4945144

[JCS258823C10] Dewitt, S., Darley, R. L. and Hallett, M. B. (2009). Translocation or just location? Pseudopodia affect fluorescent signals. *J. Cell Biol.* 184, 197-203. 10.1083/jcb.200806047 19171754 PMC2654297

[JCS258823C11] Fritz, R. D., Letzelter, M., Reimann, A., Martin, K., Fusco, L., Ritsma, L., Ponsioen, B., Fluri, E., Schulte-Merker, S., Van Rheenen, J. et al. (2013). A versatile toolkit to produce sensitive FRET biosensors to visualize signaling in time and space. *Sci. Signal.* 6, rs12. 10.1126/scisignal.2004135 23882122

[JCS258823C12] Garcia-Mata, R., Boulter, E. and Burridge, K. (2011). The 'invisible hand': regulation of RHO GTPases by RHOGDIs. *Nat. Rev. Mol. Cell Biol.* 12, 493-504. 10.1038/nrm3153 21779026 PMC3260518

[JCS258823C13] Gillingham, A. K., Bertram, J., Begum, F. and Munro, S. (2019). In vivo identification of GTPase interactors by mitochondrial relocalization and proximity biotinylation. *eLife* 8, e45916. 10.7554/eLife.45916 31294692 PMC6639074

[JCS258823C14] Goedhart, J. (2020). Plottwist: a web app for plotting and annotating continuous data. *PLoS Biol.* 18, e3000581. 10.1371/journal.pbio.3000581 31929523 PMC6980690

[JCS258823C15] Goulden, B. D., Pacheco, J., Dull, A., Zewe, J. P., Deiters, A. and Hammond, G. R. V. (2019). A high-avidity biosensor reveals plasma membrane PI(3,4)P2 is predominantly a class I PI3K signaling product. *J. Cell Biol.* 218, 1066-1079. 10.1083/jcb.201809026 30591513 PMC6400549

[JCS258823C16] Graessl, M., Koch, J., Calderon, A., Kamps, D., Banerjee, S., Mazel, T., Schulze, N., Jungkurth, J. K., Patwardhan, R., Solouk, D. et al. (2017). An excitable Rho GTPase signaling network generates dynamic subcellular contraction patterns. *J. Cell Biol.* 216, 4271-4285. 10.1083/jcb.201706052 29055010 PMC5716289

[JCS258823C17] Greenwald, E. C., Mehta, S. and Zhang, J. (2018). Genetically encoded fluorescent biosensors illuminate the spatiotemporal regulation of signaling networks. *Chem. Rev.* 118, 11707-11794. 10.1021/acs.chemrev.8b00333 30550275 PMC7462118

[JCS258823C18] Heemskerk, N., Van Rijssel, J. and Van Buul, J. D. (2014). Rho-GTPase signaling in leukocyte extravasation: an endothelial point of view. *Cell Adhes. Migr.* 8, 67-75. 10.4161/cam.28244 PMC4049863 24621576

[JCS258823C19] Heemskerk, N., Schimmel, L., Oort, C., Van Rijssel, J., Yin, T., Ma, B., Van Unen, J., Pitter, B., Huveneers, S., Goedhart, J. et al. (2016). F-actin-rich contractile endothelial pores prevent vascular leakage during leukocyte diapedesis through local RhoA signalling. *Nat. Commun.* 7, 10493. 10.1038/ncomms10493 26814335 PMC4737874

[JCS258823C20] Ito, H., Morishita, R. and Nagata, K. (2018). Functions of Rhotekin, an effector of Rho GTPase, and its binding partners in mammals. *Int. J. Mol. Sci.* 19, 2121. 10.3390/ijms19072121 PMC6073136 30037057

[JCS258823C21] Jiang, T. and Harris, T. J. C. (2019). Par-1 controls the composition and growth of cortical actin caps during *Drosophila* embryo cleavage. *J. Cell Biol.* 218, 4195-4214. 10.1083/jcb.201903152 31641019 PMC6891076

[JCS258823C22] Keller, L., Bery, N., Tardy, C., Ligat, L., Favre, G., Rabbitts, T. H. and Olichon, A. (2019). Selection and characterization of a nanobody biosensor of GTP-bound RHO activities. *Antibodies* 8, 8. 10.3390/antib8010008 PMC6640709 31544814

[JCS258823C23] Lachmann, S., Jevons, A., de Rycker, M., Casamassima, A., Radtke, S., Collazos, A. and Parker, P. J. (2011). Regulatory domain selectivity in the cell-type specific PKN-dependence of cell migration. *PLoS ONE* 6, e21732. 10.1371/journal.pone.0021732 21754995 PMC3130767

[JCS258823C24] Lawson, C. D. and Ridley, A. J. (2018). Rho GTPase signaling complexes in cell migration and invasion. *J. Cell Biol.* 217, 447-457. 10.1083/jcb.201612069 29233866 PMC5800797

[JCS258823C25] Lim, W. G., Tan, B. J., Zhu, Y., Zhou, S., Armstrong, J. S., Li, Q. T., Dong, Q., Chan, E., Smith, D., Verma, C. et al. (2006). The very C-terminus of PRK1/PKN is essential for its activation by RhoA and downstream signaling. *Cell. Signal.* 18, 1473-1481. 10.1016/j.cellsig.2005.11.009 16427251

[JCS258823C26] Maesaki, R., Ihara, K., Shimizu, T., Kuroda, S., Kaibuchi, K. and Hakoshima, T. (1999). The structural basis of Rho effector recognition revealed by the crystal structure of human RhoA complexed with the effector domain of PKN/PRK1. *Mol. Cell* 4, 793-803. 10.1016/S1097-2765(00)80389-5 10619026

[JCS258823C27] Martin-Ramirez, J., Hofman, M., Van Den Biggelaar, M., Hebbel, R. P. and Voorberg, J. (2012). Establishment of outgrowth endothelial cells from peripheral blood. *Nat. Protoc.* 7, 1709-1715. 10.1038/nprot.2012.093 22918388

[JCS258823C28] Mason, F. M., Xie, S., Vasquez, C. G., Tworoger, M. and Martin, A. C. (2016). RhoA GTPase inhibition organizes contraction during epithelial morphogenesis. *J. Cell Biol.* 214, 603-617. 10.1083/jcb.201603077 27551058 PMC5004446

[JCS258823C29] Mehta, S. and Zhang, J. (2011). Reporting from the field: genetically encoded fluorescent reporters uncover signaling dynamics in living biological systems. *Annu. Rev. Biochem.* 80, 375-401. 10.1146/annurev-biochem-060409-093259 21495849 PMC4384825

[JCS258823C30] Michaelson, D., Silletti, J., Murphy, G., D'Eustachio, P., Rush, M. and Philips, M. R. (2001). Differential localization of Rho GTPases in live cells: regulation by hypervariable regions and RhoGDI binding. *J. Cell Biol.* 152, 111-126. 10.1083/jcb.152.1.111 11149925 PMC2193662

[JCS258823C31] Miyawaki, A. and Niino, Y. (2015). Molecular spies for bioimaging—fluorescent protein-based probes. *Mol. Cell* 58, 632-643. 10.1016/j.molcel.2015.03.002 26000848

[JCS258823C32] Munjal, A., Philippe, J.-M., Munro, E. and Lecuit, T. (2015). A self-organized biomechanical network drives shape changes during tissue morphogenesis. *Nature* 524, 351-355. 10.1038/nature14603 26214737

[JCS258823C33] O'Neill, P. R., Castillo-Badillo, J. A., Meshik, X., Kalyanaraman, V., Melgarejo, K. and Gautam, N. (2018). Membrane flow drives an adhesion-independent amoeboid cell migration mode. *Dev. Cell* 46, 9-22.e4. 10.1016/j.devcel.2018.05.029 29937389 PMC6048972

[JCS258823C34] Pertz, O. (2010). Spatio-temporal Rho GTPase signaling – where are we now? *J. Cell Sci.* 123, 1841-1850. 10.1242/jcs.064345 20484664

[JCS258823C35] Pertz, O. and Hahn, K. M. (2004). Designing biosensors for Rho family proteins — deciphering the dynamics of Rho family GTPase activation in living cells. *J. Cell Sci.* 117, 1313-1318. 10.1242/jcs.01117 15020671

[JCS258823C36] Piekny, A. J. and Glotzer, M. (2008). Anillin is a scaffold protein that links RhoA, actin, and myosin during cytokinesis. *Curr. Biol.* 18, 30-36. 10.1016/j.cub.2007.11.068 18158243

[JCS258823C37] Postma, M. and Goedhart, J. (2019). PlotsOfData — A web app for visualizing data together with their summaries. *PLoS Biol.* 17, e3000202. 10.1371/journal.pbio.3000202 30917112 PMC6453475

[JCS258823C38] Reid, T., Furuyashiki, T., Ishizaki, T., Watanabe, G., Watanabe, N., Fujisawa, K., Morii, N., Madaule, P. and Narumiya, S. (1996). Rhotekin, a new putative target for Rho bearing homology to a serine/threonine kinase, PKN, and rhophilin in the Rho-binding domain. *J. Biol. Chem.* 271, 13556-13560. 10.1074/jbc.271.23.13556 8662891

[JCS258823C39] Reinhard, N. R., Van Helden, S. F., Anthony, E. C., Yin, T., Wu, Y. I., Goedhart, J., Gadella, T. W. J. and Hordijk, P. L. (2016). Spatiotemporal analysis of RhoA/B/C activation in primary human endothelial cells. *Sci. Rep.* 6, 25502. 10.1038/srep25502 27147504 PMC4857094

[JCS258823C40] Ren, X.-D., Kiosses, W. B. and Schwartz, M. A. (1999). Regulation of the small GTP-binding protein Rho by cell adhesion and the cytoskeleton. *EMBO J.* 18, 578-585. 10.1093/emboj/18.3.578 9927417 PMC1171150

[JCS258823C41] Ridley, A. J. (2015). Rho GTPase signalling in cell migration. *Curr. Opin. Cell Biol.* 36, 103-112. 10.1016/j.ceb.2015.08.005 26363959 PMC4728192

[JCS258823C42] Ridley, A. J. and Hall, A. (1992). The small GTP-binding protein rho regulates the assembly of focal adhesions and actin stress fibers in response to growth factors. *Cell* 70, 389-399. 10.1016/0092-8674(92)90163-7 1643657

[JCS258823C43] Rossman, K. L., Der, C. J. and Sondek, J. (2005). GEF means go: turning on Rho GTPases with guanine nucleotide-exchange factors. *Nat. Rev. Mol. Cell Biol.* 6, 167-180. 10.1038/nrm1587 15688002

[JCS258823C44] Schindelin, J., Arganda-Carreras, I., Frise, E., Kaynig, V., Longair, M., Pietzsch, T., Preibisch, S., Rueden, C., Saalfeld, S., Schmid, B. et al. (2012). Fiji: an open-source platform for biological-image analysis. *Nat. Methods* 9, 676-682. 10.1038/nmeth.2019 22743772 PMC3855844

[JCS258823C45] Sun, L., Guan, R., Lee, I.-J., Liu, Y., Chen, M., Wang, J., Wu, J.-Q. and Chen, Z. (2015). Mechanistic insights into the anchorage of the contractile ring by anillin and mid1. *Dev. Cell* 33, 413-426. 10.1016/j.devcel.2015.03.003 25959226 PMC4449299

[JCS258823C46] Timmerman, I., Heemskerk, N., Kroon, J., Schaefer, A., van Rijssel, J., Hoogenboezem, M., van Unen, J., Goedhart, J., Gadella, T. W. J., Yin, T. et al. (2015). A local VE-cadherin and Trio-based signaling complex stabilizes endothelial junctions through Rac1. *J. Cell Sci.* 128, 3041-3054. 10.1242/jcs.179424 26116572

[JCS258823C47] Van Unen, J., Reinhard, N. R., Yin, T., Wu, Y. I., Postma, M., Gadella, T. W. J. and Goedhart, J. (2015). Plasma membrane restricted RhoGEF activity is sufficient for RhoA-mediated actin polymerization. *Sci. Rep.* 5, 14693. 10.1038/srep14693 26435194 PMC4592971

[JCS258823C48] Van Unen, J., Rashidfarrokhi, A., Hoogendoorn, E., Postma, M., Gadella, T. W. J. and Goedhart, J. (2016). Quantitative single-cell analysis of signaling pathways activated immediately downstream of histamine receptor subtypes. *Mol. Pharmacol.* 90, 162-176. 10.1124/mol.116.104505 27358232

[JCS258823C49] Verma, V. and Maresca, T. J. (2019). Microtubule plus-ends act as physical signaling hubs to activate RhoA during cytokinesis. *eLife* 8, e38968. 10.7554/eLife.38968 30758285 PMC6398982

[JCS258823C50] Watanabe, N. and Mitchison, T. J. (2002). Single-molecule speckle analysis of actin filament turnover in lamellipodia. *Science* 295, 1083-1086. 10.1126/science.1067470 11834838

[JCS258823C51] Wheeler, A. P. and Ridley, A. J. (2004). Why three Rho proteins? RhoA, RhoB, RhoC, and cell motility. *Exp. Cell Res.* 301, 43-49. 10.1016/j.yexcr.2004.08.012 15501444

[JCS258823C52] Yoo, S. K., Deng, Q., Cavnar, P. J., Wu, Y. I., Hahn, K. M. and Huttenlocher, A. (2010). Differential regulation of protrusion and polarity by PI(3)K during neutrophil motility in live zebrafish. *Dev. Cell* 18, 226-236. 10.1016/j.devcel.2009.11.015 20159593 PMC2824622

[JCS258823C53] Zilberman, Y., Alieva, N. O., Miserey-Lenkei, S., Lichtenstein, A., Kam, Z., Sabanay, H. and Bershadsky, A. (2011). Involvement of the Rho-mDia1 pathway in the regulation of Golgi complex architecture and dynamics. *Mol. Biol. Cell* 22, 2900-2911. 10.1091/mbc.e11-01-0007 21680709 PMC3154885

