# Supplementary material for: Visualizing endogenous Rho activity with an improved localization-based, genetically encoded biosensor
Source: J Cell Sci. 2021 Sep 8;134(17):jcs258823. doi: 10.1242/jcs.258823 (PMC8445605; doi:10.1242/jcs.258823)
Supplement: Supplementary information [file joces-134-258823-s1.pdf]

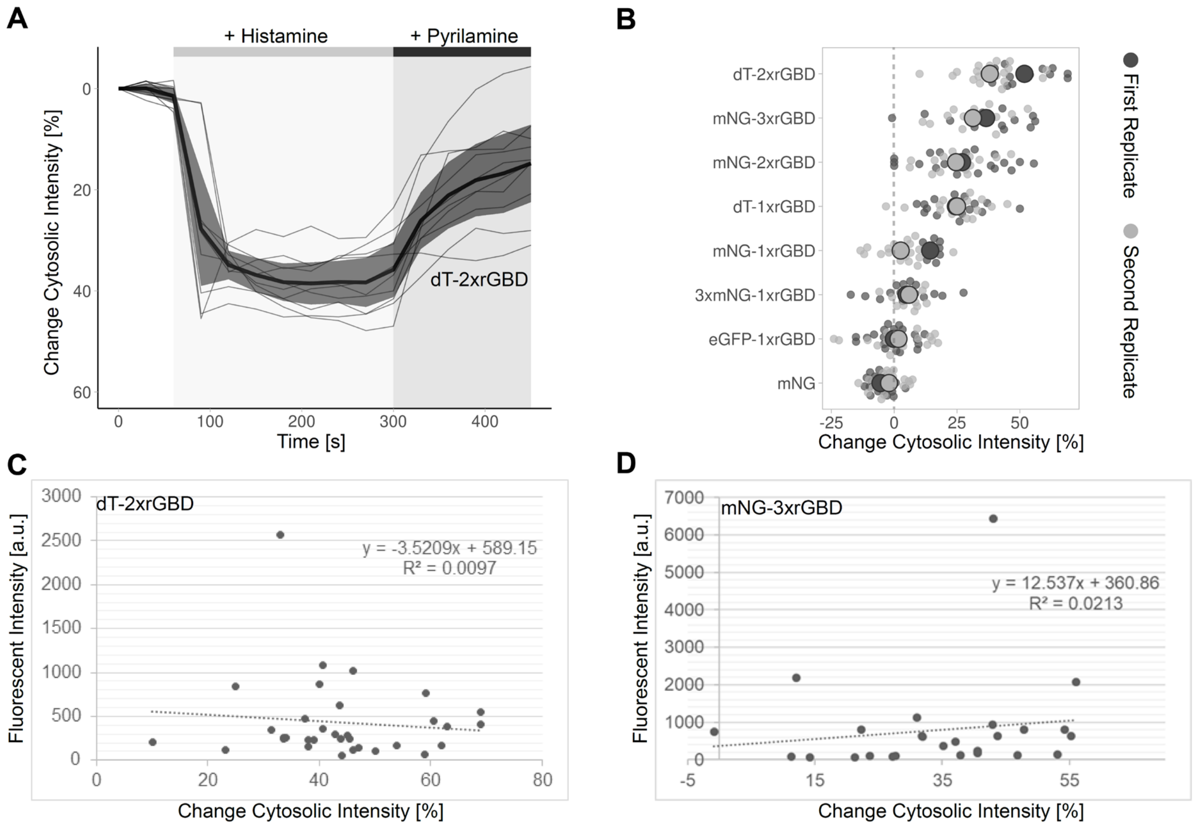

Fig. S1. **A**) Time traces of the change in cytosolic intensity for HeLa cells expressing the CMVdel-dimericTomato-2xrGBD Rho sensor and H1R which were stimulated with 100 µM histamine after 60 s and 10 µM pyrilamine after 300 s. The thick black line represents the median and the black ribbon indicates the 95 % confidence interval of the median. The experiment was performed once. The number of analyzed cells is 10. **B**) Change in cytosolic intensity for mNeonGreen, eGFP-1xrGBD, 3xmNeonGreen-1xrGBD, mNeonGreen-1xrGBD/ -2xrGBD/ -3xrGBD, and dimericTomato-1xrGBD/ -2xrGBD in Hela cells expressing H1R, upon stimulation with 100 µM histamine as shown in Fig 1B. This plot shows the reproducibility between replicates. Each small dot represents an individual cell, black dots are measurements from the first replicate and grey dots from the second. The larger dots represent the median per replicate. The grey, dashed line indicates no change in cytosolic intensity. The experiment was performed twice with two individual transfections. The number of cells per condition is: 3xmNG-1xrGBD=27, dT-1xrGBD=32, dT- 2xrGBD=33, eGFP-1xrGBD=40, mNG=39, mNG-1xrGBD=28, mNG-2xrGBD=34, mNG-3xrGBD=26 **C**) Plot of the correlation between fluorescent intensity and change in cytosolic intensity for cells expressing dimericTomato- 2xrGBD for the data shown in Fig. 1B. The general linear model (top right corner) was fit to the data and is indicated as a dashed line. **D**) Plot of the correlation between fluorescent intensity and change in cytosolic intensity for cells expressing mNeonGreen-3xrGBD for the data shown in Fig. 1B. The general linear model (top right corner) was fit to the data and is indicated as a dashed line.

Journal of Cell Science • Supplementary information

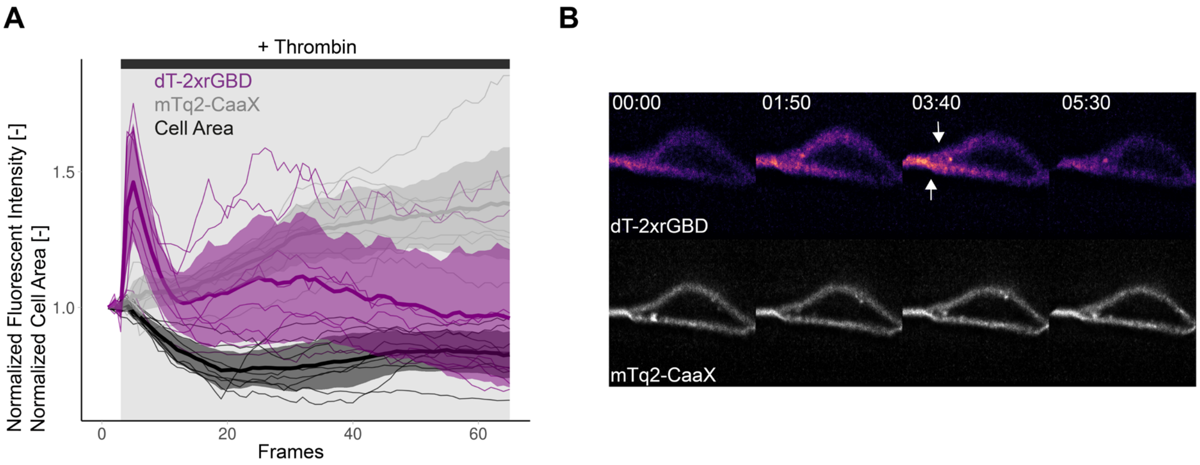

Fig. S2. A) Time trace of the normalized fluorescent intensity for the dimericTomato-2xrGBD sensor (purple) and the mTq2-CaaX membrane label (grey) stably expressed in cbBOECs, imaged with TIRF microscopy, stimulated with 1 U/ml human α-thrombin after 3 frames. Measurements of the normalized cell area are shown in black. Thick lines represent the median and ribbons indicate the 95 % confidence interval. The experiment was performed on two different days. The number of analyzed cells is 8, including the example shown in Fig. 6A,B. **B**) Lattice light sheet cross section images of a HUVEC stably expressing the dimericTomato-2xrGBD sensor (upper panel) and mTurquoise2-CaaX(lowerpanel).Thecellwasstimulatedwith2.5µM nocodazole 10 min prior to the imaging. Arrows indicate dimericTomato-2xrGBD intensity increase at basal and apical plasma membrane.

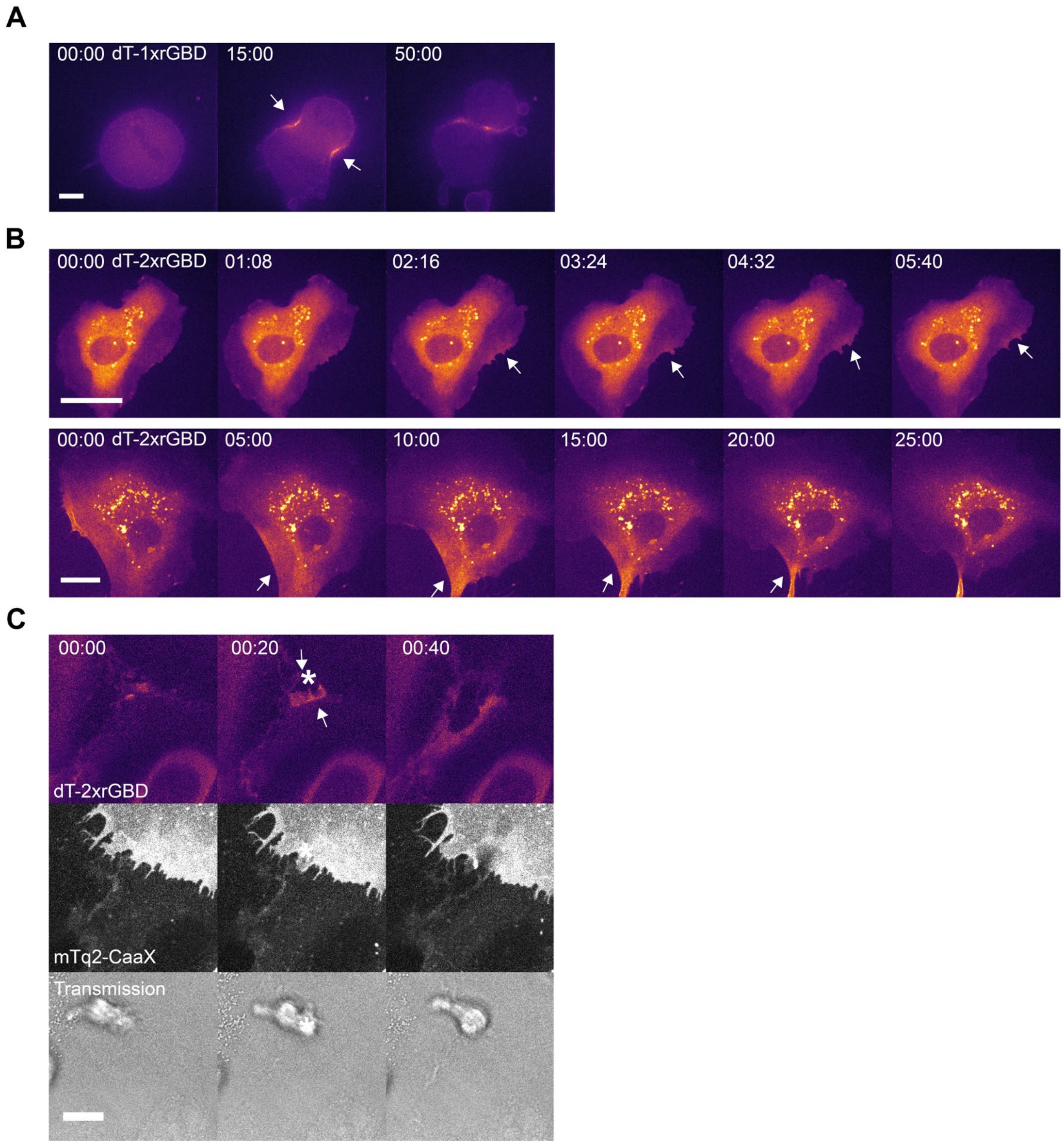

Fig. S3. A) Spinning disk images of a Hela cell expressing dimericTomato-1xrGBD sensor going through cell division. Arrows indicate intensity increase at the location that will become the cleavage furrow. Scale bar: 10 µm **B**) Spinning disk images of a BOEC transiently expressing dimericTomato-2xrGBD sensor randomly migrating. Arrows indicate contracting cell edge with increased sensor intensity. Scale bar: 25 µm **C**) Spinning disk still images of a transendothelial migration event with cbBOECs grown in to a monolayer, expressing the dimericTomato-2xrGBD sensor (upper panel) and the membrane marker mTurquoise2-CaaX (middle panel). The transmigrating neutrophil is visible in the transmission panel; its position is indicated with an asterisk. The dashed line indicates where the neutrophil is underneath the cbBOECs. Arrows indicate increased dimericTomato-2xrGBD sensor intensity around the migration pore. Scale bar: 10 µm

Journal of Cell Science • Supplementary information

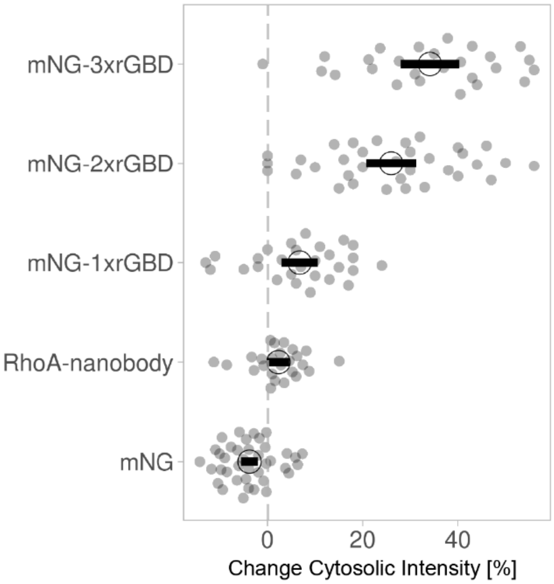

Fig. S4. Change in cytosolic intensity for mNeonGreen, HaloTag-anti RhoA nanobody, mNeonGreen-1xrGBD/ -2xrGBD/ - 3xrGBD in Hela cells expressing H1R, upon stimulation with 100 µM histamine. The black circle indicates the mean and a horizontal bar indicates the 95% confidence interval. The grey dashed line indicates no change in cytosolic intensity. The number of cells per condition is: mNG=39, mNG-1xrGBD=28, mNG-2xrGBD=34, mNG-3xrGBD=26, RhoA-nanobody=25.

Journal of Cell Science • Supplementary information

Table S1. PCR primers for insert amplification.

PCR product	Template	Primer sequence
**2nd rGBD**	4914 CMVdel mNeonGreen 1xrGBD	FW 5’-TATATATGTACATCCCCCTCGAGTCCATCCTGGAGGACCTCAAT-3’
		RV 5’-TATATAGTCGACGCCTGCCTGTCTTCTCCAGCAC-3’
**3rd rGBD**	4914 CMVdel mNeonGreen 1xrGBD	FW 5’-TATATATGTACATCCCCCTCGAGTCCATCCTGGAGGACCTCAAT-3’
		RV 5’-TATATAGTCGACCCTGCCTGTCTTCTCCAGCAC-3’
**1st aGBD**	1195 eGFP anillin	FW 5’-GCTGTACAGCCTCGAGCAAGTTAATATCAAACAGA-3’
		RV 5’-GCGGATCCTTACCTCTGAGGTCCTTCGTT-3’
**n+1 aGBD**	1195 eGFP anillin	FW 5’-GCTGTACAGCCTCGAGCAAGTTAATATCAAACAGA-3’
		RV 5’- GCGCGTCGACTTTGGATGGCATAAATTC-3’
**1st pGBD**	pGBD gblock	FW 5’-GCTGTACAGCCTCGAGGGGGTACAGCAGCAG-3’
		RV 5’-GCGGATCCTTAAAGCACCACGTGGGC-3’
**n+1 pGBD**	pGBD gblock	FW 5’-GCTGTACAGCCTCGAGGGGGTACAGCAGCAG-3’
		RV 5’-CGCGTCGACAAGCACCACGTGGGC-3’
**CDC42-G12V-Δ-CaaX**	5412 CDC42-G12V	FW 5’-TATATGTACAAGTCCATGCAGAC-3’
		RV 5’-TATAGCGGCCGCTTAGC**T**CTTCCTTTTGGGTTGAG-3’
**RAC1-G12V-Δ-CaaX**	5422 RAC1-G12V	FW 5’-TATATGTACAAGTCCATGCAGGCC-3’
		RV 5’-TATAGCGGCCGCTTAGC**T**TTTTCTCTTCCTCTTCTTC-3’
**RHOA-G14V-Δ-CaaX**	1944 RHOA-G14V	FW 5’-TATATGTACAAGTCCATGGCTG-3’
		RV 5’-TATAGCGGCCGCTTAGC**T**CCCAGATTTTTTCTTCCC-3’

Journal of Cell Science • Supplementary information

Table S2. Plasmid names and cloning strategy. The sequences of the plasmid maps are available here: https://dx.doi.org/10.5281/zenodo.5115592. The 4-digit number in the first column matches the filename of the plasmid sequence.

	NAME	BACK-BONE	RESTRICTION ENZYMES BACKBONE	INSERT	RESTRICTION ENZYMES INSERT
**4914**	CMVdel-mNeonGreen-1xrGBD	4900	AgeI, BsrGI	4907	AgeI, BsrGI
**5196**	CMVdel-dTomato-1xrGBD	4914	AgeI,BsrGI	4595	AgeI,BsrGI
**5215**	CMVdel-mScarlet-I-1xrGBD	4914	AgeI, BsrGI	4604	AgeI, BsrGI
**5214**	CMVdel-mTurquiose2-1xrGBD	4914	AgeI, BsrGI	3703	AgeI, BsrGI
**1199**	CMV-3xmNeonGreen-1xrGBD	3877	AgeI, BsrGI	2376	AgeI, BsrGI
**5380**	CMVdel-dTomato-2xrGBD	4915	AgeI, BsrGI	4595	AgeI, BsrGI
**4915**	CMVdel-mNeonGreen-2xrGBD	4914	BsrGI, AvaI	2nd rGBD	BsrGI, SalI
**4917**	CMVdel-mNeonGreen 3xrGBD	4915	BsrGI, AvaI	3rd rGBD	BsrGI, SalI
**5186**	CMVdel-mNeonGreen-anillin (AHD+PH)	1645	AgeI, BsrGI	3331	AgeI, BsrGI
**1641**	mTurquoise2-anillin(AHD+PH)	1195	AgeI, BsrGI	3703	AgeI, BsrGI
**5180**	CMVdel-mNeonGreen-1xaGBD(712-786)	5186	BsrGI, BamHI	1st aGBD	BsrGI, BamHI
**5181**	CMVdel-mNeonGreen-2xaGBD(712-786/801)	5180	BsrGI, AvaI	n+1 aGBD	BsrGI, SalI
**5182**	CMVdel-mNeonGreen-3xaGBD(712-786/ 801)	5181	BsrGI,AvaI	n+1 aGBD	BsrGI, SalI
**5183**	CMVdel-mNeonGreen-1xpGBD(30-100)t	5186	BsrGI, BamHI	1st pGBD	BsrGI, BamHI
**5184**	CMVdel-mNeonGreen-2xpGBD(30-100)	5183	BsrGI, AvaI	n+1 pGBD	BsrGI, SalI
**5197**	CMVdel-dTomato-2xpGBD(30-100)	5184	AgeI,BsrGI	4595	AgeI,BsrGI
**5185**	CMVdel-mNeonGreen-3xpGBD(30-100)	5184	BsrGI, AvaI	n+1 pGBD	BsrGI, SalI
**5431**	H2A-mTurquiose2-CDC42-G12V-ΔCaaX	5068	BsrGI, NotI	CDC42-G12V-ΔCaaX	BsrGI, NotI
**5432**	H2A-mTurquiose2-RAC1-G12V-ΔCaaX	5068	BsrGI, NotI	RAC1-G12V-ΔCaaX	BsrGI, NotI
**5434**	H2A-mTurquiose2-RHOA-G14V-ΔCaaX	5068	BsrGI, NotI	RHOA-G14V-ΔCaaX	BsrGI, NotI
**5712**	pLV-dTomato-2xrGBD	5147	EcoRV, ApaI	5200	EcoRV, ApaI

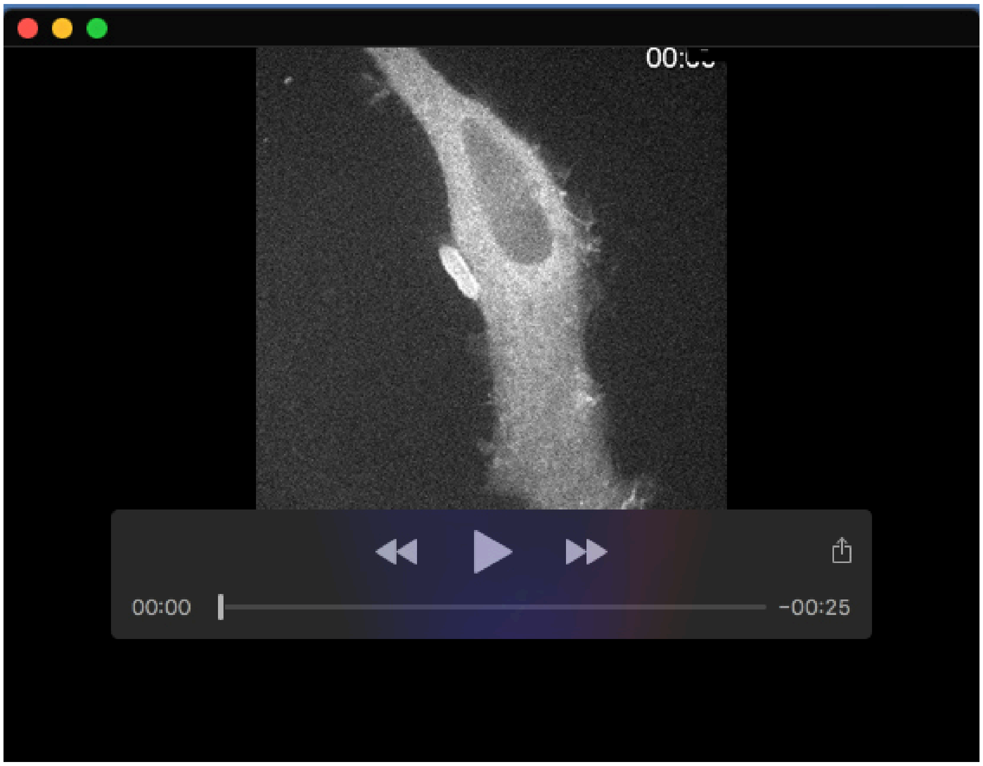

Movie 1. The dimericTomato2xrGBD Rho sensor relocating in a HeLa cell. Spinning disk time lapse movie of a HeLa cell expressing the CMVdel-dimericTomato-2xrGBD RhoA sensor and H1R (not shown) which was stimulated with 100 µM histamine after 150 s and 10 µM pyrilamine after 350 s. The time stamper represents min:s.

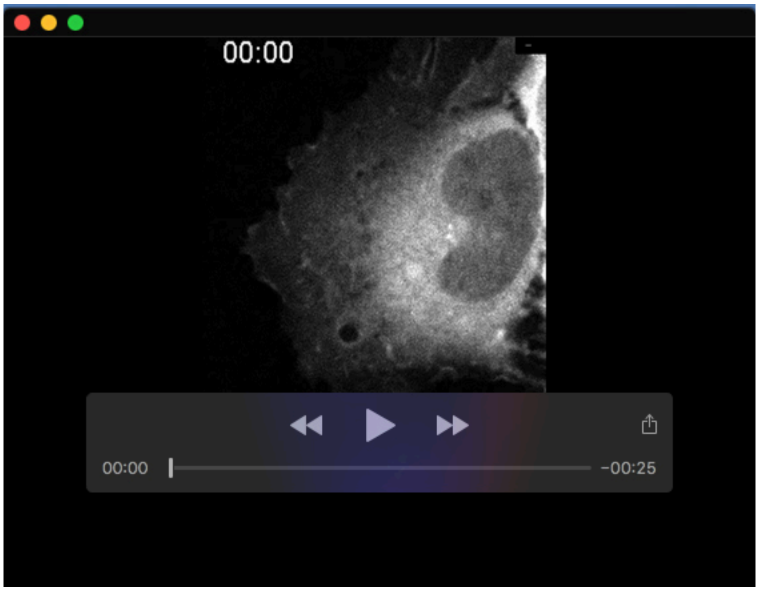

Movie 2. The mNeonGreen1xrGBD Rho sensor relocating in a HeLa cell. Spinning disk time lapse movie of a HeLa cell expressing the CMVdel-mNeonGreen-1xrGBD RhoA sensor and H1R (not shown) which was stimulated with 100 µM histamine after 150 s and 10 µM pyrilamine after 350 s. The time stamper represents min:s.

Journal of Cell Science • Supplementary information

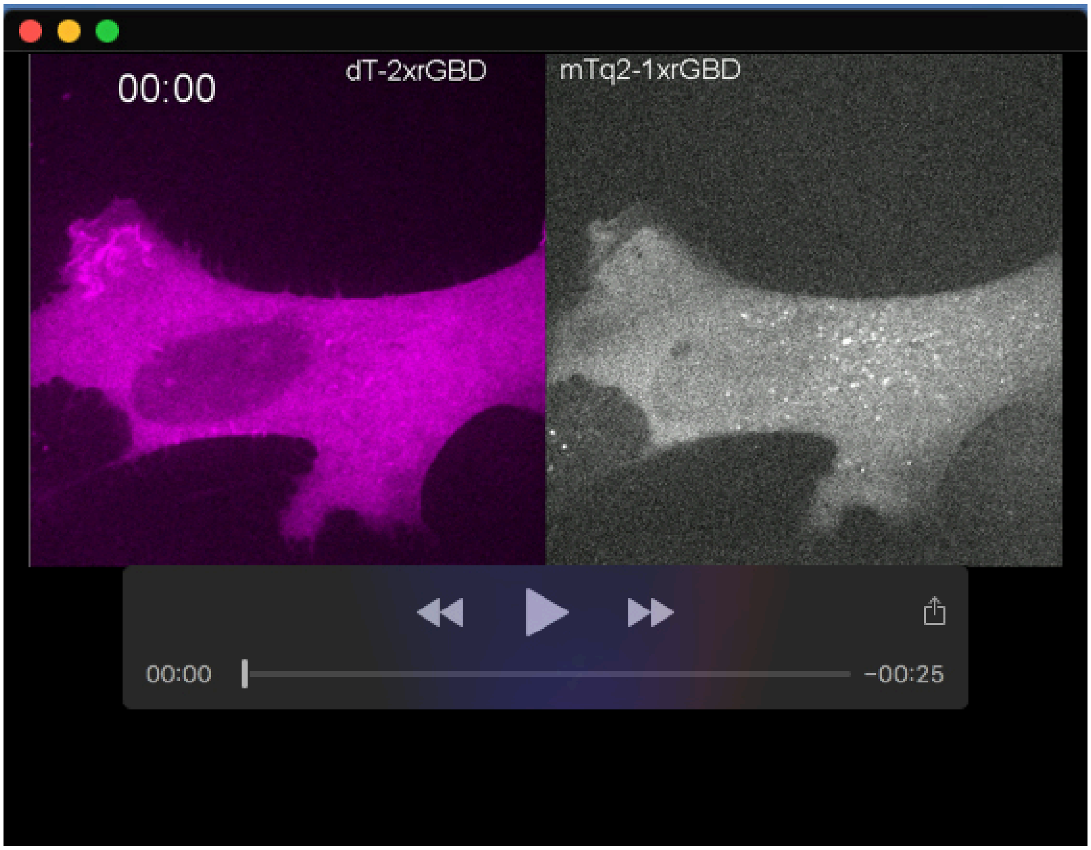

Movie 3. Direct comparison of dimericTomato-2xrGBD sensor and mTurquoise2-1xrGBD sensor in one HeLa cell. Spinning disk time lapse movie of a HeLa cell expressing H1R (not shown) and CMVdel-dimericTomato-2xrGBD (magenta) and CMVdel-mTurquoise2-1xrGBD (grey) stimulated with 100 µM histamine. The time stamper represents min:s.

Journal of Cell Science • Supplementary information

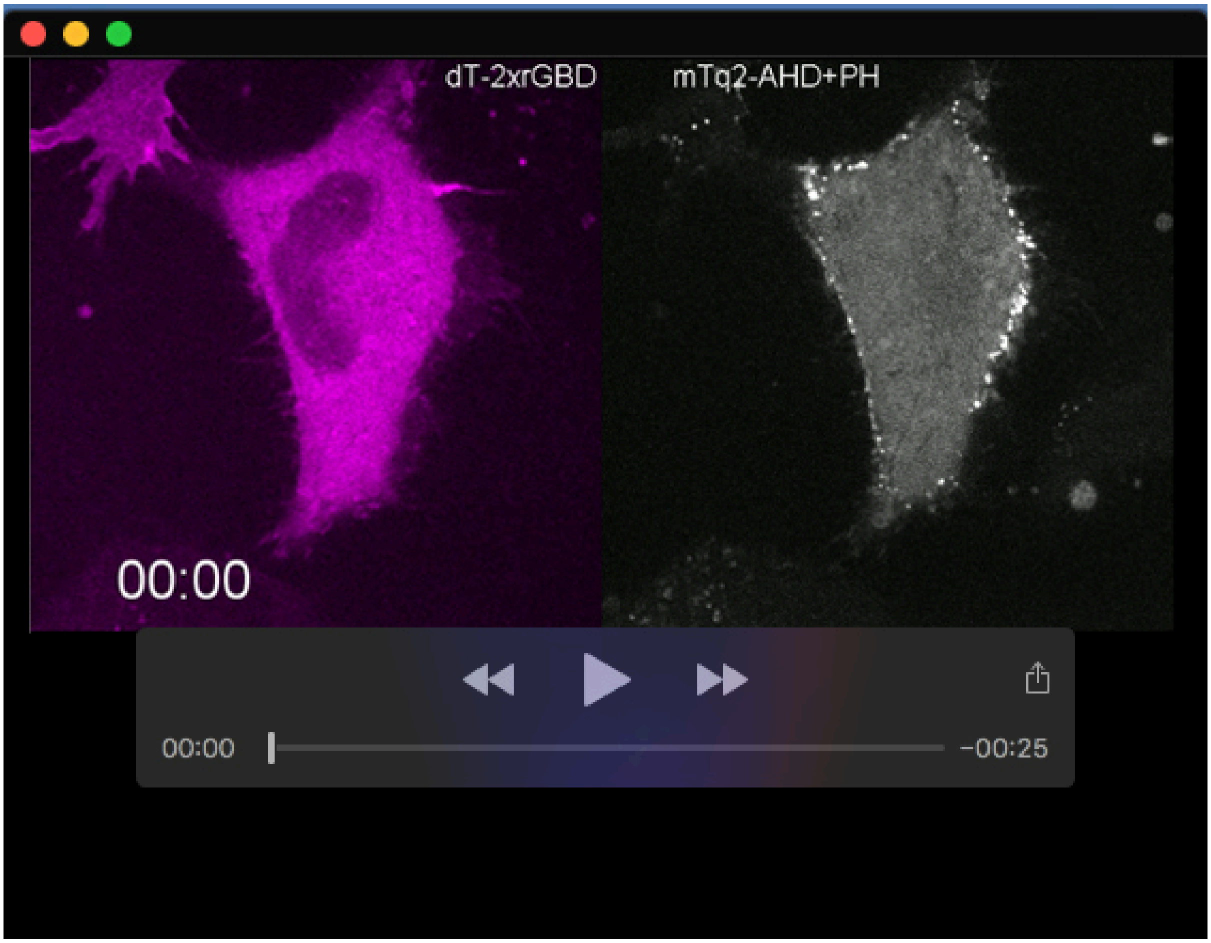

Movie 4. Direct comparison of dimericTomato-2xrGBD sensor and mTurquoise2-AHD+PH sensor in one HeLa cell. Spinning disk time lapse movie of a HeLa cell expressing H1R (not shown) and CMVdel-dimericTomato-2xrGBD (magenta) and mTurquoise2-AHD+PH (grey) stimulated with 100 µM histamine. The time stamper represents min:s.

Journal of Cell Science • Supplementary information

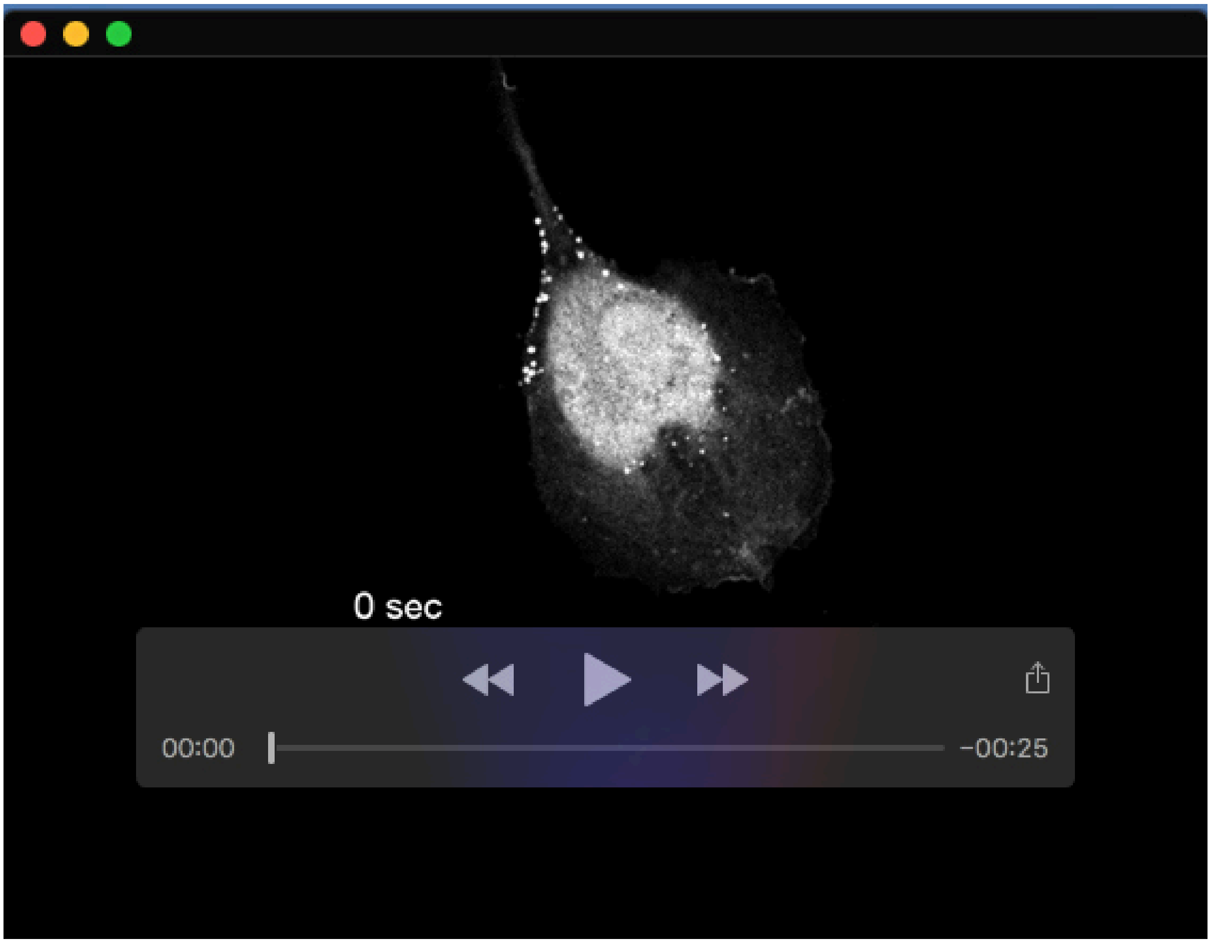

Movie 5. AHD+PH Rho sensor localizing in a punctate manner in an endothelial cell. Spinning disk time lapse movie of a HUVEC expressing the eGFP-AHD+PH sensor, stimulated with 1 U/ml human α-thrombin.

Journal of Cell Science • Supplementary information

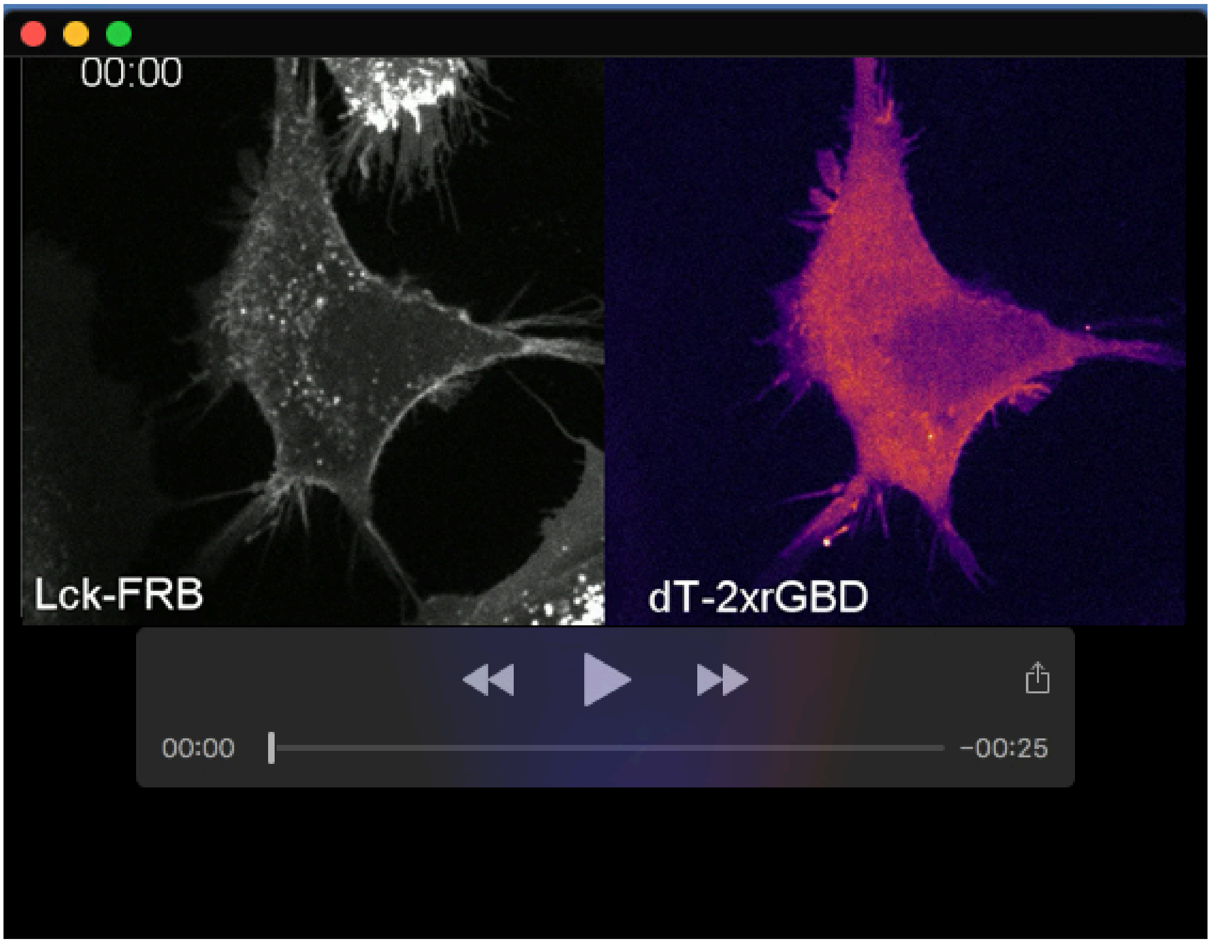

Movie 6. Membrane specific activation of Rho detected with the dimericTomato-2xrGBD sensor. Spinning disk time lapse movie of a HeLa cells expressing FRB anchored to the membrane (grey), FKBP-p63-DH (not shown) and the dimericTomato-2xrGBD sensor (mpl-inferno) stimulated with 100 nM rapamycin. The time stamper represents min:s.

Journal of Cell Science • Supplementary information

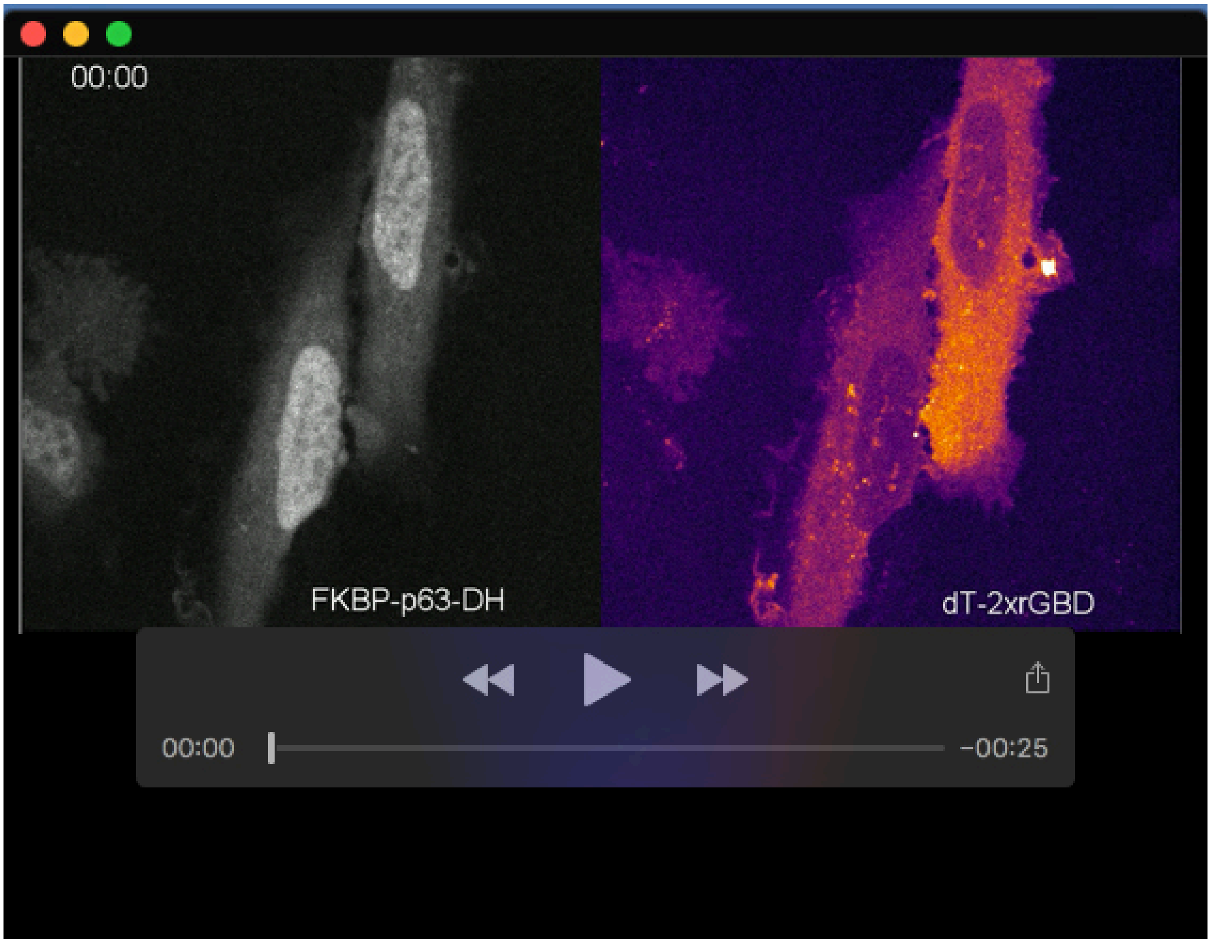

Movie 7. Mitochondria specific activation of Rho detected with the dimericTomato-2xrGBD sensor. Spinning disk time lapse movie of a HeLa cells expressing FRB anchored to the mitochondria (not shown), FKBP-p63-DH (grey) and the dimericTomato-2xrGBD sensor (mpl-inferno) stimulated with 100 nM rapamycin. The time stamper represents min:s.

Journal of Cell Science • Supplementary information

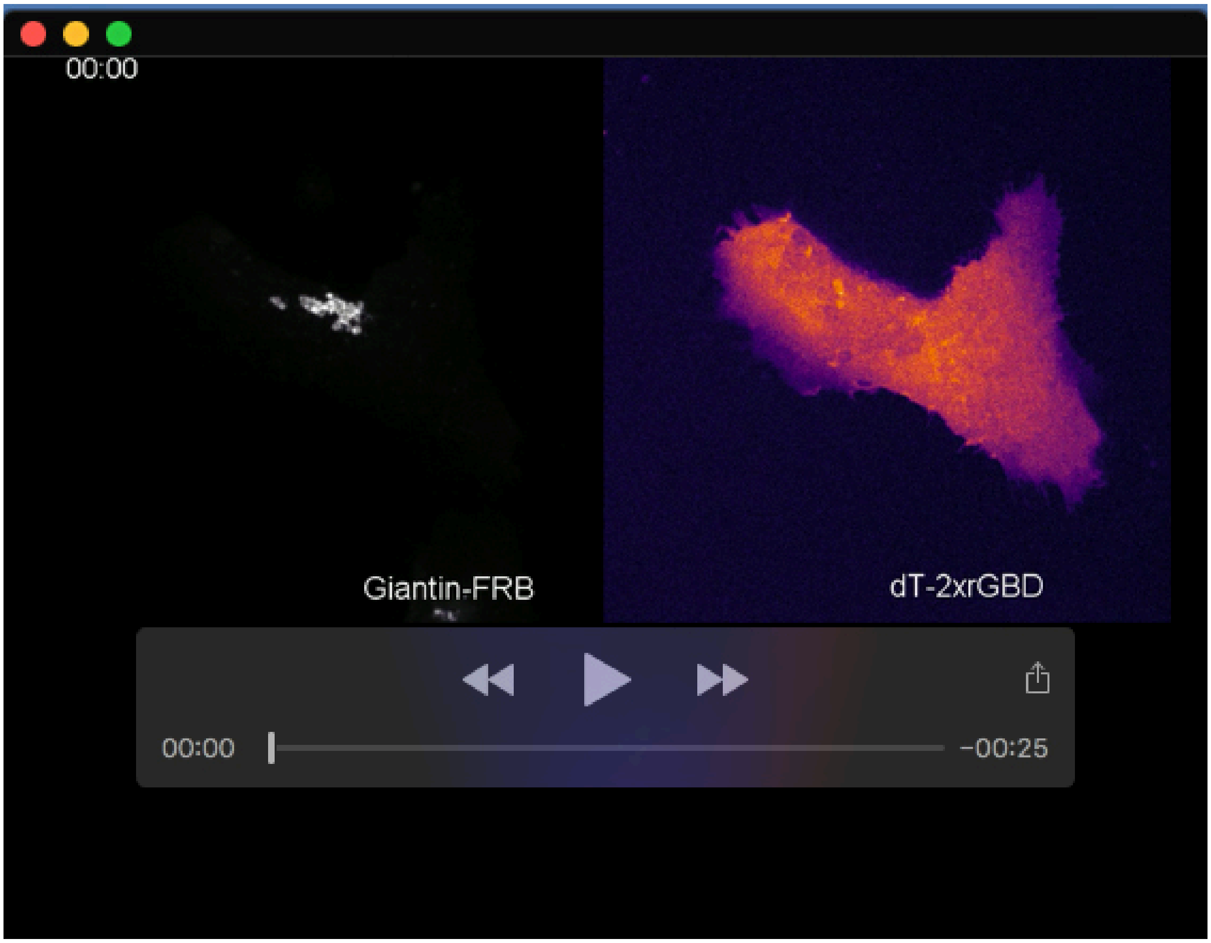

Movie 8. Golgi specific activation of Rho detected with the dimericTomato-2xrGBD sensor. Spinning disk time lapse movie of a HeLa cells expressing FRB anchored to the Golgi (grey), FKBP-p63-DH (not shown) and the dimericTomato-2xrGBD sensor (mpl-inferno) stimulated with 100 nM rapamycin. The time stamper represents min:s.

Journal of Cell Science • Supplementary information

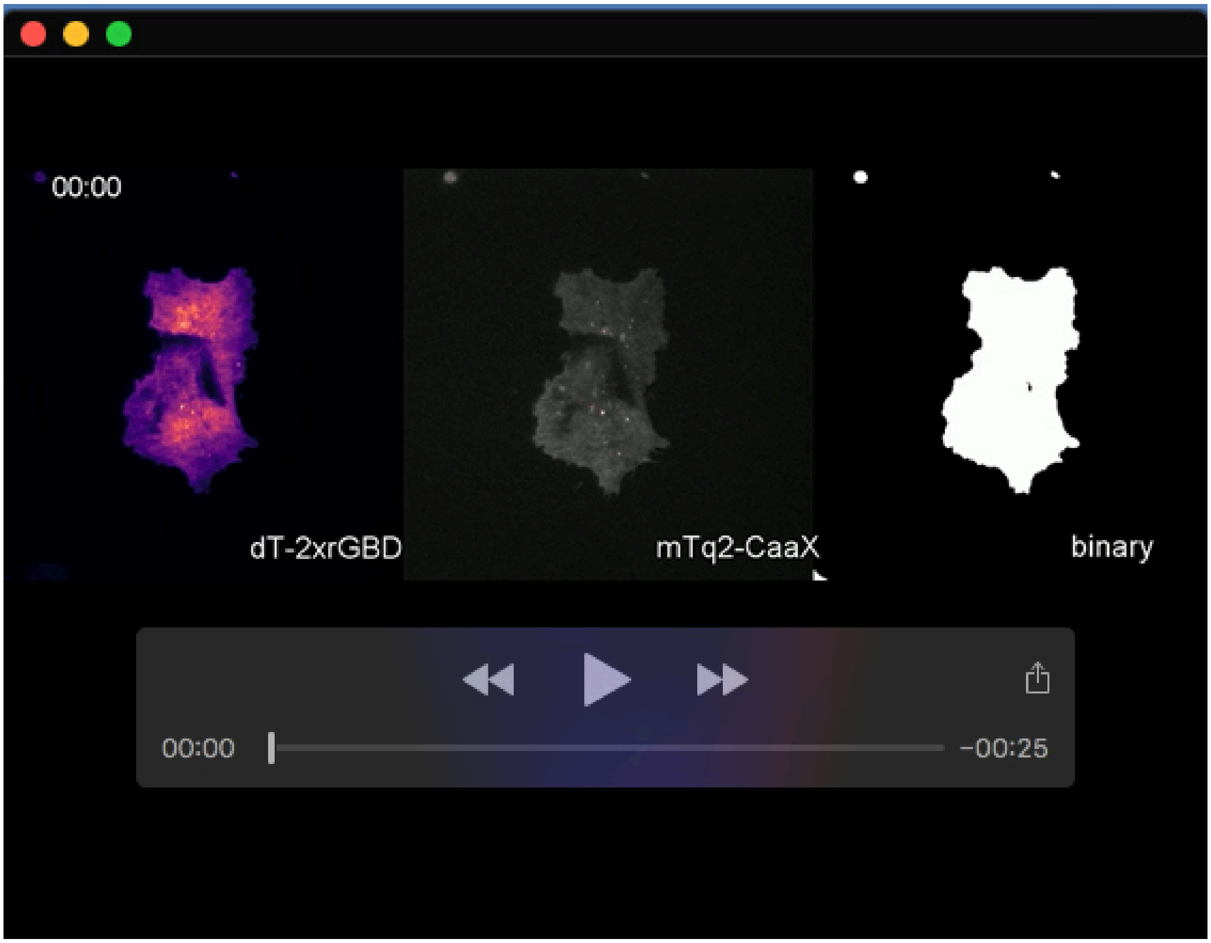

Movie 9. Visualization of active Rho at the basolateral plasma membrane of an endothelial cell. TIRF microscopy time lapse movie of a BOEC stably expressing the dimericTomato-2xrGBD sensor (left, LUT = mpl-magma) and mTq2-CaaX (middle, grey), stimulated with 1 U/ml human α-thrombin. The right shows a binary image representing the cell area. The time stamper represents min:s.

Journal of Cell Science • Supplementary information

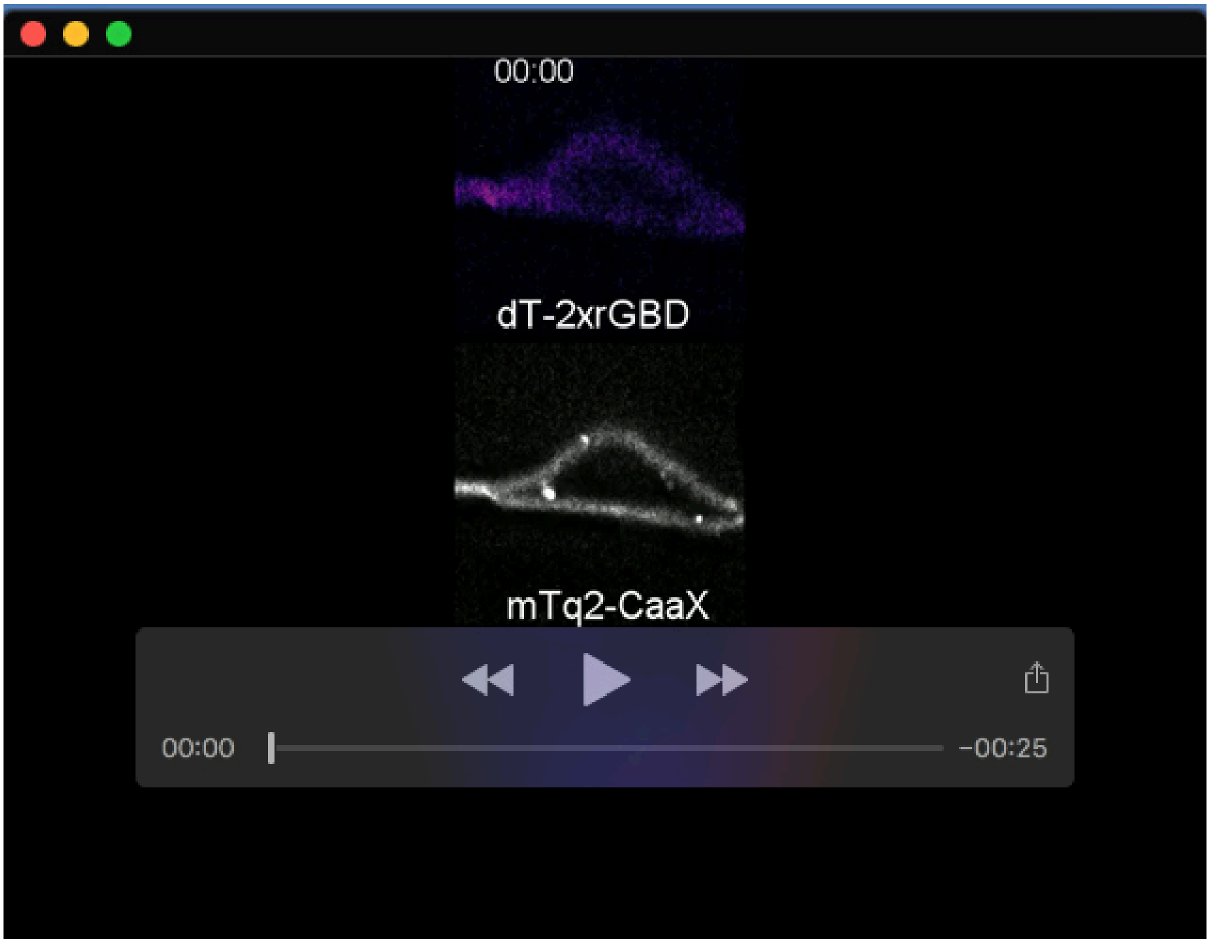

Movie 10. Visualization of active Rho at the plasma membrane in a cross section. Cross section of a lattice light sheet time lapse movie of a HUVEC stably expressing dimericTomato-2xrGBD sensor (upper panel, LUT = mpl-magma) and mTq2-CaaX (lower panel, grey), stimulated with 2,5 µM nocodazole 10 min prior to the imaging.

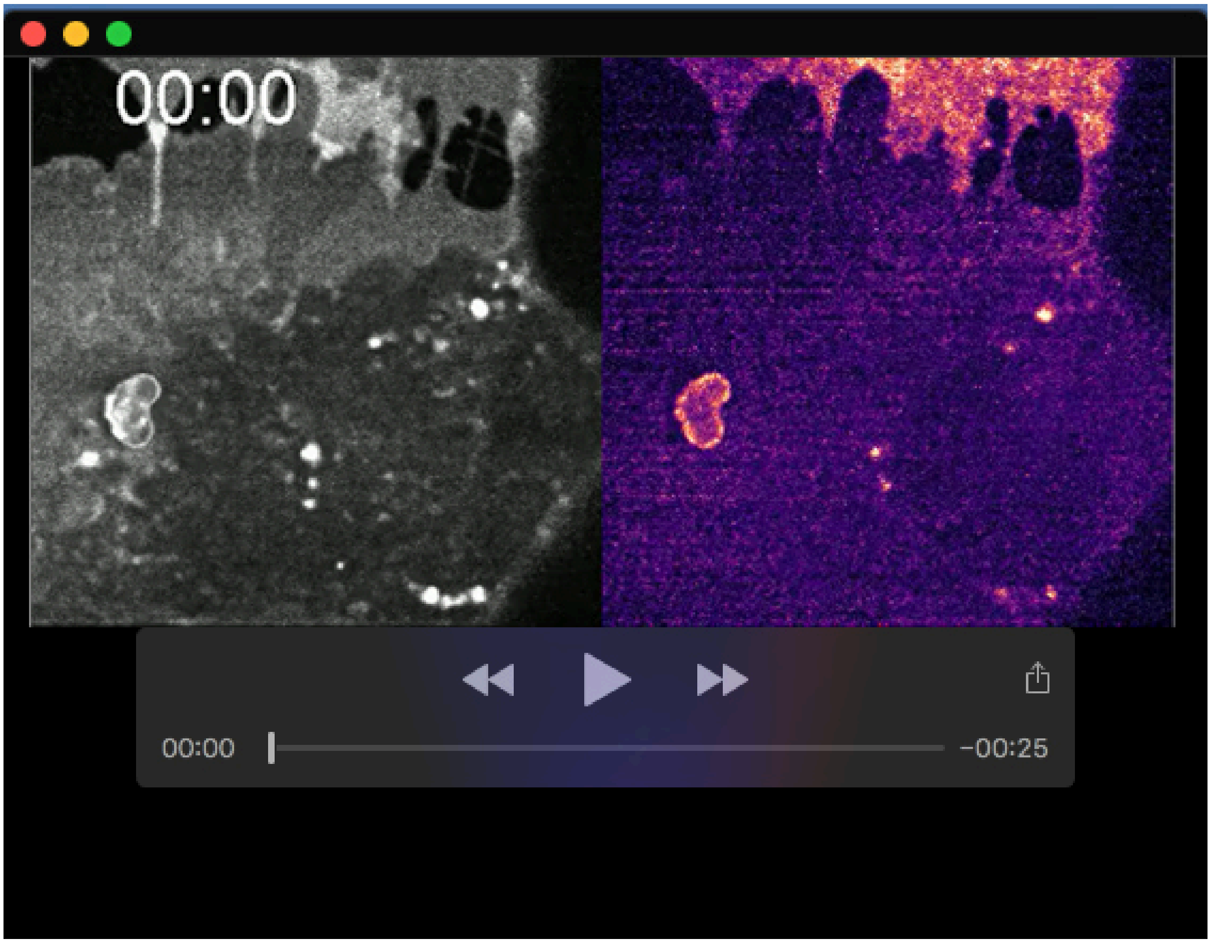

Movie 11. Whole field of view for cross section of Supplemental Movie 10. Maximum Intensity projection for the whole field of view of the cell that the cross section is presented for supplemental movie 10. Lattice light sheet time lapse movie of a HUVEC expressing dimericTomato-2xrGBD sensor (upper panel, LUT = mpl-magma) and mTq2-CaaX (lower panel, grey), stimulated with 2,5 µM nocodazole 10 min prior to the imaging. The cross section was taken vertically at three quarters from the left edge of the image.

Journal of Cell Science • Supplementary information

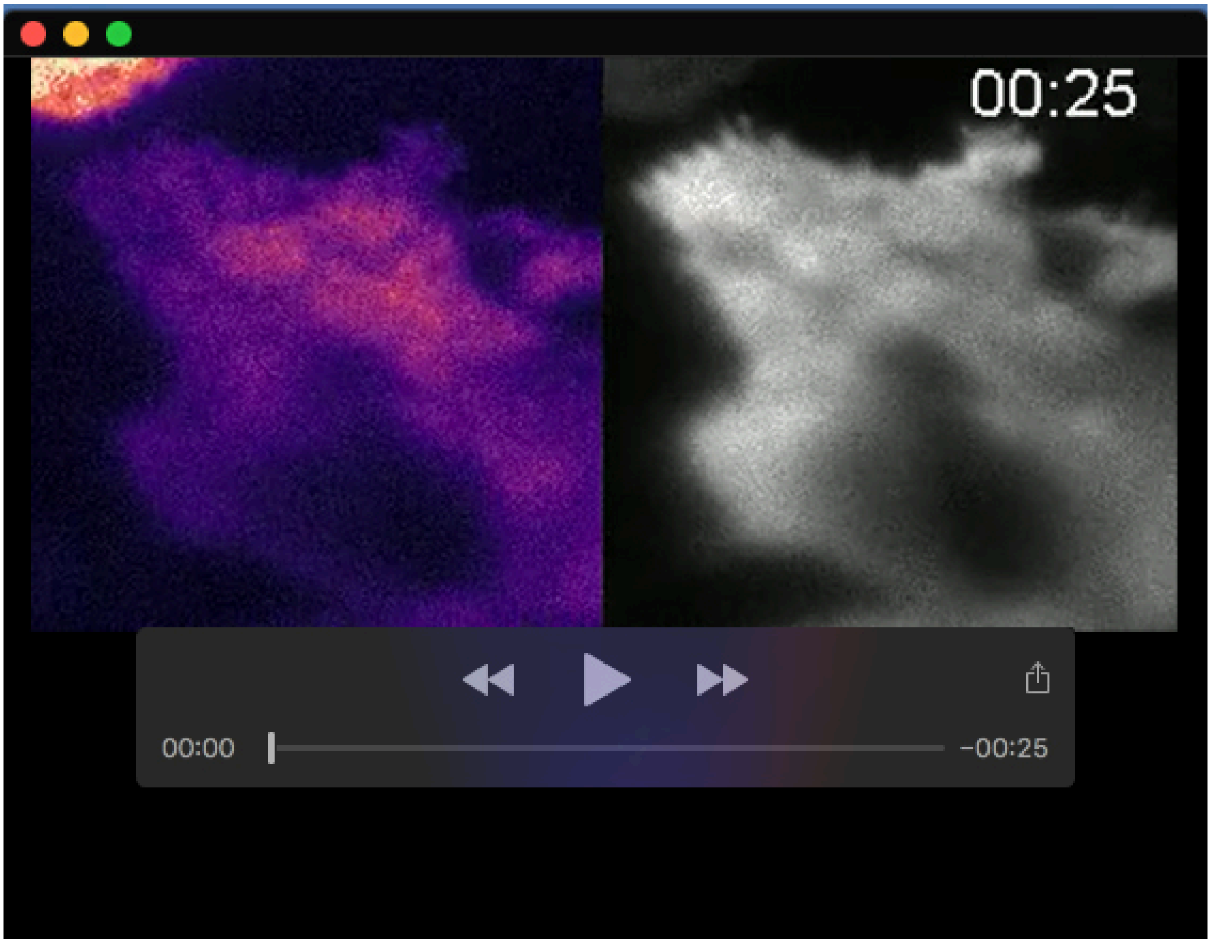

Movie 12. Visualization of active Rho in a contracting endothelial cell. TIRF microscopy time lapse movie of a BOEC stably expressing the dimericTomato-2xrGBD sensor (left, LUT = mpl-magma) and mTq2-CaaX (right, grey) stimulated with 1 U/ml human α-thrombin 5 min prior imaging. The time stamper represents min:s.

Journal of Cell Science • Supplementary information

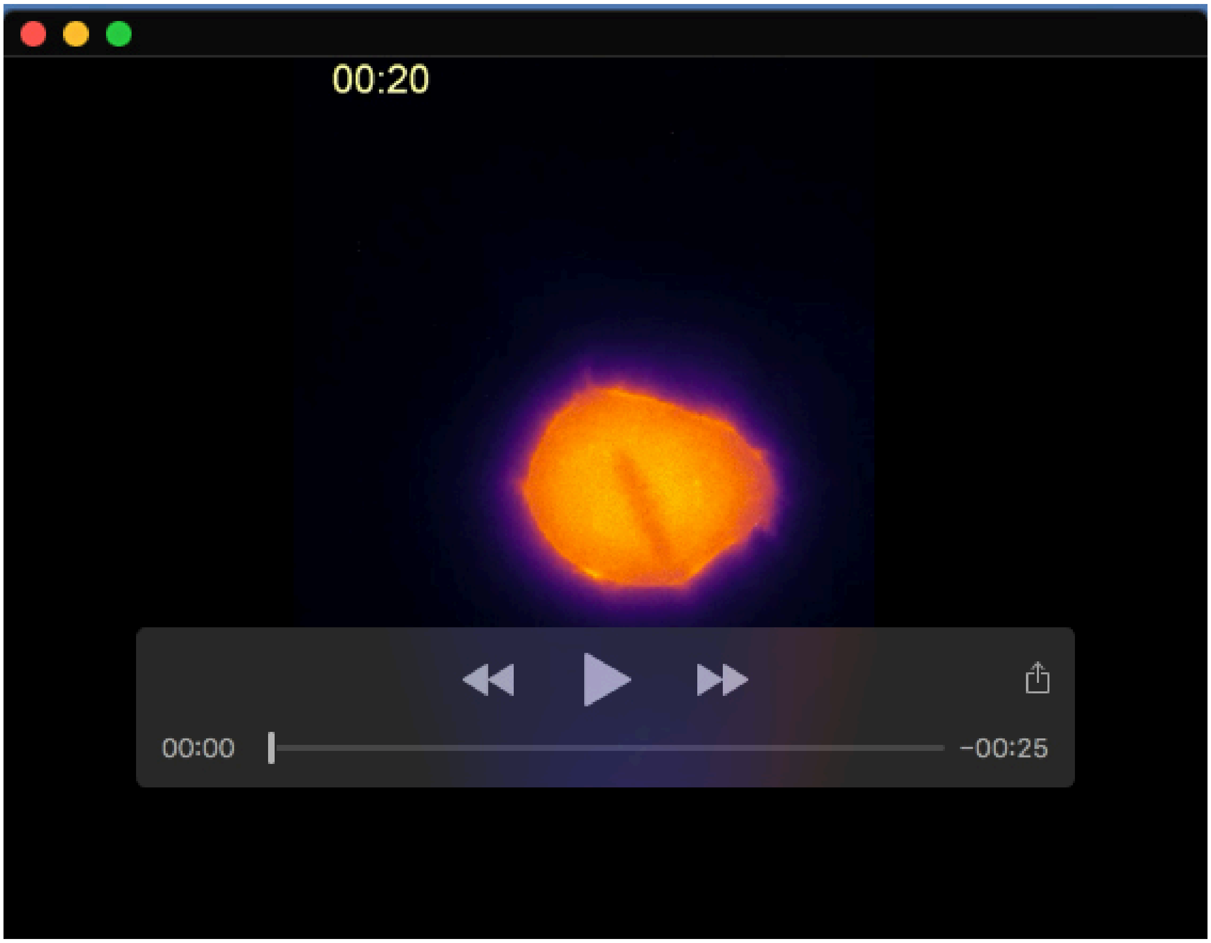

Movie 13. Visualization of active Rho at the cleavage furrow with the dimericTomato-2xrGBD sensor. Wide field time lapse movie of a HeLa cell expressing dimericTomato-2xrGBD. The time stamper represents min:s. LUT = mpl-inferno.

Journal of Cell Science • Supplementary information

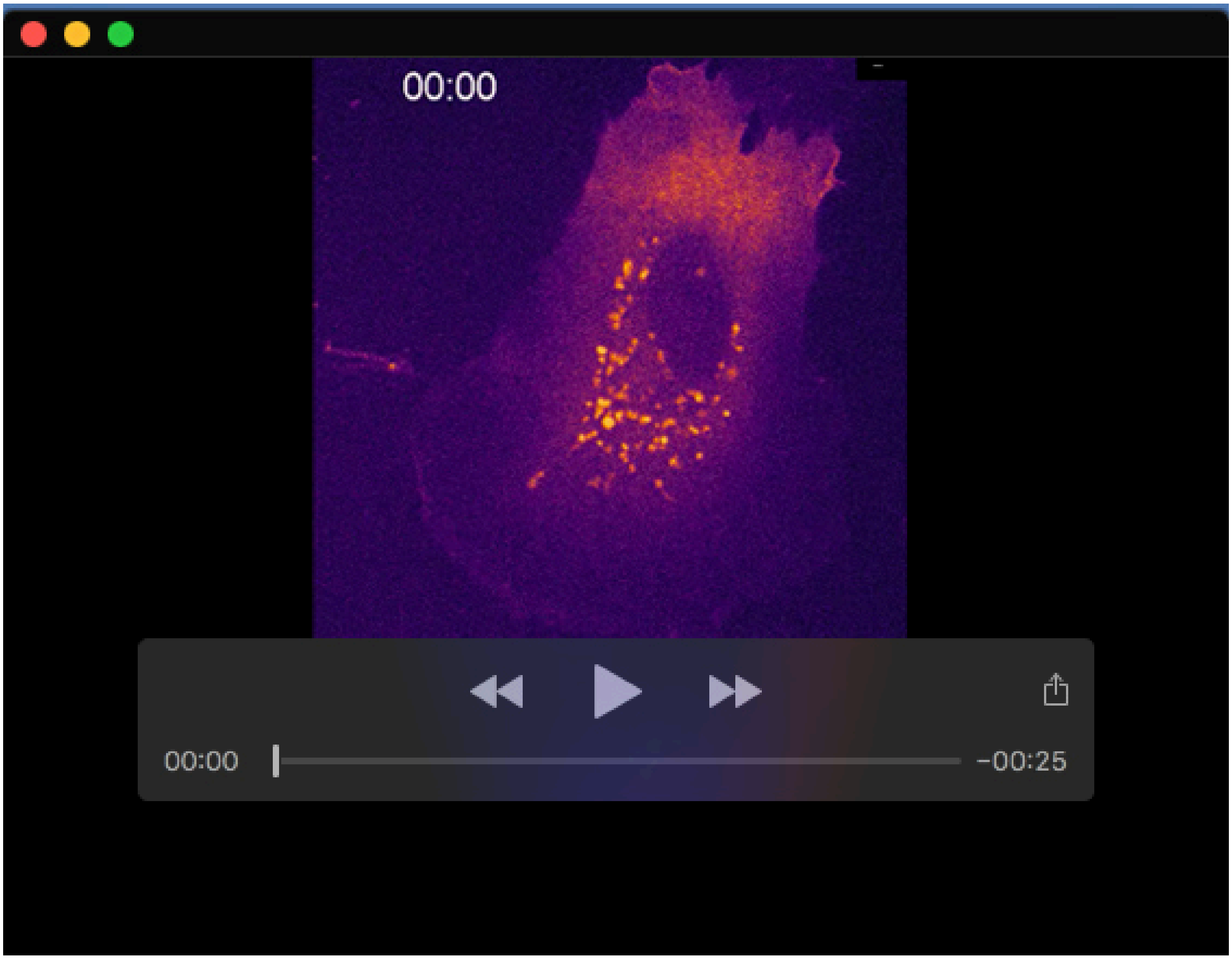

Movie 14. **Visualization of active Rho at the contracting cell edge in a randomly migrating endothelial cell**. Spinning disk time lapse movie of a BOEC expressing the dimericTomato-2xrGBD sensor. The time stamper represents min:s. LUT = mpl-inferno.

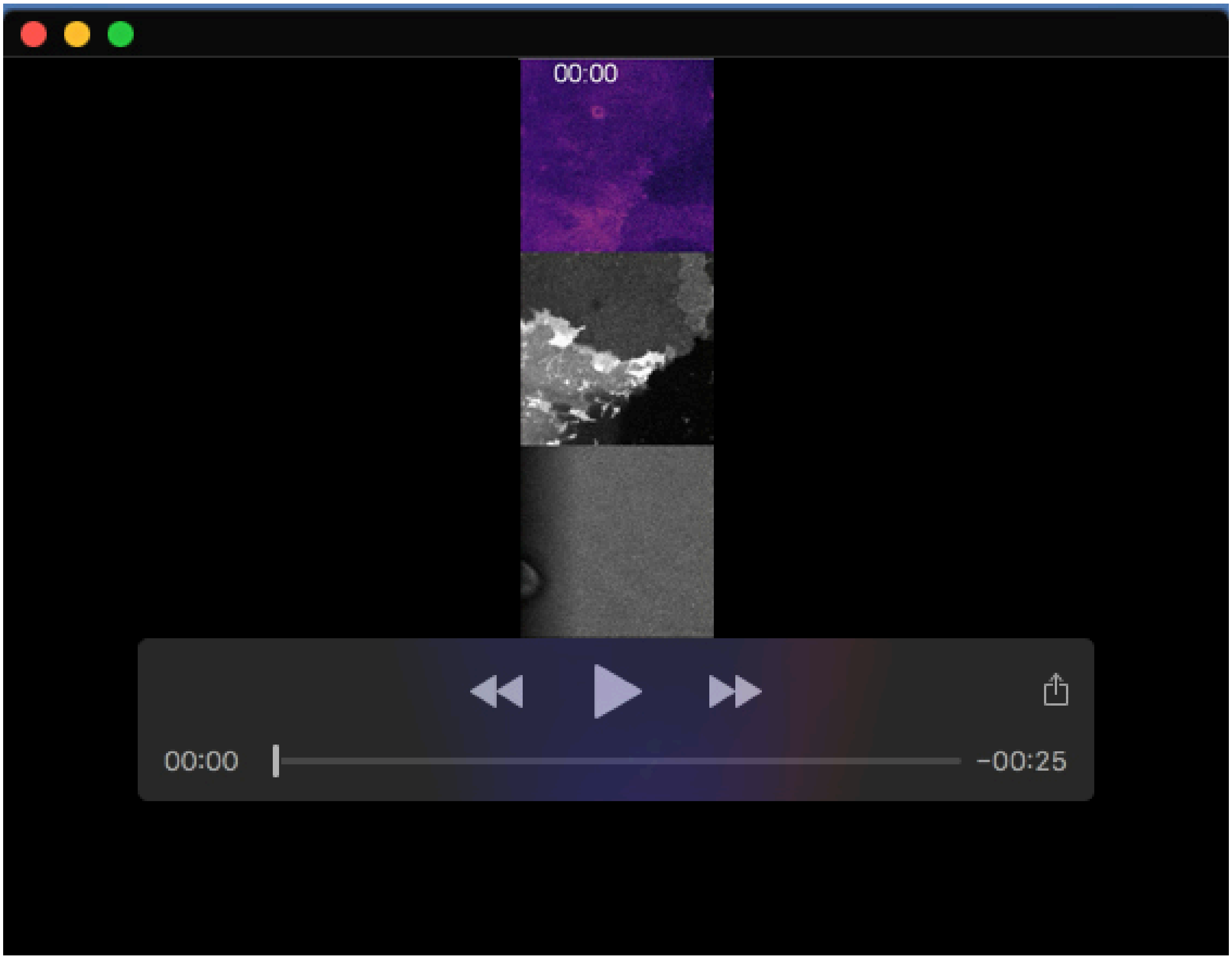

Movie 15. Visualization of active Rho during transendothelial migration. Spinning disk time lapse of a transendothelial migration event with cbBOECs grown in to a monolayer, expressing the dimericTomato-2xrGBD sensor (upper panel) and the membrane marker mTurquoise2-CaaX (middle panel). The transmigrating neutrophil is visible in the transmission panel. The time stamper represents min:s. LUT = mpl-inferno.

Journal of Cell Science • Supplementary information
