# Supplementary material for: Visualizing endogenous Rho activity with an improved localization-based, genetically encoded biosensor
Source: J Cell Sci. 2021 Sep 8;134(17):jcs258823. doi: 10.1242/jcs.258823 (PMC8445605; doi:10.1242/jcs.258823)
Supplement: Reviewer comments [file joces-134-258823_review_history.pdf]

Visualizing endogenous Rho activity with an improved localization-based, genetically encoded biosensor

Elke Mahlandt, Janine J.G. Arts, Werner J. van der Meer, Franka H. van der Linden, Simon Tol, Jaap D. van Buul, Theodorus W. J. Gadella, and Joachim Goedhart
DOI: 10.1242/jcs.258823

Editor: Michael Way

Review timeline

Submission to Review Commons:	10 Feb 2021
Submission to Journal of Cell Science:	21 April 2021
Editorial decision:	26 April 2021
First revision received:	23 July 2021
Accepted:	26 July 2021

Reviewer 1

Evidence, reproducibility and clarity

Major points:

- The affinity analyses need more work. This is against A/B/C isoforms, and also the dimerization affinity between the fluorescent proteins could change the apparent on/off rates. This point is not quantified or discussed. Due to the chemical equilibrium analysis, the apparent equilibrium is not only affected by this on/off rates, but also the local availability (concentrations) of the reacting moieties. In the limit where the biosensor concentration is low within a cellular subcompartment or vice versa, how this is going to change the sensitivity of detection because this can push the reaction in either directions. Since equimolar distribution of the moieties are not guaranteed, this affects the detection characteristics of this biosensor. This point should be discussed and emphasized.
- Fig 1 A: Are the fluorescence changes of the biosensors due to stimulation with histamine completely reversible ? In other words, is it possible to see a total recovery of the signals with pyrilamine or in the presence of another antagonist ? If not, why? Does histamine stimulation induce a maximal activation of RhoA in HeLa cells? What happens in terms of fluorescence changes when the activity of RhoA is inhibited or in the presence of a G?q-inhibitor, and in conditions in which RhoA activating GEF, RhoA GAP or RhoA GDI is overexpressed ? Generally, I think it is useful to have a calibration curve of the biosensors activity, maximal/minimal (ON/OFF) response. For exemple, it would help to answer the question concerning biosensors binding affinity for RhoA ("The function of rhotekin is not clear, it seems to lock RhoA in the GTP bound state (Ito et al., 2018; Reid et al., 1996). We can only speculate that rhotekin has a stronger binding affinity for active RhoA than anillin and PKN1 have." (p.15))
What is the effect of histamine stimulation on a membrane marker expression/location ? What is the effect of histamine stimulation on dT2xrGBD biosensor response when this one is forced to be located in other subcellular compartments (mitochondria, nucleus) by fusing the construct to targeting sequences.
Physiological control: Effect of the presence of the biosensor in cell morphology/behavior... Experimental data concerning this point are evoked in the discussion section. "We demonstrate that low expression of the biosensor, through the truncated CMV promotor, did not inhibit cell division and cell edge retraction. Plus, endothelial cells expressing the sensor still show the typical reaction of contracting followed by spreading, when stimulated with thrombin. Low expression results in a low fluorescent signal of the sensor." (p.16) I think this results would deserve a section in this manuscript.

© 2021. Published by The Company of Biologists under the terms of the Creative Commons Attribution License (https://creativecommons.org/licenses/by/4.0/).

- Fig 2D : "The anillin sensor AHD+PH showed a 15% decrease in cytosolic intensity (Figure 2D), but it also relocalizes to striking punctuate structures upon histamine stimulation. These structures did not seem to represent local, high activity of RhoA, as the optimized rGBD sensor in the same cell showed no such locally clustered RhoA activation, but rather a homogenous activation at the membrane and a 60% drop in cytosolic intensity. Similar punctuate structures were observed in endothelial cells, when stimulated with the strong RhoA activator thrombin (Supplemental Movie 5)." And p. 15 : "However, we noticed that the AHD+PH sensor, containing aGBD, C2 and PH domain, localizes in a punctate manner. These 'dots' were observed in both HeLa cells and endothelial cells and were only observed with the AHD+PH RhoA sensor. As aGBD does not localize in puncta, it seems that the localization is caused by domains other than of the RhoA binding domain, i.e. the C2- and/or PH-domain." Punctate structures are also present in HeLa cells expressing the anillin sensor before histamine stimulation (see Supplemental Movie 4). Moreover, punctuate pattern activated by thrombin in endothelial cells looks different (more widespread) than the one activated by histamine in HeLA cells. In addition, these structures can also be found in human endothelial cells expressing dT2xrGBD (fig. 6B, Supplemental movie 10). What are those structures thrombin activated in endothelial cells that would be similar to the ones in Hela cells activated by histamine and that "did not seem to represent local, high activity of RhoA"? This is not further commented by the authors.

- Fig 3A: "The rGBD sensors solely colocalized in the nucleus with RhoA but not with Rac1 and Cdc42, indicating that rGBD specifically binds constitutively active RhoA." What about dT2xrGBD binding specificity for the three homologues RhoA, RhoB and RhoC? This point is evoked in the discussion part (p.16) but there is no experimental data to support it "The specificity of the relocation sensor is determined by the binding specificity of the GBD. The rGBD binds the three homologues RhoA, B and C but not to Rac1 and Cdc42". So, why rGBD is presented as a RhoA biosensor?

- Fig 3B: The data scatter for the dTomato-2xrGBD is very wide compared to the mScarlet- 1xrGBD. What is causing this wide data scatter and such heterogeneous response? This is a problem if the sensor is really so heterogeneously responding to a strong mutant of RhoA, is this a dimerization-dependent problem?

- These domain-based biosensors could cause dominant negative/inhibitory artefacts. Also the dimerizing fluorescent proteins could introduce oligomerization of the signaling complex which is not real in cells and clearly affect phenotype. These issues should be tested and addressed by a quantitative measure of cell behavior against increasing concentration/changing dimerization potentials of the biosensor in live cell assays.

- Fig 4 C: "Given the successful improvement of the rGBD-based biosensor by increasing the number of binding domains, we explored whether the same strategy can be applied to the G protein binding domains from PKN1 and Anillin" and "The dimericTomato-2xrGBD sensor shows the best relocation efficiency, with a median change in cytosolic intensity of close to 50%"... So why the dT-2xaGBD construct has not been tried ?

- p.9 : "None of the pGBD sensors showed a clear membrane localization upon stimulation with histamine (Figure 4A). The increase in cytosolic intensity observed in some cells, seems to be caused by changes in cell shape." Do changes in HeLa cell shape induced by histamine stimulation? How this can be explained? Do some cells expressing the rGBD sensors (single, tandem and triple and dimericTomato) undergo these changes of shape too, upon histamine stimulation? If yes, to what extent these changes in cell shape affect signals?

- p9: Overall, the paragraph about Fig 4 E,F is not clear. What amino acid sequences of G Protein Binding Domains of Anillin and PKN1 bring for the understanding of rGbD, aGBD and pGBD sensors?

- p. 12, Fig 6C, Fig. 6E: "The membrane marker showed a relatively small increase in intensity after stimulation and the curve did not show the same pattern as the RhoA biosensor intensity curve. Therefore, we conclude that the increase in RhoA biosensor intensity is caused by relocalization." It surprises me that decrease in cell areas induced a very small increase in fluorescence intensity of the membrane marker. It would be very helpful to see a figure with a quantification of the

© 2021. Published by The Company of Biologists under the terms of the Creative Commons Attribution License (https://creativecommons.org/licenses/by/4.0/).

membrane marker intensity changes during this process. What about a cytoplasmic marker? In addition, how does the movement artefact is corrected?
"Our data revealed that the RhoA biosensor displays RhoA activity at subcellular locations where RhoA activity is expected, and appears mostly independent of fluorescent intensity measured by a separate membrane marker." This part should be developed further. Are there examples of cells for which the biosensor activity is dependent on fluorescent intensity measured by a separate membrane marker?

- Discussion (p.16): "Comparing relocation sensors to FRET sensors, both have their own advantages and disadvantages." The dT2xrGBD sensor is here presented as a new relocation sensor for RhoA activity. However in general, there should be more development of the direct comparisons, pros and cons, with quantitative data or more details allowing to have a general overview of the advantages and disadvantages of this new relocation biosensor as compared to the existing ones.

Minor points:
- Overall, scale bars should have to be included in HeLa cells microscopy images.
- It was not clear until the Methods section that the widefield analysis appeared to be normalized against another fluorescent protein-based cytoplasmic signal to correct for variations in cell volume. I think this point should be mentioned in the main text more prominently and emphasized so that readers are not misled.
- p. 9 : "Anillin AH+PH sensor" instead of "Anillin AHD+PH sensor"
- Fig 2B and 2D : Explain what parameter is used for the normalization of each signals ?
- Fig. 1A, top panel: it would be good to know which images correspond to the addition of histamine and which ones correspond to the addition of pyrilamine
- "TRIF microscopy" is written in legends of Fig. 6 and of Supplemental movie 11, and in Materiel and Methods section p. 23
- Fig. 3 legend: Correct "mScralet-I-1xrGBD"
- Fig 4F, legend: " Anillin and the bound RhoA are depicted in dark and light yellow, respectively. PKN1 and the bound RhoA are depicted in light and dark blue, respectively." Color codes in legend are opposites to the figure ones.
- p.11 : "To examine this, we used a rapamycin-induced hetero dimerization system to recruit the dbl homology (DH) domain, of the RhoA activating GEF p63, to the membrane of the Golgi apparatus." Corresponding references should be included.
- Fig. 5A : Explain FRB, Fig 5C : no unit for a ratio

Significance

Mahlandt et al. optimized and compared several G protein binding domain (GBD)-based biosensors in order to improve the potential of existing RhoA-domain-based biosensors for visualizing and reporting RhoA subcellular activity in living cells and tissue. The authors demonstrate that fusing a dimerizing fluorescent protein to the rhotekin GBD (rGBD) is an efficient strategy to increase the brightness of the sensor. The use of Rhotekin-RBD as affinity domain for Rho-class of GTPase is very well established, both in the methods of affinity pulldowns and in biosensor designs for Rho-class of GTPases in the field. The authors show that the dimericTomato-2xrGBD biosensor can indicate endogenous RhoGTPase spatial activity in dividing HeLa cells and during cell retraction of human endothelial cells.

The dimericTomato-2xrGBD biosensor is thus introduced and described as a RhoA localization-based biosensor, however no experimental data demonstrate the binding specificity of the biosensor for RhoA. Moreover, authors discuss about a previous work showing that rGBD binds the three paralogs RhoA, RhoB and RhoC. This point and the apparent singular claim of this biosensor reporting RhoA activity as this manuscript alludes to are inappropriate and misleading. This point especially in light of the field has moved on in the past 20 years to assign more specificity (not less) to which GTPase the biosensors are being specific, i.e., via FRET, etc., significantly tempers the enthusiasm of this reviewer. In addition to this main issue, the incomplete characterization of the relative affinities of the domain to the target GTPase isoforms and of the dimerization affinities of the fluorescent proteins (which could change the apparent reaction rate constants), and the impact of which on the reversibility, oligomerization states and detection sensitivity, and the biology, also appeared lacking. Additional stoichiometric considerations and apparent reaction equilibrium that are

© 2021. Published by The Company of Biologists under the terms of the Creative Commons Attribution License (https://creativecommons.org/licenses/by/4.0/).

impacted by the relative concentrations of interacting moieties require careful and further analyses, study and discussion.
In general, I think that this work could be interesting to a more specialized field audience with further analyses of the affinities of the interacting moieties and better characterization of the behavior of this biosensor in living cells since it is likely causing oligomerization of the signaling units due to the forced dimerization of the detection unit.

Referees cross-commenting

This is a dimerizing probe. It gets pretty bulky. Is dimerization occurring prior to GTPase binding or after? Is the dimerized probe/GTPase complex somehow more stable than would otherwise be if they were monomeric? If so, how would that affect the lifetime of the detection and also the diffusivity of the probe("s", if already dimerized) and possibly the whole oligomer?

It still feels to me that, yes new brighter fluorescent proteins were used, and dimerization andmultimerization of the signaling complex increased the SNR of the system, but the whole premise just reverted the biosensor field back 20yrs, which has been my biggest single concern regarding this paper.

Reviewer 2

Evidence, reproducibility and clarity

Summary:

Visualization of subcellular activity of GTPases is critical for the understanding of signal transduction of cell growth, differentiation, morphogenesis, etc. For this purpose, researchers often use relocation probes, which comprise a fluorescent protein(s) and a GTPase-binding domain(s), and move from cytosol to the location of active GTPases. The authors improved a previously reported RhoA probe with a strategy of increasing the avidity of RhoA-binding domain and optimizing the fluorescent protein. In the beginning, the authors declare "the relocation of the original, single rGBD monomeric fluorescent protein sensor is hardly detectable" in HeLa cells. To overcome this problem, they developed six constructs by changing the number of rGBD (rhotekin GBD) domains and fluorescent proteins. They found that the increase in the number of rGBD and a dimeric prone fluorescent protein, tdTomato, generate a better probe for RhoA activity. The specificity was examined by using active Rac1 and Cdc42 proteins. Different RhoA-bind domains derived from Rhotekin, PKN1, and Anillin were compared to show the superiority of rhotekin GBD. Finally, they show that subcellular RhoA activation detected by the probe is consistent with the knowledge on RhoA activation by using vascular endothelial cells. Overall this work has been well done in an organized way and disclose a novel RhoA probe that will be useful in future research of RhoA.

Major comments:
1. Reproducibility: The number of analyzed cells is described in the legend, but the number of independent experiments is not shown. This is critical to evaluate the reproducibility of the data. Preferably, the data should be presented to show data set derived from each trial clearly. It should also be described how cells were selected for the analysis? It is also preferable to apply automatic analysis. Ideally, the raw data with code sets for analysis should be presented.

2. A serious defect of the relocation probe is the dependency on the expression level. The lower the number of the probe in a cell, the higher the fraction of recruited to active RhoA. However, lowering the probe concentration will be accompanied by dim fluorescence. The authors should describe how the optimal expression level was achieved.

3. Statistical analysis is absent throughout the paper.

© 2021. Published by The Company of Biologists under the terms of the Creative Commons Attribution License (https://creativecommons.org/licenses/by/4.0/).

Minor comments:
1. In Figure 1, mNeonGreen (mNG) was used as the fluorescent protein fused to rGBD instead of EGFP, which was used in the original paper. For a fair comparison with the previous report, analysis using the original probe, i.e., EGFP-rGBD, is desirable. Or, the author may simply tone done.
2. In the introduction, it says " The RhoA FRET sensors achieve subcellular resolution to a certain extent, but due to their design they do not localize as endogenous RhoA". Reference is required.
3. rGBD should be rhotekin GBD. It should be clearly stated in the beginning.
4. The reason why the CMVdel promoter is used should be stated clearly.
5. Page 23: TRIF should read as TIRF.
6. Figures: Grey letters should be avoided.
7. Fig. 3A: Apparently the probe binds to Rac1 G12V to some extent. The discrepancy of RhoA localization between mSca-1xrGBD and dt-2xrGBD must be discussed. This observation clearly suggests that GBD may change the localization of RhoA. It is interesting to note that Rac1 and RhoA may localize to the nucleolus.

Significance

1. This work discloses an improved RhoA probe, which will be welcome by the researchers in the field of small GTPases.
2. Novelty of increased GBD: The idea of increasing the GTPase-binding domain in the relocation probe was reported some time ago: Augsten et al., Live-cell imaging of endogenous Ras-GTP illustrates predominant Ras activation at the plasma membrane. EMBO Rep. 7, 46-51 (2006).
3. Novelty of rhotekin GBD: The reason why GBD of PKN is chosen in intramolecular FRET biosensors such as DORA and Raichu is that the affinity of other GBD's is too high [Table 1, Yoshizaki et al., J. Cell Biol. 162, 223-232 (2003)]. Judging from this old data, GBD's of mDia and Rhophilin, may work better than that of Rhotekin. Moreover, it is known that PH domain may be required for proper conformation of GBD's. Thus, it is not surprising that removal of PH domain from the Anillin probe abolishes its translocation ability. Therefore, to the reviewer's eyes, the choice of GBD in Figure 4 is biased to those that will work less efficiently.
4. Authors' proposal of "systematic optimization" sounds exaggerated, considering the small number of constructs tested in Fig. 1 and Fig. 4. Similarly, it is not clear whether dimerize prone-fluorescent proteins are better choice by simply comparing tdTomato and mNeonGreen.
5. Keywords of expertise: Fluorescent probes. Cell signaling.

Referess cross-commenting

Because Review Commons does not specify the journal to be published, the request by the Reviewer #1 sounds too much. The probe reported in this work deserves publishing, although it may not be a ground-breaking probe.

Reading the comments by the other reviewers, following concerns should be cleared.

1. Relationship between the probe's concentration and the response.

2. Specificity to RhoA, RhoB, and RhoC

3. The effect of the cell morphology as pointed by Reviewer #1.

To Reviewer #1
-Since equimolar distribution of the moieties are not guaranteed, this affects the detection characteristics of this biosensor. This point should be discussed and emphasized The probe will diffuse rapidly within cytosol. Therefore, subcellular concentration of the probe may not affect significantly on the performance of the probe.

-What is the effect of histamine stimulation on dT2xrGBD biosensor response when this one is forced to be located in other subcellular compartments (mitochondria, nucleus) by fusing the construct to targeting sequences.

I did not understand this question quite well.

© 2021. Published by The Company of Biologists under the terms of the Creative Commons Attribution License (https://creativecommons.org/licenses/by/4.0/).

Reviewer 3

Evidence, reproducibility and clarity

Summary

In this paper, Mahlandt et al compared and improved relocation sensors to visualize the activity of endogenous Rho. As a result of screening for several Rho binding domains (GBDs) and the number of repeats, the authors found that dTomato-2xrGBD is optimal, and succeeded in visualizing the activity of Rho during cytokinesis and migrating cells. Overall, this sensor would be a useful tool for many cell biologists. The data are represented clearly in the figures. I provide some concerns; that would be worth addressing in a revised version.

Major comments

1. The authors should experimentally show the quantitative relationship between biosensor expression level and degree of relocation. In principle, this relocation type sensor binds to the endogenous GTP-bound Rho. Since the number of endogenous GTP-bound Rho is limited in cells, the degree of relocation is considered to be dependent on the expression level of the sensor. If the number of biosensors expressed is too small in a cell, the response will be saturated. If the number of biosensors is too large, the relocation will be weakened and the Rho signal will be suppressed. Furthermore, although a weak promoter is used, the heterogeneity of the expression level in each cell makes quantitative analysis difficult, especially in transient expression experiments. I would like to suggest the addition of quantitative experimental data.
2. Most of the time-series data show only a representative example, namely, N = 1. In relation to the aforementioned issue, data and distribution derived from several cells (e.g. SD) should be shown in a clear manner.

Minor comments

3. I hesitate to call the biosensor developed in this study "RhoA sensor". This is because, as the authors mention, it has been reported that the rGBD also binds to RhoB and RhoC. If the authors call it a RhoA sensor, they should investigate the specificity of binding to RhoB and RhoC in addition to RhoA. If not, I would like to suggest changing the name to "Rho sensor" instead of "RhoA sensor".

Significance

Rho is one of the low molecular weight G proteins, which regulate the reorganization of the actin cytoskeleton. As biosensors for visualizing the activity of Rho proteins, it has been reported intramolecular and intermolecular FRET biosensors and relocation sensors. The latter is less widely used than the former, because of insufficient sensitivity and specificity. Therefore, the improvement of Rho biosensors is really important and needed in the community of cell biology research field. The importance of this manuscript, I believe, is that the authors compared the existing relocation type Rho sensors. This is informative.

Rho is one of the low molecular weight G proteins that regulate the rearrangement of the actin cytoskeleton. Intramolecular and intermolecular FRET biosensors and relocation sensors have been reported as biosensors for visualizing the activity of Rho proteins. The latter is not as widely used as the former due to its inadequate sensitivity and specificity. Therefore, improving the Rho biosensor is very important and is needed by the community in the field of cell biology research. I believe the importance of this manuscript is that the author compared existing relocation-type Rho sensors. This is beneficial and informative.

My expertise: Cell biology, live-cell imaging, development of genetically encoded fluorescent probes

Referees cross-commenting

© 2021. Published by The Company of Biologists under the terms of the Creative Commons Attribution License (https://creativecommons.org/licenses/by/4.0/).

I generally agree with Reviewer 2's opinion. The opinions of our three reviewers can be summarized in three points: expression level, specificity, and statistical analysis and representation. I think these should be asked to the authors as major critics that should be addressed before publication.

Reviewer 4

Evidence, reproducibility and clarity

SUMMARY:

Mahlandt and colleagues use advanced microscopy techniques to test new configurations of several Rho relocation sensors, which report on the activity of members of the endogenous RhoA GTPase family of proteins. A novel variant containing the dimericTomato fluorescent protein and a double rGBD domain shows a substantial increase in dynamic range in comparison with 2 originally published sensors and other new variants they tested. They use a cellular assay to show that this novel variant is specific for the activity of Rho family of Rho GTPases and not the Cdc42/Rac families. Finally, the authors show that this new variant can be used to measure a specific localised increase of Rho activity at the Golgi, and during cell division and cellular morphology changes that are known to activate the RhoA family of Rho GTPases. The biosensor can be useful for the community. However, I think the paper is not well written (I was very confused by several statements). The manuscript should be thoroughly proofread, there are quite some unclear or duplicate passages (for examples, see "text comments" below). Currently this hampers the interpretation of the manuscript for the reader. The authors are very dogmatic - they make claims about the literature that I do not agree with at all. Some of these unbalanced views will confuse the non-expert readers.

MAJOR COMMENTS:

-The reported dTomato-rGBD sensor is unable to distinguish between the different members of the RhoA familiy of Rho GTPases (measures combined activity of RhoA, RhoB and RhoC), which is unclear for the reader in the current text phrasing in the introduction. The authors seemingly suggest throughout the manuscript to work with a specific RhoA biosensor, which is not the case. This strong statement is completely misleading. The authors need to refer to the biosensor being specific for Rho (RhoA,B,C) GTPases versus Rac1/Cdc42 biosensors, and discuss what this means for the field. Some discussions about this are made in a JCB paper by Graessl et al, that the authors also cite.

-If the authors really want to sell that the biosensor is only specific for RhoA, then they need to make a series of experiments with RhoB and RhoC dominant positive/negative constructs, to tackle that specific point.

-Did the authors consider to use the artificial GBD from Keller, 2019 to make a specific relocation sensor for RhoA? Perhaps the authors can comment on the feasibility of this approach?

-A strong (dogmatic) statement is that Rho GTPases FRET sensors report solely on the activity of GEFs. This is not the case, these sensors report on the flux of GAP and GEF activity for Rho GTPase in cells. This is also true for relocation sensors, and has been documented in work from the Bement/Pertz/Nalbant/Dehmelt labs.

-From the data in Figure 1, it seems to follow that the efficiency of PM relocation is mainly determined by the number of rGBD modules on the sensors. Could the authors speculate on how this works in practice; is the multi-rGBD sensor increasingly kinetically trapped by a single RhoA molecule, or is the sensor mostly bound to multiple RhoA molecules at the PM?

-Some form of statistical analysis should be performed on the data to give the reader a sense of robustness of the findings and its uncertainty. Either a non-parametric test on the median, confidence intervals or e.g. boxplots showing notches.

© 2021. Published by The Company of Biologists under the terms of the Creative Commons Attribution License (https://creativecommons.org/licenses/by/4.0/).

-Time-series now show single example traces (fig1C, fig2B,D, fig5B). It would be informative for the reader if the curves of all experiments were plotted, and statistical analysis would be performed on the data. It is unclear how representable the kinetics in these curves are.

-About the spatial patterns of Rho activity (cytokinesis, tail retraction, ...), the reviewers agree that statistical analysis is much more difficult. But maybe showing 2-3 cells instead of only one, would make the data more convincing.

MINOR COMMENTS:

-(fig4a) dTomato-2xpGBD, why is this not good? how is it possible that it binds good to nucleus, but no translocation is observed? const activity? expression levels?

-(fig4f) The aGBD/pGBD binding sites for RhoA show great overlap but bind to completely different sites at RhoA, is this correct? (color scheme used for the structures is not easily interpretable)

-(fig5) Unclear how the intensity at the specific organelles is measured? were the organelles segmented or hand-drawn ROI based? The quantified difference is very small, no statistics are performed, and it is unclear how it was measured. This is currently weak evidence for the main claim in this subsection.

-(fig5) The kinetics of the response to histamine (fig1C) seems to be much faster as the rapamycin mediated increase in fig5B for the PM condition. Any explanation for this? Why does it not reach a plateau like in the histamine experiments?

-(fig6F) Data from 6D is repeated here, 6F could potentially show aggregate time-series instead of individual cells. Would also improve interpretation if the membrane marker curve is plotted in every subfigure. Potentially membrane marker intensity could be used to normalise the (TIRF) measurements?

-can the authors provide scale bars on the micrographs, as is usually done in any manuscript ? It would also be useful to put time labels when images corresponding to timeseries are shown.

-ratio values are dimensionless by definition, so no need to write "arbitrary units"

TEXT COMMENTS:

-(abstract): "Due to the improved avidity of the new biosensors for RhoA activity, cellular processes regulated by RhoA can be better understood." -> unclear what the authors mean with 'avidity' in this context? (here, and throughout rest the manuscript)

-(introduction) "Although these three Rho GTPases may have different functions, we generally refer to RhoA in this manuscript." -> unclear what message the authors try to convey with this sentence.

-(introduction) "Active RhoA mainly localizes at the plasma membrane, due to its prenylated C-terminus" -> where else would it be localised? Where is inactive RhoA localised?

-(introduction) "Unimolecular Rho GTPase FRET-based biosensors consist of the Rho GTPase itself, a GBD and a FRET pair." -> a short description/explanation of what a "FRET pair" is would benefit the non-specialised audience.

-(Results p9) "For the original Anillin AH+PH sensor...around 15%" -> did the authors do the experiment with G14V on this original sensor variant?

-(Results p9) The "mScarlet-I-AHD+PH" seems to perform quite good on the fig4D assay, but is not present in 4C analysis?

-(Results p9) "mScarlet-I-AHD+PH" is the same as "AHD+PH (aGBD+C2+PH)"? descriptions unclear. Would generally advise to thoroughly check the manuscript for consistency of condition descriptions / abbreviations in both text and legends.

© 2021. Published by The Company of Biologists under the terms of the Creative Commons Attribution License (https://creativecommons.org/licenses/by/4.0/).

-(Results p12) "Visualizing endogenous RhoA activity" as subsection title could potentially confuse readers, since all measured Rho activity in the manuscript is endogenous.

minor text:

-(fig3b legend) "mScralet-I-1xrGBD"

-(fig6H legend) "TRIF", and "cbBOEC" is same as "BOEC"?

Significance

The novel "Rho" family GTPase relocation sensor that the authors present might be a significant improvement over the currently existing ones (for refs, see manuscript). This might provide a substantial technical advance in the field and increases the utilisation and the reproducibility of this tool in the field. This sensor will be of significant interest for the Rho GTPase signalling field, and more broader the cytoskeleton biology community. My expertise in Rho GTPase biology, biosensor development and advanced microscopy granted me the opportunity to judge the complete manuscript

---

Author response to reviewers' comments

Reviewer #1

Major points:

- The affinity analyses need more work. This is against A/B/C isoforms, and also the dimerization affinity between the fluorescent proteins could change the apparent on/off rates. This point is not quantified or discussed. Due to the chemical equilibrium analysis, the apparent equilibrium is not only affected by this on/off rates, but also the local availability (concentrations) of the reacting moieties. In the limit where the biosensor concentration is low within a cellular subcompartment or vice versa, how this is going to change the sensitivity of detection because this can push the reaction in either directions. Since equimolar distribution of the moieties are not guaranteed, this affects the detection characteristics of this biosensor. This point should be discussed and emphasized.
Regarding the A/B/C isoforms: We did not mean to claim, that the sensor is specific for RhoA, based on the literature, we are certain it will also bind Rho B and C. We observed binding to active RhoB in an experiment not shown in the manuscript. To make this clearer, we changed the name of the Rho GTPase to Rho.
Regarding the dimerization affinity: Some initial data has been acquired for the weaker dimers Venus and iRFP. They seem to have a slightly beneficial effect but less beneficial than the stronger dimer dTomato. We agree that the biosensor concentration affects the performance (which is an important point with respect to optimizing the right concentration, as will be discussed later). We think that the local availability is not limiting because of fast diffusion of the soluble biosensor. However, this may be an issue in highly polarized cell types such as neurons. This is added to the discussion: '***The biosensor concentration of relocation probes affects their performance. Although the diffusion of a soluble probe will not readily lead to differences in local availability in most cell types, this may be an issue in highly polarized cell types.***'

- Fig 1 A: Are the fluorescence changes of the biosensors due to stimulation with histamine completely reversible ? In other words, is it possible to see a total recovery of the signals with pyrilamine or in the presence of another antagonist ? If not, why?

This is typically what we observe for this antagonist. Although it is added at a saturating concentration, it cannot completely switch of the Rho GTPase activity. This has also been observed with a DORA FRET sensor (Figure 4B in: https://doi.org/10.1124/mol.116.104505)

© 2021. Published by The Company of Biologists under the terms of the Creative Commons Attribution License (https://creativecommons.org/licenses/by/4.0/).

Does histamine stimulation induce a maximal activation of RhoA in HeLa cells? What happens in terms of fluorescence changes when the activity of RhoA is inhibited or in the presence of a Gαq-inhibitor, and in conditions in which RhoA activating GEF, RhoA GAP or RhoA GDI is overexpressed ? Generally, I think it is useful to have a calibration curve of the biosensors activity, maximal/minimal (ON/OFF) response. For exemple, it would help to answer the question concerning biosensors binding affinity for RhoA ("The function of rhotekin is not clear, it seems to lock RhoA in the GTP bound state (Ito et al., 2018; Reid et al., 1996). We can only speculate that rhotekin has a stronger binding affinity for active RhoA than anillin and PKN1 have." (p.15))

We have optimized our system to achieve high Rho activation and this has previously allowed us to do a quantitative comparison of the contrast of RhoA FRET sensors (see supplemental material of: https://doi.org/10.1038/srep14693). Whether this is a maximal response is unclear, but we do observe robust and consistently strong responses, which were not achieved by other strategies.

What is the effect of histamine stimulation on a membrane marker expression/location ?

We propose to perform an additional experiment, measuring the fluorescent intensity for a cytosolic fluorescent protein in the HeLa cell histamine stimulation assay, since we measure the depletion in fluorescent intensity of the sensor in the cytosol.

What is the effect of histamine stimulation on dT2xrGBD biosensor response when this one is forced to be located in other subcellular compartments (mitochondria, nucleus) by fusing the construct to targeting sequences.

We have not tried this experiment and we are not sure what would be the point of that experiment? If the construct would be forced to localize, we would not observe relocalization.

Physiological control: Effect of the presence of the biosensor in cell morphology/behavior... Experimental data concerning this point are evoked in the discussion section. "We demonstrate that low expression of the biosensor, through the truncated CMV promotor, did not inhibit cell division and cell edge retraction. Plus, endothelial cells expressing the sensor still show the typical reaction of contracting followed by spreading, when stimulated with thrombin. Low expression results in a low fluorescent signal of the sensor." (p.16) I think this results would deserve a section in this manuscript.

This is the data shown in Figure 6 we will refer to it more clearly.

- Fig 2D : "The anillin sensor AHD+PH showed a 15% decrease in cytosolic intensity (Figure 2D), but it also relocalizes to striking punctuate structures upon histamine stimulation. These structures did not seem to represent local, high activity of RhoA, as the optimized rGBD sensor in the same cell showed no such locally clustered RhoA activation, but rather a homogenous activation at the membrane and a 60% drop in cytosolic intensity. Similar punctuate structures were observed in endothelial cells, when stimulated with the strong RhoA activator thrombin (Supplemental Movie 5)."
And p. 15 : "However, we noticed that the AHD+PH sensor, containing aGBD, C2 and PH domain, localizes in a punctate manner. These 'dots' were observed in both HeLa cells and endothelial cells and were only observed with the AHD+PH RhoA sensor. As aGBD does not localize in puncta, it seems that the localization is caused by domains other than of the RhoA binding domain, i.e. the C2- and/or PH-domain."
Punctate structures are also present in HeLa cells expressing the anillin sensor before histamine stimulation (see Supplemental Movie 4). Moreover, punctuate pattern activated by thrombin in endothelial cells looks different (more widespread) than the one activated by histamine in HeLA cells. In addition, these structures can also be found in human endothelial cells expressing dT2xrGBD (fig. 6B, Supplemental movie 10). What are those structures thrombin activated in endothelial cells that would be similar to the ones in Hela cells activated by histamine and that "did not seem to represent local, high activity of RhoA"? This is not further commented by the

© 2021. Published by The Company of Biologists under the terms of the Creative Commons Attribution License (https://creativecommons.org/licenses/by/4.0/).

authors.
Very well spotted. What can be seen in Figure 6B and SMovie 10, are different vesicles, that are always observed in endothelial cells expressing fluorescent proteins. We think they are endosomes/lysosomes, which would explain why especially the more pH stable red fluorescent proteins are visible in these structures. They do not localize at the membrane but in the cytosol. These structure are not induced by RhoA activation, and are not present in the TIRF data which excludes the cytosol.

- Fig 3A: "The rGBD sensors solely colocalized in the nucleus with RhoA but not with Rac1 and Cdc42, indicating that rGBD specifically binds constitutively active RhoA." What about dT2xrGBD binding specificity for the three homologues RhoA, RhoB and RhoC?
This point is evoked in the discussion part (p.16) but there is no experimental data to support it "The specificity of the relocation sensor is determined by the binding specificity of the GBD. The rGBD binds the three homologues RhoA, B and C but not to Rac1 and Cdc42". So, why rGBD is presented as a RhoA biosensor?

We apologize for this misunderstanding. We have no reason to assume that the biosensor does not bind all three isoforms. We will refer to the RhoA/B/C isoforms as 'Rho' and we will call it a Rho sensor.

- Fig 3B: The data scatter for the dTomato-2xrGBD is very wide compared to the mScarlet-1xrGBD. What is causing this wide data scatter and such heterogeneous response? This is a problem if the sensor is really so heterogeneously responding to a strong mutant of RhoA, is this a dimerization-dependent problem?
We think that this is related to expression levels. Since dTomato-2xrGBD shows higher amplitudes, the spread also becomes larger and so we think the coefficient of variation will be similar. We will add standard deviations an indicate fluorescent intensity.

- These domain-based biosensors could cause dominant negative/inhibitory artefacts. Also the dimerizing fluorescent proteins could introduce oligomerization of the signaling complex which is not real in cells and clearly affect phenotype. These issues should be tested and addressed by a quantitative measure of cell behavior against increasing concentration/changing dimerization potentials of the biosensor in live cell assays.
We agree that these type of biosensors in a general sense can cause dominant negative/inhibitory artefacts and we explicitly mention this in the text: "Visualizing the endogenous Rho activity may interfere with the biological role of Rho, as the sensor binds endogenous Rho and may compete with natural effectors of Rho"
We were worried about this possible downside and have been very carefully looking at the effects of the biosensor. As highlighted in the manuscript, we noticed mitosis and natural contraction/spreading of endothelial cells. We were able to make stable cell lines. These are all signs that there are no strong negative effects. We also advice to use low expression of the senor to limit negative effects: "To limit the perturbation, the sensor should be expressed at a low level to allow Rho signaling"

- Fig 4 C: "Given the successful improvement of the rGBD-based biosensor by increasing the number of binding domains, we explored whether the same strategy can be applied to the G protein binding domains from PKN1 and Anillin" and "The dimericTomato-2xrGBD sensor shows the best relocation efficiency, with a median change in cytosolic intensity of close to 50%"... So why the dT-2xaGBD construct has not been tried ?
Because we did not see the stepwise improvement as we saw for the rGBD sensor, so we do not expect an improvement in that construct. Plus, the cloning for the 2xaGBD was initially not working out.

- p.9 : "None of the pGBD sensors showed a clear membrane localization upon stimulation with histamine (Figure 4A). The increase in cytosolic intensity observed in some cells, seems to be caused by changes in cell shape." Do changes in HeLa cell shape induced by histamine stimulation? How this can be explained? Do some cells expressing the rGBD sensors (single, tandem and triple and dimericTomato) undergo these changes of shape too, upon histamine stimulation? If yes, to what extent these changes in cell shape affect signals?

© 2021. Published by The Company of Biologists under the terms of the Creative Commons Attribution License (https://creativecommons.org/licenses/by/4.0/).

The activation of Rho GTPases by the histamine receptor often results in changes in cell shape in HeLa cells.
We propose to perform an additional experiment with a cytosolic fluorescent protein in the HeLa cell histamine stimulation assay, to measure potential intensity changed solely caused by shape changes.

- p9: Overall, the paragraph about Fig 4 E,F is not clear. What amino acid sequences of G Protein Binding Domains of Anillin and PKN1 bring for the understanding of rGbD, aGBD and pGBD sensors?
Since there is no crystal structure for rGBD available, we thought it is interesting to compare the amino acid sequences to see how similar/ different these domains are.

- p. 12, Fig 6C, Fig. 6E: "The membrane marker showed a relatively small increase in intensity after stimulation and the curve did not show the same pattern as the RhoA biosensor intensity curve. Therefore, we conclude that the increase in RhoA biosensor intensity is caused by relocalization." It surprises me that decrease in cell areas induced a very small increase in fluorescence intensity of the membrane marker. It would be very helpful to see a figure with a quantification of the membrane marker intensity changes during this process. What about a cytoplasmic marker?
Figure 6D shows the intensity measurements of the membrane marker intensity. The small change can be caused by membrane changes, but also other factors that affect intensity (focus change). We will add the membrane intensity measurements to Figure 6F and G as well. Since these measurements are made in TIRF, the intensity of the cytoplasmic marker would be very low. Therefore, we decided to use a membrane marker.

In addition, how does the movement artefact is corrected?

The ROIs were drawn by hand to measure the fluorescence intensity.

"Our data revealed that the RhoA biosensor displays RhoA activity at subcellular locations where RhoA activity is expected, and appears mostly independent of fluorescent intensity measured by a separate membrane marker." This part should be developed further. Are there examples of cells for which the biosensor activity is dependent on fluorescent intensity measured by a separate membrane marker?

The intensity of the membrane marker is only affected by changes in membrane area or morphology (and other technical reasons that lead to a change in intensity, e.g. focal drift, bleaching). This point is made in the paper by Dewitt that we cite (https://doi.org/10.1083/jcb.200806047). We are not aware of papers that show biosensor activity dependent on a separate membrane marker. One potential confounding issue is quenching of the membrane marker by FRET, but this would lead to a decrease in intensity and we do not observe that.

- Discussion (p.16): "Comparing relocation sensors to FRET sensors, both have their own advantages and disadvantages." The dT2xrGBD sensor is here presented as a new relocation sensor for RhoA activity. However in general, there should be more development of the direct comparisons, pros and cons, with quantitative data or more details allowing to have a general overview of the advantages and disadvantages of this new relocation biosensor as compared to the existing ones.
We explain the pros and cons of FRET sensors and relocation sensors in the introduction and we show a quantitative comparison of this new relocation biosensor as compared to existing relocation biosensors (figure 2). The advantage of the relocation sensor relative to a FRET sensor is highlighted in the discussion: ***"Furthermore, the relocation sensor requires confocal microscopy or TIRF microcopy to spatially separate the bound from unbound probe, whereas FRET measurements are usually performed with widefield microscopes. However, the former mentioned techniques usually offer the higher resolution. Here we presented previously unachieved visualization of Rho activity at subcellular resolution. We observed local activation of Rho at the Golgi which was not possible with the DORA RhoA FRET sensor (Van Unen et al., 2015), indicating a higher sensitivity of the relocation sensor."***

© 2021. Published by The Company of Biologists under the terms of the Creative Commons Attribution License (https://creativecommons.org/licenses/by/4.0/).

Minor points:
- Overall, scale bars should have to be included in HeLa cells microscopy images.

We will provide the width of the image in the figure captions.

- It was not clear until the Methods section that the widefield analysis appeared to be normalized against another fluorescent protein-based cytoplasmic signal to correct for variations in cell volume. I think this point should be mentioned in the main text more prominently and emphasized so that readers are not misled.
The normalization of time traces has been done to account for differences in the initial intensity (e.g. due to differences in expression level), this is now better explained: ***"The mean gray value or cell area respectively, were normalized by dividing each value by the value of the first frame, to account for differences in the initial intensity."***

Of note, there is no extra cytoplasmic signal to correct for variations in cell volume.

- p. 9 : "Anillin AH+PH sensor" instead of "Anillin AHD+PH sensor"

Corrected.

- Fig 2B and 2D : Explain what parameter is used for the normalization of each signals ?

We state in the methods: ***" The mean gray value or cell area respectively, were normalized by dividing each value by the value of the first frame, to account for differences in the initial intensity."***

- Fig. 1A, top panel: it would be good to know which images correspond to the addition of histamine and which ones correspond to the addition of pyrilamine

The time line with the grey bars indicating the stimulus of the graph matches the images. We changed the legend to clarify: *"The images match with the perturbation that is indicated for the plot in panel C."*

- "TRIF microscopy" is written in legends of Fig. 6 and of Supplemental movie 11, and in Materiel and Methods section p. 23
- Fig. 3 legend: Correct "mScralet-I-1xrGBD"
- Fig 4F, legend: " Anillin and the bound RhoA are depicted in dark and light yellow, respectively. PKN1 and the bound RhoA are depicted in light and dark blue, respectively." Color codes in legend are opposites to the figure ones.
- p.11 : "To examine this, we used a rapamycin-induced hetero dimerization system to recruit the dbl homology (DH) domain, of the RhoA activating GEF p63, to the membrane of the Golgi apparatus." Corresponding references should be included.

Thanks for pointing these out, all have been addressed/corrected.

- Fig. 5A : Explain FRB, Fig 5C : no unit for a ratio
We changed the legend ***"A) Still images of HeLa cells expressing FRB (part of rapamycin hetero-dimerization system) anchored to the membrane, Golgi and mitochondria (first column), FKBP-p63-DH (counterpart of rapamycin hetero-dimerization system, not shown), localization of the dimericTomato-2xrGBD sensor pre activation (second column) and post activation with 100 nM rapamycin (third column)."***

Reviewer #1 (Significance (Required)):

Mahlandt et al. optimized and compared several G protein binding domain (GBD)-based biosensors in order to improve the potential of existing RhoA-domain-based biosensors for visualizing and reporting RhoA subcellular activity in living cells and tissue. The authors demonstrate that fusing a dimerizing fluorescent protein to the rhotekin GBD (rGBD) is an

© 2021. Published by The Company of Biologists under the terms of the Creative Commons Attribution License (https://creativecommons.org/licenses/by/4.0/).

efficient strategy to increase the brightness of the sensor. The use of Rhotekin-RBD as affinity domain for Rho-class of GTPase is very well established, both in the methods of affinity pulldowns and in biosensor designs for Rho-class of GTPases in the field. The authors show that the dimericTomato-2xrGBD biosensor can indicate endogenous RhoGTPase spatial activity in dividing HeLa cells and during cell retraction of human endothelial cells.

The dimericTomato-2xrGBD biosensor is thus introduced and described as a RhoA localization-based biosensor, however no experimental data demonstrate the binding specificity of the biosensor for RhoA. Moreover, authors discuss about a previous work showing that rGBD binds the three paralogs RhoA, RhoB and RhoC. This point and the apparent singular claim of this biosensor reporting RhoA activity as this manuscript alludes to are inappropriate and misleading.

We apologize for the misconception that this probe is specific for RhoA. We do not want to claim this sensor is specific for RhoA (and note that we have been involved in generating FRET biosensors for the different isoforms, RhoA/B/C ourselves: https://doi.org/10.1038/srep25502). We have addressed this in the introduction, and we have changed RhoA to Rho to better reflect that we are looking at all three isoforms.

This point especially in light of the field has moved on in the past 20 years to assign more specificity (not less) to which GTPase the biosensors are being specific, i.e., via FRET, etc., significantly tempers the enthusiasm of this reviewer. In addition to this main issue, the incomplete characterization of the relative affinities of the domain to the target GTPase isoforms and of the dimerization affinities of the fluorescent proteins (which could change the apparent reaction rate constants), and the impact of which on the reversibility, oligomerization states and detection sensitivity, and the biology, also appeared lacking. Additional stoichiometric considerations and apparent reaction equilibrium that are impacted by the relative concentrations of interacting moieties require careful and further analyses, study and discussion.
In general, I think that this work could be interesting to a more specialized field audience with further analyses of the affinities of the interacting moieties and better characterization of the behavior of this biosensor in living cells since it is likely causing oligomerization of the signaling units due to the forced dimerization of the detection unit.

Referees cross-commenting
This is a dimerizing probe. It gets pretty bulky. Is dimerization occurring prior to GTPase binding or after? Is the dimerized probe/GTPase complex somehow more stable than would otherwise be if they were monomeric? If so, how would that affect the lifetime of the detection and also the diffusivity of the probe("s", if already dimerized) and possibly the whole oligomer? **dTomato is** shown to be a strong, obligate dimer. Therefore, we assume that the fluorescent probe is present as a dimer before (and after) binding to the GTPase.
With respect to size/bulkiness we'd like to note that the biosensor is only somewhat larger than a FRET sensor, i.e 2x47 kDa and 74 kDa, respectively.

It still feels to me that, yes new brighter fluorescent proteins were used, and dimerization and multimerization of the signaling complex increased the SNR of the system, but the whole premise just reverted the biosensor field back 20yrs, which has been my biggest single concern regarding this paper.

This evaluation is in our opinion largely based on the misconception that we claim RhoA specificity. We do not claim that this sensor is specific for RhoA (and we have revised the manuscript accordingly) and we are not aiming to replace FRET sensors (being quite fond of FRET sensors as is clear from our previous work).
We think that there is ample opportunities and applications for the improved relocation sensor (as is also evident from requests for the plasmids that encode the probe), for instance in experiment were FRET sensors are challenging to use, such as optogenetics experiments and multiplexing biosensors. We state in the discussion: "***Single color relocation sensors are ideal candidates for multiplexing experiments. Plus, the growing field of optogenetics is in need of single color biosensors to detect the effect of optogenetic perturbations. The conventional CFP-YFP FRET sensor is incompatible with most, blue light induced optogenetic tools.***"

© 2021. Published by The Company of Biologists under the terms of the Creative Commons Attribution License (https://creativecommons.org/licenses/by/4.0/).

Reviewer #2 (Evidence, reproducibility and clarity (Required)):
Summary:

Visualization of subcellular activity of GTPases is critical for the understanding of signal transduction of cell growth, differentiation, morphogenesis, etc. For this purpose, researchers often use relocation probes, which comprise a fluorescent protein(s) and a GTPase-binding domain(s), and move from cytosol to the location of active GTPases. The authors improved a previously reported RhoA probe with a strategy of increasing the avidity of RhoA-binding domain and optimizing the fluorescent protein. In the beginning, the authors declare "the relocation of the original, single rGBD monomeric fluorescent protein sensor is hardly detectable" in HeLa cells. To overcome this problem, they developed six constructs by changing the number of rGBD (rhotekin GBD) domains and fluorescent proteins. They found that the increase in the number of rGBD and a dimeric prone fluorescent protein, tdTomato, generate a better probe for RhoA activity. The specificity was examined by using active Rac1 and Cdc42 proteins. Different RhoA-bind domains derived from Rhotekin, PKN1, and Anillin were compared to show the superiority of rhotekin GBD. Finally, they show that subcellular RhoA activation detected by the probe is consistent with the knowledge on RhoA activation by using vascular endothelial cells. Overall this work has been well done in an organized way and disclose a novel RhoA probe that will be useful in future research of RhoA.

Major comments:

1. Reproducibility: The number of analyzed cells is described in the legend, but the number of independent experiments is not shown. This is critical to evaluate the reproducibility of the data. Preferably, the data should be presented to show data set derived from each trial clearly. It should also be described how cells were selected for the analysis? It is also preferable to apply automatic analysis. Ideally, the raw data with code sets for analysis should be presented.
We will indicate the independent experiments. ROIs were partly drawn by hand. We agree that segmentation based methods would increase reproducibility, but this data set is not suitable for automated analysis.

2. A serious defect of the relocation probe is the dependency on the expression level. The lower the number of the probe in a cell, the higher the fraction of recruited to active RhoA. However, lowering the probe concentration will be accompanied by dim fluorescence. The authors should describe how the optimal expression level was achieved.
We fully agree. Using the low expression promoter improved the dynamic range but we have not gained control over the optimal expression level. It does vary from cell to cell. We added this paragraph to the discussion: "***However, the optimal expression level is crucial for the dynamic range of the relocation sensor. Low concentrations of the sensor will show higher levels of relocalization, as a larger fraction of the sensor molecules binds the limited, active, endogenous Rho molecules. Nevertheless, if the concertation of sensor is too low, the fluorescent signal cannot be detected. To optimize the expression level, the CMVdel promoter, leading to a lower expression level, was applied (Watanabe and Mitchison 2002). Even though, this minimal promoter improved the performance of the relocations sensor, a variety*** *of expression levels was observed. Cell sorting could be applied to select for cells with the optimal expression level.*"

3. Statistical analysis is absent throughout the paper.
We will add standard deviations to the dot plots.

Minor comments:

1. In Figure 1, mNeonGreen (mNG) was used as the fluorescent protein fused to rGBD instead of EGFP, which was used in the original paper. For a fair comparison with the previous report, analysis using the original probe, i.e., EGFP-rGBD, is desirable. Or, the author may simply tone done.
That is a good point. We propose to perform the HeLa cell histamine stimulation assay for the eGFP-rGBD sensor and add the data to Figure 1B.

© 2021. Published by The Company of Biologists under the terms of the Creative Commons Attribution License (https://creativecommons.org/licenses/by/4.0/).

2. In the introduction, it says " The RhoA FRET sensors achieve subcellular resolution to a certain extent, but due to their design they do not localize as endogenous RhoA". Reference is required.
We changed the following in the introduction: *The RhoA FRET sensors achieve subcellular resolution to a certain extent, but due to their design they may not localize as endogenous RhoA (Michaelson et al., 2001).*

3. rGBD should be rhotekin GBD. It should be clearly stated in the beginning.
We wrote in the introduction: "Secondly, the rhotekin G protein binding domain (rGBD)-based eGFP-rGBD Rho sensor, that was reported in 2005 (Benink & Bement, 2005)." and in the results " The eGFP-rGBD biosensor consists of an enhanced green fluorescent protein (eGFP) and a rhotekin G protein binding domain (rGBD)."

4. The reason why the CMVdel promoter is used should be stated clearly.

Thanks for the suggestion. We added to the discussion:
"However, the optimal expression level is crucial for the dynamic range of the relocation sensor. Low concentrations of the sensor will show higher levels of relocalization, as a larger fraction of the sensor molecules binds the limited, active, endogenous Rho molecules. Nevertheless, if the concertation of sensor is too low, the fluorescent signal cannot be detected. To optimize the expression level, the CMVdel promoter, leading to a lower expression level, was applied (Watanabe and Mitchison 2002). Even though, this minimal promoter improved the performance of the relocations sensor, a variety of expression levels was observed. Cell sorting could be applied to select for cells with the optimal expression level."

5. Page 23: TRIF should read as TIRF.
Corrected

6. Figures: Grey letters should be avoided.
We will verify the figures for readability

7. Fig. 3A: Apparently the probe binds to Rac1 G12V to some extent. The discrepancy of RhoA localization between mSca-1xrGBD and dt-2xrGBD must be discussed. This observation clearly suggests that GBD may change the localization of RhoA. It is interesting to note that Rac1 and RhoA may localize to the nucleolus.

We have changed the text to make clear that the dTomato-2xrGBD binds better to RhoA than the 1xrGBD variant: "Comparing the original single rGBD sensor (mScarlet-I-1xrGBD) with the dimericTomato-2xrGBD sensor, a higher nuclear to cytosolic intensity ratio for the multi- domain sensor was detected, supporting its higher affinity for RhoA."

Reviewer #2 (Significance (Required)):

1. This work discloses an improved RhoA probe, which will be welcome by the researchers in the field of small GTPases.

We are glad that the reviewer shares our enthusiasm

2. Novelty of increased GBD: The idea of increasing the GTPase-binding domain in the relocation probe was reported some time ago: Augsten et al., Live-cell imaging of endogenous Ras-GTP illustrates predominant Ras activation at the plasma membrane. EMBO Rep. 7, 46-51 (2006)
Agreed - we added the reference to the discussion: "This strategy, to utilize multiple repeating domains has also been effective for a PH domain based lipid sensor and a cRAF derived Ras-binding domain Ras activity sensor (Augsten et al., 2006; Goulden et al., 2018)"

3. Novelty of rhotekin GBD: The reason why GBD of PKN is chosen in intramolecular FRET

© 2021. Published by The Company of Biologists under the terms of the Creative Commons Attribution License (https://creativecommons.org/licenses/by/4.0/).

biosensors such as DORA and Raichu is that the affinity of other GBD's is too high [Table 1, Yoshizaki et al., J. Cell Biol. 162, 223-232 (2003)]. Judging from this old data, GBD's of mDia and Rhophilin, may work better than that of Rhotekin. Moreover, it is known that PH domain may be required for proper conformation of GBD's. Thus, it is not surprising that removal of PH domain from the Anillin probe abolishes its translocation ability. Therefore, to the reviewer's eyes, the choice of GBD in Figure 4 is biased to those that will work less efficiently.

We see the point, but we have chosen these (PKN/anillin) for a practical reason, namely that we had cDNA encoding these probes in our lab. We thank the reviewer for the suggestion to look into other GBDs.

4. Authors' proposal of "systematic optimization" sounds exaggerated, considering the small number of constructs tested in Fig. 1 and Fig. 4. Similarly, it is not clear whether dimerize prone-fluorescent proteins are better choice by simply comparing tdTomato and mNeonGreen.

Fair enough, we think of it as a systematic comparison (figure 1) and we have rephrased the sentence: ***"Improving the rGBD probe by increasing the avidity was successful"***

5. Keywords of expertise: Fluorescent probes. Cell signaling.

Referess cross-commenting

Because Review Commons does not specify the journal to be published, the request by the Reviewer #1 sounds too much. The probe reported in this work deserves publishing, although it may not be a ground-breaking probe.

We thank the reviewer for the encouraging words and support.

Reading the comments by the other reviewers, following concerns should be cleared.

1. Relationship between the probe's concentration and the response.

2. Specificity to RhoA, RhoB, and RhoC

3. The effect of the cell morphology as pointed by Reviewer #1.

Concern 1 will be addressed by re-analysis of the data.
Concern 2 is addressed by changes in the text, was we have indicated in our response. Concern 3 will be addressed by control experiments that look into changes in cell morphology

To Reviewer #1

-Since equimolar distribution of the moieties are not guaranteed, this affects the detection characteristics of this biosensor. This point should be discussed and emphasized
The probe will diffuse rapidly within cytosol. Therefore, subcellular concentration of the probe may not affect significantly on the performance of the probe.

-What is the effect of histamine stimulation on dT2xrGBD biosensor response when this one is forced to be located in other subcellular compartments (mitochondria, nucleus) by fusing the construct to targeting sequences.
I did not understand this question quite well.

Reviewer #3 (Evidence, reproducibility and clarity (Required)):
Summary

In this paper, Mahlandt et al compared and improved relocation sensors to visualize the activity of endogenous Rho. As a result of screening for several Rho binding domains (GBDs) and the number of repeats, the authors found that dTomato-2xrGBD is optimal, and succeeded in visualizing the activity of Rho during cytokinesis and migrating cells.

© 2021. Published by The Company of Biologists under the terms of the Creative Commons Attribution License (https://creativecommons.org/licenses/by/4.0/).

Overall, this sensor would be a useful tool for many cell biologists. The data are represented clearly in the figures. I provide some concerns; that would be worth addressing in a revised version.

Major comments

1. The authors should experimentally show the quantitative relationship between biosensor expression level and degree of relocation. In principle, this relocation type sensor binds to the endogenous GTP-bound Rho. Since the number of endogenous GTP-bound Rho is limited in cells, the degree of relocation is considered to be dependent on the expression level of the sensor. If the number of biosensors expressed is too small in a cell, the response will be saturated. If the number of biosensors is too large, the relocation will be weakened and the Rho signal will be suppressed. Furthermore, although a weak promoter is used, the heterogeneity of the expression level in each cell makes quantitative analysis difficult, especially in transient expression experiments. I would like to suggest the addition of quantitative experimental data.

We propose to re-analyze of our data, indicating the relative expression levels of the biosensor (based on intensity) in the dot plots. We agree that the expression level potentially affects sensor performance and we will address this more clearly in the text
We added to the introduction: "*A potential drawback is that background signal of the unbound biosensor in the cytosol, which may occlude the bound pool and reduce the dynamic range.*"
We added to the discussion: "***However, the optimal expression level is crucial for the dynamic range of the relocation sensor. Low concentrations of the sensor will show higher levels of relocalization, as a larger fraction of the sensor molecules binds the limited, active, endogenous Rho molecules. Nevertheless, if the concertation of sensor is too low, the fluorescent signal cannot be detected. To optimize the expression level, the CMVdel promoter, leading to a lower expression level, was applied (Watanabe and Mitchison 2002). Even though, this minimal promoter improved the performance of the relocations sensor, a variety of expression levels was observed. Cell sorting could be applied to select for cells with the optimal expression level.***"

2. Most of the time-series data show only a representative example, namely, N = 1. In relation to the aforementioned issue, data and distribution derived from several cells (e.g. SD) should be shown in a clear manner.
We focused not primarily on the kinetics, but more on maximal relocation, therefore we do not have time lapse movies for all the shown data points (e.g. a time lapse is shown in 1C and the data for a higher number of cells is shown in 1B). However, we can provide time series for multiple cells from our existing data sets.

Minor comments

3. I hesitate to call the biosensor developed in this study "RhoA sensor". This is because, as the authors mention, it has been reported that the rGBD also binds to RhoB and RhoC. If the authors call it a RhoA sensor, they should investigate the specificity of binding to RhoB and

RhoC in addition to RhoA. If not, I would like to suggest changing the name to "Rho sensor" instead of "RhoA sensor".
This is a fair point, also made by other reviewers. We will change the name to Rho sensor.

Reviewer #3 (Significance (Required)):

Rho is one of the low molecular weight G proteins, which regulate the reorganization of the actin cytoskeleton. As biosensors for visualizing the activity of Rho proteins, it has been reported intramolecular and intermolecular FRET biosensors and relocation sensors. The latter is less widely used than the former, because of insufficient sensitivity and specificity. Therefore, the improvement of Rho biosensors is really important and needed in the community of cell biology research field. The importance of this manuscript, I believe, is that the authors compared the existing relocation type Rho sensors. This is informative.
Rho is one of the low molecular weight G proteins that regulate the rearrangement of the actin cytoskeleton. Intramolecular and intermolecular FRET biosensors and relocation sensors have

© 2021. Published by The Company of Biologists under the terms of the Creative Commons Attribution License (https://creativecommons.org/licenses/by/4.0/).

been reported as biosensors for visualizing the activity of Rho proteins. The latter is not as widely used as the former due to its inadequate sensitivity and specificity. Therefore, improving the Rho biosensor is very important and is needed by the community in the field of cell biology research. I believe the importance of this manuscript is that the author compared existing relocation-type Rho sensors. This is beneficial and informative.

My expertise: Cell biology, live-cell imaging, development of genetically encoded fluorescent probes

We thank the reviewer for the positive evaluation of our work.

Referees cross-commenting

I generally agree with Reviewer 2's opinion. The opinions of our three reviewers can be summarized in three points: expression level, specificity, and statistical analysis and representation. I think these should be asked to the authors as major critics that should be addressed before publication.

We agree and we propose to address the three main points (see also response to reviewer 2).

Reviewer #4 (Evidence, reproducibility and clarity (Required)):

SUMMARY:

Mahlandt and colleagues use advanced microscopy techniques to test new configurations of several Rho relocation sensors, which report on the activity of members of the endogenous RhoA

GTPase family of proteins. A novel variant containing the dimericTomato fluorescent protein and a double rGBD domain shows a substantial increase in dynamic range in comparison with 2 originally published sensors and other new variants they tested.
They use a cellular assay to show that this novel variant is specific for the activity of Rho family of Rho GTPases and not the Cdc42/Rac families. Finally, the authors show that this new variant can be used to measure a specific localised increase of Rho activity at the Golgi, and during cell division and cellular morphology changes that are known to activate the RhoA family of Rho GTPases. The biosensor can be useful for the community. However, I think the paper is not well written (I was very confused by several statements). The manuscript should be thoroughly proofread, there are quite some unclear or duplicate passages (for examples, see "text comments" below). Currently this hampers the interpretation of the manuscript for the reader. The authors are very dogmatic - they make claims about the literature that I do not agree with at all. Some of these unbalanced views will confuse the non-expert readers.

MAJOR COMMENTS:

-The reported dTomato-rGBD sensor is unable to distinguish between the different members of the RhoA familiy of Rho GTPases (measures combined activity of RhoA, RhoB and RhoC), which is unclear for the reader in the current text phrasing in the introduction. The authors seemingly suggest throughout the manuscript to work with a specific RhoA biosensor, which is not the case. This strong statement is completely misleading. The authors need to refer to the biosensor being specific for Rho (RhoA,B,C) GTPases versus Rac1/Cdc42 biosensors, and discuss what this means for the field. Some discussions about this are made in a JCB paper by Graessl et al, that the authors also cite.
We agree that the probe measures the combined activity of all three isoforms and apologize for the confusion. We have changed the name to Rho sensor and updated the manuscript.

-If the authors really want to sell that the biosensor is only specific for RhoA, then they need to make a series of experiments with RhoB and RhoC dominant positive/negative constructs, to tackle that specific point.
No, we did not intend to claim the sensor is specific for RhoA in comparison to Rho B and C.

-Did the authors consider to use the artificial GBD from Keller, 2019 to make a specific

© 2021. Published by The Company of Biologists under the terms of the Creative Commons Attribution License (https://creativecommons.org/licenses/by/4.0/).

relocation sensor for RhoA? Perhaps the authors can comment on the feasibility of this approach?
We think that this might be the only way to make a specific RhoA relocation sensor. Recently, we have received the DNA and plan to do the histamine stimulation experiment in HeLa cells as in Figure 1B.

-A strong (dogmatic) statement is that Rho GTPases FRET sensors report solely on the activity of GEFs. This is not the case, these sensors report on the flux of GAP and GEF activity for Rho GTPase in cells. This is also true for relocation sensors, and has been documented in work from the Bement/Pertz/Nalbant/Dehmelt labs.
We thank the referee for this correction and we have changed the text to: "*By design, these* ***FRET sensors report on the balance between activating guanine exchange factors (GEFs) and inactivating GTPase-activating proteins, instead of visualizing endogenous RhoA-GTP***"

-From the data in Figure 1, it seems to follow that the efficiency of PM relocation is mainly determined by the number of rGBD modules on the sensors. Could the authors speculate on how this works in practice; is the multi-rGBD sensor increasingly kinetically trapped by a single RhoA molecule, or is the sensor mostly bound to multiple RhoA molecules at the PM?
This is an interesting question to which we do not have an answer. We added some text to the discussion: "***It is currently not clear how each of the GBDs of the dimericTomato-2xrGBD sensor contribute to Rho binding and the probe may bind anywhere between 1 and 4 Rho molecules. If the probe is capable of binding multiple Rho proteins, the binding efficiency will depend on the local density of Rho in the membrane.*** "

-Some form of statistical analysis should be performed on the data to give the reader a sense of robustness of the findings and its uncertainty. Either a non-parametric test on the median, confidence intervals or e.g. boxplots showing notches.

We will include standard deviations in our dot plots.

-Time-series now show single example traces (fig1C, fig2B,D, fig5B). It would be informative for the reader if the curves of all experiments were plotted, and statistical analysis would be performed on the data. It is unclear how representable the kinetics in these curves are.

We can show the kinetics for more examples but we did not acquire time lapses for all the data points shown in the dot plots, since the microscope could not move fast enough to acquire frames with an interval of 10 -20 s.

-About the spatial patterns of Rho activity (cytokinesis, tail retraction, ...), the reviewers agree that statistical analysis is much more difficult. But maybe showing 2-3 cells instead of only one, would make the data more convincing.

We will provide more examples.

MINOR COMMENTS:

-(fig4a) dTomato-2xpGBD, why is this not good? how is it possible that it binds good to nucleus, but no translocation is observed? const activity? expression levels?

We were surprised and somewhat disappointed by this as well and we do not have an explanation, besides that the binding affinity required for dynamic relocation seems to be higher than the one for binding the overexpressed active Rho GTPase.

-(fig4f) The aGBD/pGBD binding sites for RhoA show great overlap but bind to completely different sites at RhoA, is this correct? (color scheme used for the structures is not easily interpretable)

It is correct they both have two binding sites but apparently, they found crystals for one or the other. Maesaki et al. 1999 is describing the two binding site. We will change the colors.

© 2021. Published by The Company of Biologists under the terms of the Creative Commons Attribution License (https://creativecommons.org/licenses/by/4.0/).

-(fig5) Unclear how the intensity at the specific organelles is measured? were the organelles segmented or hand-drawn ROI based? The quantified difference is very small, no statistics are performed, and it is unclear how it was measured. This is currently weak evidence for the main claim in this subsection.
ROIs are drawn by hand. We will provide standard deviations in our dot plots.

-(fig5) The kinetics of the response to histamine (fig1C) seems to be much faster as the rapamycin mediated increase in fig5B for the PM condition. Any explanation for this? Why does it not reach a plateau like in the histamine experiments?

It is probably the recruitment of the p63-DH that takes more time than the activation of the H1R and the downstream signaling. We have the data of the p63-DH recruitment channel so we will check the recruitment kinetics of the p63-DH to the membrane.

-(fig6F) Data from 6D is repeated here, 6F could potentially show aggregate time-series instead of individual cells. Would also improve interpretation if the membrane marker curve is plotted in every subfigure. Potentially membrane marker intensity could be used to normalise the (TIRF) measurements?

We will include the data of the membrane intensity for every trace in F.

-can the authors provide scale bars on the micrographs, as is usually done in any manuscript ? It would also be useful to put time labels when images corresponding to timeseries are shown.

We will provide the width of the image in the figure captions.

-ratio values are dimensionless by definition, so no need to write "arbitrary units"

We will change that.

TEXT COMMENTS:

-(abstract): "Due to the improved avidity of the new biosensors for RhoA activity, cellular processes regulated by RhoA can be better understood." -> unclear what the authors mean with 'avidity' in this context? (here, and throughout rest the manuscript)
Avidity refers to "*the accumulated strength of multiple affinities*", we added this explanation to the text in the introduction. Another paper working with multiple biding domains to improve a relocation sensors also calls it avidity: A high-avidity biosensor reveals plasma membrane PI(3,4)P2 is predominantly a class I PI3K signaling product (Goulden at al. 2018 JCB).

-(introduction) "Although these three Rho GTPases may have different functions, we generally refer to RhoA in this manuscript." -> unclear what message the authors try to convey with this sentence.
We changed to: "We will use 'Rho' throughout the manuscript, which refers to all three isoforms"

-(introduction) "Active RhoA mainly localizes at the plasma membrane, due to its prenylated C-terminus" -> where else would it be localised? Where is inactive RhoA localised?

We included: ***"Active Rho mainly localizes at the plasma membrane, due to its prenylated C-terminus (Garcia-Mata et al., 2011).However, a fraction of RhoA has been found at the Golgi apparatus. Inactive RhoA, in comparison, can be extracted from the plasma membrane by Rho-specific guanine nucleotide dissociation inhibitors (RHOGDIs) (Garcia-Mata et al., 2011)".***

-(introduction) "Unimolecular Rho GTPase FRET-based biosensors consist of the Rho GTPase itself, a GBD and a FRET pair." -> a short description/explanation of what a "FRET pair" is would benefit the non-specialised audience.

© 2021. Published by The Company of Biologists under the terms of the Creative Commons Attribution License (https://creativecommons.org/licenses/by/4.0/).

We included: "*Unimolecular Rho GTPase FRET-based biosensors consist of the Rho GTPase itself, a GBD and a FRET pair, which is commonly a cyan and a yellow fluorescent protein.*"

-(Results p9) "For the original Anillin AH+PH sensor...around 15%" -> did the authors do the experiment with G14V on this original sensor variant?
Yes, it is supposed to say AHD+PH here as well, which has been corrected. We performed the experiment with mScarlet-AHD-PH.

-(Results p9) The "mScarlet-I-AHD+PH" seems to perform quite good on the fig4D assay, but is not present in 4C analysis?
eGFP-AHD+PH was used as the original sensors for the 4C assay. Due to the color of the RhoA G14V (mTq2) we switched to the mScarlet version to exclude bleed through. We assume that the sensor performs similar with different monomeric fluorescent proteins.

-(Results p9) "mScarlet-I-AHD+PH" is the same as "AHD+PH (aGBD+C2+PH)"? descriptions unclear. Would generally advise to thoroughly check the manuscript for consistency of condition descriptions / abbreviations in both text and legends.
Changed to: AHD+PH (consisting of aGBD+C2+PH).
We mention earlier: "Moreover, a published relocation sensor AHD+PH based on Anillin contains, next to a G protein binding domain, also a C2 and a PH domain and localizes in punctuate structures which do not represent Rho activity (Figure 2C,Supplemental Movie 4 and 5) (Munjal et al., 2015; Piekny & Glotzer, 2000). Here, we used only the G protein binding domain of Anillin (aGBD) as a basis for another sensor."

-(Results p12) "Visualizing endogenous RhoA activity" as subsection title could potentially confuse readers, since all measured Rho activity in the manuscript is endogenous.
That could indeed be confusing. What we intending to highlight is that we did not overexpress any signaling molecules or receptors in these experiments. We changed the title to: "Visualizing endogenous Rho activity under physiological conditions"

minor text:

-(fig3b legend) "mScralet-I-1xrGBD"
Corrected

-(fig6H legend) "TRIF", and "cbBOEC" is same as "BOEC"?

It is a detail, but these are indeed different and we have updated the materials and methods to better reflect this: "*cord blood Blood Outgrowth Endothelial cells (cbBOEC)*" and "*Blood Outgrowth Endothelial cells from healthy adult donor blood (BOEC)*"

Reviewer #4 (Significance (Required)):

The novel "Rho" family GTPase relocation sensor that the authors present might be a significant improvement over the currently existing ones (for refs, see manuscript). This might provide a substantial technical advance in the field and increases the utilisation and the reproducibility of this tool in the field. This sensor will be of significant interest for the Rho GTPase signalling field, and more broader the cytoskeleton biology community. My expertise in Rho GTPase biology, biosensor development and advanced microscopy granted me the opportunity to judge the complete manuscript

The reviewer thinks that the new sensor will be of significant interest and we agree.

© 2021. Published by The Company of Biologists under the terms of the Creative Commons Attribution License (https://creativecommons.org/licenses/by/4.0/).

Revision Plan

Major points:

Expression level (Relationship between the probe's concentration and the response):

Reveiwer1: These domain-based biosensors could cause dominant negative/inhibitory artefacts. Also the dimerizing fluorescent proteins could introduce oligomerization of the signaling complex which is not real in cells and clearly affect phenotype. These issues should be tested and addressed by a quantitative measure of cell behavior against increasing concentration/changing dimerization potentials of the biosensor in live cell assays.

Reviewer 3: The authors should experimentally show the quantitative relationship between biosensor expression level and degree of relocation. In principle, this relocation type sensor binds to the endogenous GTP-bound Rho. Since the number of endogenous GTP-bound Rho is limited in cells, the degree of relocation is considered to be dependent on the expression level of the sensor. If the number of biosensors expressed is too small in a cell, the response will be saturated. If the number of biosensors is too large, the relocation will be weakened and the Rho signal will be suppressed. Furthermore, although a weak promoter is used, the heterogeneity of the expression level in each cell makes quantitative analysis difficult, especially in transient expression experiments. I would like to suggest the addition of quantitative experimental data.

Reviewer 2: A serious defect of the relocation probe is the dependency on the expression level. The lower the number of the probe in a cell, the higher the fraction of recruited to active RhoA. However, lowering the probe concentration will be accompanied by dim fluorescence. The authors should describe how the optimal expression level was achieved.

Action: We will indicate expression level in our graphs by color coding or size of the dots, correlating with the measured fluorescents intensity. We will plot the correlation between fluorescent intensity and relocation for the dT-2xrGBD sensor in HeLa cells stimulated with histamine.

Specificity to RhoA, RhoB, and RhoC

This is a misunderstanding, we did not mean to claim that the sensor is able to differentiate between RhoA/B and C. We will call it Rho sensor.

Cell morphology

Reviewer1) - p.9 : "None of the pGBD sensors showed a clear membrane localization upon stimulation with histamine (Figure 4A). The increase in cytosolic intensity observed in some cells, seems to be caused by changes in cell shape." Do changes in HeLa cell shape induced by histamine stimulation? How this can be explained? Do some cells expressing the rGBD sensors (single, tandem and triple and dimericTomato) undergo these changes of shape too, upon histamine stimulation? If yes, to what extent these changes in cell shape affect signals

Reviewer2) The effect of the cell morphology as pointed by Reviewer #1

Reviewer 1: What is the effect of histamine stimulation on a membrane marker expression/location ?

Action: We propose to do an additional experiment, image a cytosolic fluorescent protein (dimericTomato or mNeonGreen) in the HeLa cell histamine stimulation assay, to measure the effect of cell shape on the fluorescent intensity. This data can be added to Figure 1 B.

Statistics

- *Statistical analysis is absent throughout the paper*
- *Most of the time-series data show only a representative example, namely, N = 1. In relation to the aforementioned issue, data and distribution derived from several cells (e.g. SD) should be shown in a clear manner.*
- *Some form of statistical analysis should be performed on the data to give the reader*

© 2021. Published by The Company of Biologists under the terms of the Creative Commons Attribution License (https://creativecommons.org/licenses/by/4.0/).

a sense of robustness of the findings and its uncertainty. Either a non-parametric test on the median, confidence intervals or e.g. boxplots showing notches

- *(fig5) Unclear how the intensity at the specific organelles is measured? were the organelles segmented or hand-drawn ROI based? The quantified difference is very small, no statistics are performed, and it is unclear how it was measured. This is currently weak evidence for the main claim in this subsection*
- *Time-series now show single example traces (fig1C, fig2B,D, fig5B). It would be informative for the reader if the curves of all experiments were plotted, and statistical analysis would be performed on the data. It is unclear how representable the kinetics in these curves are*

__Action__: We will include error bars to our dotplots. We can show kinetics for dT-2xrGBD in more cells in supplement figure. We will indicate replicates.

Additional experiments:

About the spatial patterns of Rho activity (cytokinesis, tail retraction, ...), the reviewers agree that statistical analysis is much more difficult. But maybe showing 2-3 cells instead of only one, would make the data more convincing.

We can provide more data for unstimulated endothelial cells retracting form the data we have already acquired. Only one HeLa cell was imaged going through cell division. Since we have obtained a stable endothelial cell line with the dimericTomato-2xrGBD sensor in the meantime, we would propose to acquired data for these cell lines during mitosis instead of transfected HeLa cells.

mNeonGreen (mNG) was used as the fluorescent protein fused to rGBD instead of EGFP, which was used in the original paper. For a fair comparison with the previous report, analysis using the original probe, i.e., EGFP-rGBD, is desirable. Or, the author may simply tone done.

We will acquire data with the eGFP sensor in HeLa cells stimulated with histamine, which can be added to Figure 1B.

Original submission

First decision letter

MS ID#: JOCES/2021/258823

MS TITLE: Visualizing endogenous Rho activity with an improved localization-based, genetically encoded biosensor

AUTHORS: Elke Mahlandt, Janine J.G. Arts, Werner J. van der Meer, Franka H. van der Linden, Simon Tol, Jaap D van den Buul, Theodorus W.J. Gadella Jr., and Joachim Goedhart
ARTICLE TYPE: Research Article

I have now had time to go through your original MS submitted to review commons and the reviewers comments, which were favourable but raised some critical points that will require amendments to your manuscript. I see that you have already revised the text to some extent but are yet to add any new data to address the reviewers concerns. I think your response to the reviewers questions is appropriate and that the additional of the new data you suggest and better statistical analysis and representation will improve the paper. You might also consider using super plots to allow better appreciation of your data sets and statistical analysis (see https://www.molbiolcell.org/doi/10.1091/mbc.E20-09-0583).

We are aware that you may be experiencing disruption to the normal running of your lab that makes experimental revisions challenging. If it would be helpful, we encourage you to contact us to discuss your revision in greater detail. Please send us a point-by-point response indicating

© 2021. Published by The Company of Biologists under the terms of the Creative Commons Attribution License (https://creativecommons.org/licenses/by/4.0/).

where you are able to address concerns raised (either experimentally or by changes to the text) and where you will not be able to do so within the normal timeframe of a revision. We will then provide further guidance. Please also note that we are happy to extend revision timeframes as necessary.

Please ensure that you clearly highlight all changes made tot he text in the revised manuscript in a different colour in the PDF. Please avoid using 'Tracked changes' in Word files as these are lost in PDF conversion.

I should be grateful if you would also provide a point-by-point response detailing how you have dealt with the points raised by the reviewers in the 'Response to Reviewers' box. Please attend to all of the reviewers' comments. If you do not agree with any of their criticisms or suggestions please explain clearly why this is so.

First revision

Author response to reviewers' comments

We were very pleased with the generally positive comments on our manuscript. Several points were raised by multiple reviewers, which we addressed by including new experiments and by revising our data analysis.

A) Effect of the expression level on the performance of the probe
We have included an analysis of the sensor relocation amplitude versus the concentration (inferred from the fluorescence intensity). The resulting graph shows that the amplitude of the relocation is largely independent of the expression level. This data is included as supplementary figure S1C and S1D.

B) Selectivity of the probe for RhoA/B/C
We apologize for an unclarity in the submitted manuscript. We did not mean to claim that the sensor is able to differentiate between RhoA/B and C. We have changed the text to make this clear and we will call it Rho sensor.

C) Effect of the probe on cell morphology
We added an experiment in which we imaged a cytosolic fluorescent protein (mNeonGreen) in the HeLa cell histamine stimulation assay, to measure the effect of cell shape on the fluorescent intensity. This data is added to Figure 1B.

A point-by-point reply to all points follows below.

Reviewer #1 (Evidence, reproducibility and clarity (Required)):

Major points:

- The affinity analyses need more work. This is against A/B/C isoforms, and also the dimerization affinity between the fluorescent proteins could change the apparent on/off rates. This point is not quantified or discussed. Due to the chemical equilibrium analysis, the apparent equilibrium is not only affected by this on/off rates, but also the local availability (concentrations) of the reacting moieties. In the limit where the biosensor concentration is low within a cellular subcompartment or vice versa, how this is going to change the sensitivity of detection because this can push the reaction in either directions. Since equimolar distribution of the moieties are not guaranteed, this affects the detection characteristics of this biosensor. This point should be discussed and emphasized.
Regarding the A/B/C isoforms: We did not mean to claim, that the sensor is specific for RhoA, based on the literature, we are certain it will also bind Rho B and C. We observed binding to

© 2021. Published by The Company of Biologists under the terms of the Creative Commons Attribution License (https://creativecommons.org/licenses/by/4.0/).

active RhoB in an experiment not shown in the manuscript. To make this clearer, we changed the name of the Rho GTPase to Rho.
Regarding the dimerization affinity: Some initial data has been acquired for the weaker dimers Venus and iRFP. They seem to have a slightly beneficial effect on the relocation amplitude but less beneficial than the stronger dimer dTomato. We agree that the biosensor concentration affects the performance (which is an important point with respect to optimizing the right concentration, as will be discussed later). We think that the local availability is not limiting because of fast diffusion of the soluble biosensor. However, this may be an issue in highly polarized and elongated cell types such as neurons. This is added to the discussion: '*The biosensor concentration of relocation probes affects their performance. Although the diffusion of a soluble probe will not readily lead to differences in local availability in most cell types, this may be an issue in highly elongated or polarized cell types.*'

- Fig 1 A: Are the fluorescence changes of the biosensors due to stimulation with histamine completely reversible ? In other words, is it possible to see a total recovery of the signals with pyrilamine or in the presence of another antagonist ? If not, why?

We typically see almost complete inhibition of the effect of histamine with the antagonist that we use (pyrilamine). Although it is added at a saturating concentration, it cannot completely switch off the Rho GTPase activity. We do not know the reason, but this has also been observed with a DORA FRET sensor (e.g. Figure 4B in: https://doi.org/10.1124/mol.116.104505)

Does histamine stimulation induce a maximal activation of RhoA in HeLa cells? What happens in terms of fluorescence changes when the activity of RhoA is inhibited or in the presence of a Gαq-inhibitor, and in conditions in which RhoA activating GEF, RhoA GAP or RhoA GDI is overexpressed ? Generally, I think it is useful to have a calibration curve of the biosensors activity, maximal/minimal (ON/OFF) response. For exemple, it would help to answer the question concerning biosensors binding affinity for RhoA ("The function of rhotekin is not clear, it seems to lock RhoA in the GTP bound state (Ito et al., 2018; Reid et al., 1996). We can only speculate that rhotekin has a stronger binding affinity for active RhoA than anillin and PKN1 have." (p.15))

We have optimized our system to achieve high Rho activation and this has previously allowed us to do a quantitative comparison of the contrast of RhoA FRET sensors (see supplemental material of: https://doi.org/10.1038/srep14693). Whether this is a maximal response is unclear, but we do observe robust and consistently strong responses, which were not achieved by other strategies.

What is the effect of histamine stimulation on a membrane marker expression/location ?

We performed an additional experiment, measuring the fluorescent intensity for a cytosolic fluorescent protein in the HeLa cell histamine stimulation assay, since we measure the depletion in fluorescent intensity of the sensor in the cytosol. The data was added to Figure 1B.

What is the effect of histamine stimulation on dT2xrGBD biosensor response when this one is forced to be located in other subcellular compartments (mitochondria, nucleus) by fusing the construct to targeting sequences.

We have not tried this experiment and we are not sure what would be the point of that experiment? Rho in the cytoplasm would be an another compartment so one would not expect any response here. Furthermore, if the construct would be forced to localize, we would not observe relocalization.

Physiological control: Effect of the presence of the biosensor in cell morphology/behavior... Experimental data concerning this point are evoked in the discussion section. "We demonstrate that low expression of the biosensor, through the truncated CMV promotor, did not inhibit cell division and cell edge retraction. Plus, endothelial cells expressing the sensor still show the typical reaction of contracting followed by spreading, when stimulated with thrombin. Low expression results in a low fluorescent signal of the sensor." (p.16) I think this results would deserve a section in this manuscript.

© 2021. Published by The Company of Biologists under the terms of the Creative Commons Attribution License (https://creativecommons.org/licenses/by/4.0/).

This is the data shown in Figure 6 and 7 we will refer to it more clearly.
- Fig 2D : "The anillin sensor AHD+PH showed a 15% decrease in cytosolic intensity (Figure 2D), but it also relocalizes to striking punctuate structures upon histamine stimulation. These structures did not seem to represent local, high activity of RhoA, as the optimized rGBD sensor in the same cell showed no such locally clustered RhoA activation, but rather a homogenous activation at the membrane and a 60% drop in cytosolic intensity. Similar punctuate structures were observed in endothelial cells, when stimulated with the strong RhoA activator thrombin (Supplemental Movie 5)."
And p. 15 : "However, we noticed that the AHD+PH sensor, containing aGBD, C2 and PH domain, localizes in a punctate manner. These 'dots' were observed in both HeLa cells and endothelial cells and were only observed with the AHD+PH RhoA sensor. As aGBD does not localize in puncta, it seems that the localization is caused by domains other than of the RhoA binding domain, i.e. the C2- and/or PH-domain."
Punctate structures are also present in HeLa cells expressing the anillin sensor before histamine stimulation (see Supplemental Movie 4). Moreover, punctuate pattern activated by thrombin in endothelial cells looks different (more widespread) than the one activated by histamine in HeLA cells. In addition, these structures can also be found in human endothelial cells expressing dT2xrGBD (fig. 6B, Supplemental movie 10). What are those structures thrombin activated in endothelial cells that would be similar to the ones in Hela cells activated by histamine and that "did not seem to represent local, high activity of RhoA"? This is not further commented by the authors.
Very well spotted. What can be seen in Figure 7B and Movie 14, are different vesicles, that are always observed in endothelial cells expressing fluorescent proteins. We think they are autophagocytic endosomes/lysosomes in which some of the fluorescent proteins expressed in the cytosol eventually will accumulate over time. The more pH resistant red fluorescent proteins are visible in these structures (e.g. see Cell Structure and Function 33: 1-12 (2008) DOI: 10.1247/csf.07011). They do not localize at the membrane but in the cytosol. These structures are not induced by RhoA activation and are not present in the TIRF data which largely excludes the cytosol.

- Fig 3A: "The rGBD sensors solely colocalized in the nucleus with RhoA but not with Rac1 and Cdc42, indicating that rGBD specifically binds constitutively active RhoA." What about dT2xrGBD binding specificity for the three homologues RhoA, RhoB and RhoC?
This point is evoked in the discussion part (p.16) but there is no experimental data to support it "The specificity of the relocation sensor is determined by the binding specificity of the GBD. The rGBD binds the three homologues RhoA, B and C but not to Rac1 and Cdc42". So, why rGBD is presented as a RhoA biosensor?

We apologize for this misunderstanding. We have no reason to assume that the biosensor does not bind all three isoforms. We will refer to the RhoA/B/C isoforms as 'Rho' and we will call it a Rho sensor.

- Fig 3B: The data scatter for the dTomato-2xrGBD is very wide compared to the mScarlet-1xrGBD. What is causing this wide data scatter and such heterogeneous response? This is a problem if the sensor is really so heterogeneously responding to a strong mutant of RhoA, is this a dimerization-dependent problem?
It is not full clear why the spread in the data is more pronounced for dTomato-2xrGBD. We have added confidence intervals to show that the data for dTomato-2xrGBD is significantly different from no effect.

- These domain-based biosensors could cause dominant negative/inhibitory artefacts. Also the dimerizing fluorescent proteins could introduce oligomerization of the signaling complex which is not real in cells and clearly affect phenotype. These issues should be tested and addressed by a quantitative measure of cell behavior against increasing concentration/changing dimerization potentials of the biosensor in live cell assays.
We agree that these type of biosensors in a general sense can cause dominant negative/inhibitory artefacts and we explicitly mention this in the text: "Visualizing the endogenous Rho activity may interfere with the biological role of Rho, as the sensor binds endogenous Rho and may compete with natural effectors of Rho"

© 2021. Published by The Company of Biologists under the terms of the Creative Commons Attribution License (https://creativecommons.org/licenses/by/4.0/).

We were worried about this possible downside and have been very carefully looking at the effects of the biosensor. As highlighted in the manuscript, we noticed mitosis and natural contraction/spreading of endothelial cells. We were able to make stable cell lines. These are all signs that there are no strong negative effects. We also advice to use low expression of the sensor to limit negative effects: "To limit the perturbation, the sensor should be expressed at a low level to allow Rho signaling"

- Fig 4 C: "Given the successful improvement of the rGBD-based biosensor by increasing the number of binding domains, we explored whether the same strategy can be applied to the G protein binding domains from PKN1 and Anillin" and "The dimericTomato-2xrGBD sensor shows the best relocation efficiency, with a median change in cytosolic intensity of close to 50%"... So why the dT-2xaGBD construct has not been tried ?
We did not see the stepwise improvement as we saw for the rGBD sensor, so we do not expect an improvement in that construct. An additional reason is that the cloning for the 2xaGBD was initially not working out.

- p.9 : "None of the pGBD sensors showed a clear membrane localization upon stimulation with histamine (Figure 4A). The increase in cytosolic intensity observed in some cells, seems to be caused by changes in cell shape." Do changes in HeLa cell shape induced by histamine stimulation? How this can be explained? Do some cells expressing the rGBD sensors (single, tandem and triple and dimericTomato) undergo these changes of shape too, upon histamine stimulation? If yes, to what extent these changes in cell shape affect signals?
The activation of Rho GTPases by the histamine receptor often results in changes in cell shape in HeLa cells.
We performed an additional experiment with a cytosolic fluorescent protein in the HeLa cell histamine stimulation assay, to measure potential intensity changed solely caused by shape changes. The data was added to Figure 1B.

- p9: Overall, the paragraph about Fig 4 E,F is not clear. What amino acid sequences of G Protein Binding Domains of Anillin and PKN1 bring for the understanding of rGbD, aGBD and pGBD sensors?
Since there is no crystal structure for rGBD available, we thought it is interesting to compare the amino acid sequences to see how similar/ different these domains are.

- p. 12, Fig 6C, Fig. 6E: "The membrane marker showed a relatively small increase in intensity after stimulation and the curve did not show the same pattern as the RhoA biosensor intensity curve. Therefore, we conclude that the increase in RhoA biosensor intensity is caused by relocalization." It surprises me that decrease in cell areas induced a very small increase in fluorescence intensity of the membrane marker. It would be very helpful to see a figure with a quantification of the membrane marker intensity changes during this process. What about a cytoplasmic marker?
Figure 6B shows the intensity measurements of the membrane marker intensity. The small change can be caused by membrane changes, but also other factors that affect intensity (focus change). We added the membrane intensity measurements to Supplemental Figure S2 A as well. Since these measurements are made in TIRF, the intensity of the cytoplasmic marker would be very low. Therefore, we decided to use a membrane marker.

In addition, how does the movement artefact is corrected?
The ROIs were drawn by hand to measure the fluorescence intensity.

"Our data revealed that the RhoA biosensor displays RhoA activity at subcellular locations where RhoA activity is expected, and appears mostly independent of fluorescent intensity measured by a separate membrane marker." This part should be developed further. Are there examples of cells for which the biosensor activity is dependent on fluorescent intensity measured by a separate membrane marker?

The intensity of the membrane marker is only affected by changes in membrane area or morphology (and other technical reasons that lead to a change in intensity, e.g. focal drift, bleaching). This point is made in the paper by Dewitt that we cite (https://doi.org/10.1083/jcb.200806047). We are not aware of papers that show biosensor activity dependent on a separate membrane marker. One potential confounding issue is

© 2021. Published by The Company of Biologists under the terms of the Creative Commons Attribution License (https://creativecommons.org/licenses/by/4.0/).

quenching of the membrane marker by FRET, but this would lead to a decrease in intensity and we do not observe that.

- Discussion (p.16): "Comparing relocation sensors to FRET sensors, both have their own advantages and disadvantages." The dT2xrGBD sensor is here presented as a new relocation sensor for RhoA activity. However in general, there should be more development of the direct comparisons, pros and cons, with quantitative data or more details allowing to have a general overview of the advantages and disadvantages of this new relocation biosensor as compared to the existing ones.
We explain the pros and cons of FRET sensors and relocation sensors in the introduction and we show a quantitative comparison of this new relocation biosensor as compared to existing relocation biosensors (Figure 2). The advantage of the relocation sensor relative to a FRET sensor is highlighted in the discussion: "*Furthermore, the relocation sensor requires confocal microscopy or TIRF microcopy to spatially separate the bound from unbound probe, whereas FRET measurements are usually performed with widefield microscopes. However, the former mentioned techniques usually offer the higher resolution. Here we presented previously unachieved visualization of Rho activity at subcellular resolution. We observed local activation of Rho at the Golgi which was not possible with the DORA RhoA FRET sensor (Van Unen et al., 2015), indicating a higher sensitivity of the relocation sensor.*"

Minor points:
- Overall, scale bars should have to be included in HeLa cells microscopy images.

We now provide scale bars for all the figures.

- It was not clear until the Methods section that the widefield analysis appeared to be normalized against another fluorescent protein-based cytoplasmic signal to correct for variations in cell volume. I think this point should be mentioned in the main text more prominently and emphasized so that readers are not misled.

**The normalization of time traces has been done to account for differences in the initial intensity (e.g. due to differences in expression level), this is now better explained: "*The mean gray value or cell area respectively, were normalized by dividing each value by the value of the first frame, to account for differences in the initial intensity.*"
Of note, there is no extra cytoplasmic signal to correct for variations in cell volume.**

- p. 9 : "Anillin AH+PH sensor" instead of "Anillin AHD+PH sensor"

Corrected.

- Fig 2B and 2D : Explain what parameter is used for the normalization of each signals ?

We state in the methods: " *The mean gray value or cell area respectively, were normalized by dividing each value by the value of the first frame, to account for differences in the initial intensity.*"

- Fig. 1A, top panel: it would be good to know which images correspond to the addition of histamine and which ones correspond to the addition of pyrilamine

We added an indication of the stimulus to the images. Additionally, the time line with the grey bars indicating the stimulus of the graph matches the images. We changed the legend to clarify: "*The images match with the perturbation that is indicated for the plot in panel C.*"

- "TRIF microscopy" is written in legends of Fig. 6 and of Supplemental movie 11, and in Materiel and Methods section p. 23
- Fig. 3 legend: Correct "mScralet-I-1xrGBD"
- Fig 4F, legend: " Anillin and the bound RhoA are depicted in dark and light yellow, respectively. PKN1 and the bound RhoA are depicted in light and dark blue, respectively." Color codes in legend are opposites to the figure ones.

© 2021. Published by The Company of Biologists under the terms of the Creative Commons Attribution License (https://creativecommons.org/licenses/by/4.0/).

- p.11 : "To examine this, we used a rapamycin-induced hetero dimerization system to recruit the dbl homology (DH) domain, of the RhoA activating GEF p63, to the membrane of the Golgi apparatus." Corresponding references should be included.

Thanks for pointing these out, all have been addressed/corrected.

- Fig. 5A : Explain FRB, Fig 5C : no unit for a ratio

We changed the legend "*A) Still images of HeLa cells expressing FRB (part of rapamycin hetero-dimerization system) anchored to the membrane, Golgi and mitochondria (first column), FKBP-p63-DH (counterpart of rapamycin hetero-dimerization system, not shown), localization of the dimericTomato-2xrGBD sensor pre activation (second column) and post activation with 100 nM rapamycin (third column).*"

Reviewer #1 (Significance (Required)):

Mahlandt et al. optimized and compared several G protein binding domain (GBD)-based biosensors in order to improve the potential of existing RhoA-domain-based biosensors for visualizing and reporting RhoA subcellular activity in living cells and tissue. The authors demonstrate that fusing a dimerizing fluorescent protein to the rhotekin GBD (rGBD) is an efficient strategy to increase the brightness of the sensor. The use of Rhotekin-RBD as affinity domain for Rho-class of GTPase is very well established, both in the methods of affinity pulldowns and in biosensor designs for Rho-class of GTPases in the field. The authors show that the dimericTomato-2xrGBD biosensor can indicate endogenous RhoGTPase spatial activity in dividing HeLa cells and during cell retraction of human endothelial cells.

The dimericTomato-2xrGBD biosensor is thus introduced and described as a RhoA localization-based biosensor, however no experimental data demonstrate the binding specificity of the biosensor for RhoA. Moreover, authors discuss about a previous work showing that rGBD binds the three paralogs RhoA, RhoB and RhoC. This point and the apparent singular claim of this biosensor reporting RhoA activity as this manuscript alludes to are inappropriate and misleading.

We apologize for the misconception that this probe is specific for RhoA. We do not want to claim this sensor is specific for RhoA (and note that we have been involved in generating FRET biosensors for the different isoforms, RhoA/B/C ourselves: https://doi.org/10.1038/srep25502). We have addressed this in the introduction, and we have changed RhoA to Rho to better reflect that we are looking at all three isoforms.

This point especially in light of the field has moved on in the past 20 years to assign more specificity (not less) to which GTPase the biosensors are being specific, i.e., via FRET, etc., significantly tempers the enthusiasm of this reviewer. In addition to this main issue, the incomplete characterization of the relative affinities of the domain to the target GTPase isoforms and of the dimerization affinities of the fluorescent proteins (which could change the apparent reaction rate constants), and the impact of which on the reversibility, oligomerization states and detection sensitivity, and the biology, also appeared lacking. Additional stoichiometric considerations and apparent reaction equilibrium that are impacted by the relative concentrations of interacting moieties require careful and further analyses, study and discussion.
In general, I think that this work could be interesting to a more specialized field audience with further analyses of the affinities of the interacting moieties and better characterization of the behavior of this biosensor in living cells since it is likely causing oligomerization of the signaling units due to the forced dimerization of the detection unit.

Referees cross-commenting

This is a dimerizing probe. It gets pretty bulky. Is dimerization occurring prior to GTPase binding or after? Is the dimerized probe/GTPase complex somehow more stable than would otherwise be if they were monomeric? If so, how would that affect the lifetime of the detection and also the diffusivity of the probe("s", if already dimerized) and possibly the whole oligomer?

dTomato is shown to be a strong, obligate dimer. Therefore, we assume that the fluorescent probe is present as a dimer before (and after) binding to the GTPase.

© 2021. Published by The Company of Biologists under the terms of the Creative Commons Attribution License (https://creativecommons.org/licenses/by/4.0/).

With respect to size/bulkiness we'd like to note that the biosensor is only somewhat larger than a FRET sensor, i.e 2x47 kDa and 74 kDa, respectively.

It still feels to me that, yes new brighter fluorescent proteins were used, and dimerization and multimerization of the signaling complex increased the SNR of the system, but the whole premise just reverted the biosensor field back 20yrs, which has been my biggest single concern regarding this paper.

This evaluation is in our opinion largely based on the misconception that we claim RhoA specificity. We do not claim that this sensor is specific for RhoA (and we have revised the manuscript accordingly) and we are not aiming to replace FRET sensors (being quite fond of FRET sensors as is clear from our previous work).

We think that there is ample opportunities and applications for the improved relocation sensor (as is also evident from requests for the plasmids that encode the probe), for instance in experiment were FRET sensors are challenging to use, such as optogenetics experiments and multiplexing biosensors. We state in the discussion: "*Single color relocation sensors are ideal candidates for multiplexing experiments. Plus, the growing field of optogenetics is in need of single color biosensors to detect the effect of optogenetic perturbations. The conventional CFP-YFP FRET sensor is incompatible with most, blue light induced optogenetic tools.*"

Reviewer #2 (Evidence, reproducibility and clarity (Required)):

Summary:

Visualization of subcellular activity of GTPases is critical for the understanding of signal transduction of cell growth, differentiation, morphogenesis, etc. For this purpose, researchers often use relocation probes, which comprise a fluorescent protein(s) and a GTPase-binding domain(s), and move from cytosol to the location of active GTPases. The authors improved a previously reported RhoA probe with a strategy of increasing the avidity of RhoA-binding domain and optimizing the fluorescent protein. In the beginning, the authors declare "the relocation of the original, single rGBD monomeric fluorescent protein sensor is hardly detectable" in HeLa cells. To overcome this problem, they developed six constructs by changing the number of rGBD (rhotekin GBD) domains and fluorescent proteins. They found that the increase in the number of rGBD and a dimeric prone fluorescent protein, tdTomato, generate a better probe for RhoA activity. The specificity was examined by using active Rac1 and Cdc42 proteins. Different RhoA-bind domains derived from Rhotekin, PKN1, and Anillin were compared to show the superiority of rhotekin GBD. Finally, they show that subcellular RhoA activation detected by the probe is consistent with the knowledge on RhoA activation by using vascular endothelial cells. Overall this work has been well done in an organized way and disclose a novel RhoA probe that will be useful in future research of RhoA.

Major comments:

1. Reproducibility: The number of analyzed cells is described in the legend, but the number of independent experiments is not shown. This is critical to evaluate the reproducibility of the data. Preferably, the data should be presented to show data set derived from each trial clearly. It should also be described how cells were selected for the analysis? It is also preferable to apply automatic analysis. Ideally, the raw data with code sets for analysis should be presented.
The number of experiments is now indicated in the legends. Replicates for Figure 1B are now indicated in Figure S1B. ROIs were partly drawn by hand. We agree that segmentation based methods would increase reproducibility, but this data set is not suitable for automated analysis.

2. A serious defect of the relocation probe is the dependency on the expression level. The lower the number of the probe in a cell, the higher the fraction of recruited to active RhoA. However, lowering the probe concentration will be accompanied by dim fluorescence. The authors should describe how the optimal expression level was achieved.
We fully agree. However, we cannot measure a correlation between expression level and intensity (Figure S1 C,D). Using the low expression promoter improved the dynamic range but we have not gained control over the optimal expression level. It does vary from cell to cell. We

© 2021. Published by The Company of Biologists under the terms of the Creative Commons Attribution License (https://creativecommons.org/licenses/by/4.0/).

added this paragraph to the discussion: "*However, the optimal expression level is crucial for the dynamic range of the relocation sensor. Low concentrations of the sensor will show higher levels of relocalization, as a larger fraction of the sensor molecules binds the limited, active, endogenous Rho molecules. Nevertheless, if the concertation of sensor is too low, the fluorescent signal cannot be detected. To optimize the expression level, the CMVdel promoter, leading to a lower expression level, was applied (Watanabe and Mitchison 2002). Even though, this minimal promoter improved the performance of the relocations sensor, a variety of expression levels was observed. Cell sorting could be applied to select for cells with the optimal expression level.*"

3. Statistical analysis is absent throughout the paper.
We added 95% confidence intervals to the dot plots.

Minor comments:*

1. In Figure 1, mNeonGreen (mNG) was used as the fluorescent protein fused to rGBD instead of EGFP, which was used in the original paper. For a fair comparison with the previous report, analysis using the original probe, i.e., EGFP-rGBD, is desirable. Or, the author may simply tone done.
That is a good point. We performed the HeLa cell histamine stimulation assay for the eGFP-rGBD sensor and added the data to Figure 1B.

2. In the introduction, it says " The RhoA FRET sensors achieve subcellular resolution to a certain extent, but due to their design they do not localize as endogenous RhoA". Reference is required.
We changed the following in the introduction: *The RhoA FRET sensors achieve subcellular resolution to a certain extent, but due to their design they may not localize as endogenous RhoA (Michaelson et al., 2001).*

3. rGBD should be rhotekin GBD. It should be clearly stated in the beginning.
We wrote in the introduction: "*Secondly, the rhotekin G protein binding domain (rGBD)-based eGFP-rGBD Rho sensor, that was reported in 2005 (Benink & Bement, 2005).*" and in the results " *The eGFP-rGBD biosensor consists of an enhanced green fluorescent protein (eGFP) and a rhotekin G protein binding domain (rGBD).*"

4. The reason why the CMVdel promoter is used should be stated clearly.

Thanks for the suggestion. We added to the discussion:
"*However, the optimal expression level is crucial for the dynamic range of the relocation sensor. Low concentrations of the sensor will show higher levels of relocalization, as a larger fraction of the sensor molecules binds the limited, active, endogenous Rho molecules. Nevertheless, if the concertation of sensor is too low, the fluorescent signal cannot be detected. To optimize the expression level, the CMVdel promoter, leading to a lower expression level, was applied (Watanabe and Mitchison 2002). Even though, this minimal promoter improved the performance of the relocations sensor, a variety of expression levels was observed. Cell sorting could be applied to select for cells with the optimal expression level.*"

5. Page 23: TRIF should read as TIRF.
Corrected

6. Figures: Grey letters should be avoided.
We verified the figures for readability.

7. Fig. 3A: Apparently the probe binds to Rac1 G12V to some extent. The discrepancy of RhoA localization between mSca-1xrGBD and dt-2xrGBD must be discussed. This observation clearly suggests that GBD may change the localization of RhoA. It is interesting to note that Rac1 and RhoA may localize to the nucleolus.

We usually observe the dT-2xrGBD sensor to be excluded from the nucleus. However, in some cells it does localize not only in the cytosol but also in the nucleus. To us it appears that the overexpression of H2A-Rac1(G12V) results in more cells, in which the sensor is not excluded

© 2021. Published by The Company of Biologists under the terms of the Creative Commons Attribution License (https://creativecommons.org/licenses/by/4.0/).

from the nucleus, but we do not see a clearly increased accumulation in the nucleus in comparison to the cytosol. We have changed the text to make clear that the dTomato-2xrGBD binds better to RhoA than the 1xrGBD variant: “*Comparing the original single rGBD sensor (mScarlet-I-1xrGBD) with the dimericTomato-2xrGBD sensor, a higher nuclear to cytosolic intensity ratio for the multi-domain sensor was detected, supporting its higher affinity for RhoA.*”

Reviewer #2 (Significance (Required)):

1. This work discloses an improved RhoA probe, which will be welcome by the researchers in the field of small GTPases.

We are glad that the reviewer shares our enthusiasm

2. Novelty of increased GBD: The idea of increasing the GTPase-binding domain in the relocation probe was reported some time ago: Augsten et al., Live-cell imaging of endogenous Ras-GTP illustrates predominant Ras activation at the plasma membrane. EMBO Rep. 7, 46-51 (2006).
Agreed - we added the reference to the discussion: “*This strategy, to utilize multiple repeating domains has also been effective for a PH domain based lipid sensor and a cRAF derived Ras-binding domain Ras activity sensor (Augsten et al., 2006; Goulden et al., 2018)*”

3. Novelty of rhotekin GBD: The reason why GBD of PKN is chosen in intramolecular FRET biosensors such as DORA and Raichu is that the affinity of other GBD's is too high [Table 1, Yoshizaki et al., J. Cell Biol. 162, 223-232 (2003)]. Judging from this old data, GBD's of mDia and Rhophilin, may work better than that of Rhotekin. Moreover, it is known that PH domain may be required for proper conformation of GBD's. Thus, it is not surprising that removal of PH domain from the Anillin probe abolishes its translocation ability. Therefore, to the reviewer's eyes, the choice of GBD in Figure 4 is biased to those that will work less efficiently.

We see the point, but we have chosen these (PKN/anillin) for a practical reason, namely that we had cDNA encoding these probes in our lab. We thank the reviewer for the suggestion to look into other GBDs.

4. Authors' proposal of "systematic optimization" sounds exaggerated, considering the small number of constructs tested in Fig. 1 and Fig. 4. Similarly, it is not clear whether dimerize prone-fluorescent proteins are better choice by simply comparing tdTomato and mNeonGreen.

Fair enough, we think of it as a systematic comparison (Figure 1) and we have rephrased the sentence: “*Improving the rGBD probe by increasing the avidity was successful*”

5. Keywords of expertise: Fluorescent probes. Cell signaling.

Referess cross-commenting*

Because Review Commons does not specify the journal to be published, the request by the Reviewer #1 sounds too much. The probe reported in this work deserves publishing, although it may not be a ground-breaking probe.

We thank the reviewer for the encouraging words and support.

Reading the comments by the other reviewers, following concerns should be cleared.

1. Relationship between the probe's concentration and the response.

2. Specificity to RhoA, RhoB, and RhoC

3. The effect of the cell morphology as pointed by Reviewer #1.

Concern 1 is addressed by re-analysis of the data and the result is shown in Figure S1C,D. Concern 2 is addressed by changes in the text.

© 2021. Published by The Company of Biologists under the terms of the Creative Commons Attribution License (https://creativecommons.org/licenses/by/4.0/).

Concern 3 is addressed by control experiments that look into changes in cell morphology. This data (with a soluble fluorescent protein, mNG) is added to Figure 1B.

Reviewer #3 (Evidence, reproducibility and clarity (Required)):

Summary

In this paper, Mahlandt et al compared and improved relocation sensors to visualize the activity of endogenous Rho. As a result of screening for several Rho binding domains (GBDs) and the number of repeats, the authors found that dTomato-2xrGBD is optimal, and succeeded in visualizing the activity of Rho during cytokinesis and migrating cells.
Overall, this sensor would be a useful tool for many cell biologists. The data are represented clearly in the figures. I provide some concerns; that would be worth addressing in a revised version.

Major comments*

1. The authors should experimentally show the quantitative relationship between biosensor expression level and degree of relocation. In principle, this relocation type sensor binds to the endogenous GTP-bound Rho. Since the number of endogenous GTP-bound Rho is limited in cells, the degree of relocation is considered to be dependent on the expression level of the sensor. If the number of biosensors expressed is too small in a cell, the response will be saturated. If the number of biosensors is too large, the relocation will be weakened and the Rho signal will be suppressed. Furthermore, although a weak promoter is used, the heterogeneity of the expression level in each cell makes quantitative analysis difficult, especially in transient expression experiments. I would like to suggest the addition of quantitative experimental data.

We re-analyzed of our data to verify the effect of relative expression levels of the biosensor (based on intensity). The resulting data is added as supplemental figure S1C and S1D
We added to the introduction: "*A potential drawback is that background signal of the unbound biosensor in the cytosol, which may occlude the bound pool and reduce the dynamic range.*"
We added to the discussion: "*However, the optimal expression level is crucial for the dynamic range of the relocation sensor. Low concentrations of the sensor will show higher levels of relocalization, as a larger fraction of the sensor molecules binds the limited, active, endogenous Rho molecules. Nevertheless, if the concertation of sensor is too low, the fluorescent signal cannot be detected. To optimize the expression level, the CMVdel promoter, leading to a lower expression level, was applied (Watanabe and Mitchison 2002). Even though, this minimal promoter improved the performance of the relocations sensor, a variety of expression levels was observed. Cell sorting could be applied to select for cells with the optimal expression level.*"

2. Most of the time-series data show only a representative example, namely, N = 1. In relation to the aforementioned issue, data and distribution derived from several cells (e.g. SD) should be shown in a clear manner.
We focused not primarily on the kinetics, but more on maximal relocation, therefore we do not have time lapse movies for all the shown data points (e.g. a time lapse is shown in 1C and the data for a higher number of cells is shown in 1B). However, we can provide time series for multiple cells from our existing data sets. We added additional time traces in Figure S1A, S2A and we added measurements for multiple cells in Figure 2C,F.

Minor comments*

3. I hesitate to call the biosensor developed in this study "RhoA sensor". This is because, as the authors mention, it has been reported that the rGBD also binds to RhoB and RhoC. If the authors call it a RhoA sensor, they should investigate the specificity of binding to RhoB and RhoC in addition to RhoA. If not, I would like to suggest changing the name to "Rho sensor" instead of "RhoA sensor".
This is a fair point, also made by other reviewers. We will change the name to Rho sensor.

© 2021. Published by The Company of Biologists under the terms of the Creative Commons Attribution License (https://creativecommons.org/licenses/by/4.0/).

Reviewer #3 (Significance (Required)):

Rho is one of the low molecular weight G proteins, which regulate the reorganization of the actin cytoskeleton. As biosensors for visualizing the activity of Rho proteins, it has been reported intramolecular and intermolecular FRET biosensors and relocation sensors. The latter is less widely used than the former, because of insufficient sensitivity and specificity. Therefore, the improvement of Rho biosensors is really important and needed in the community of cell biology research field. The importance of this manuscript, I believe, is that the authors compared the existing relocation type Rho sensors. This is informative.

Rho is one of the low molecular weight G proteins that regulate the rearrangement of the actin cytoskeleton. Intramolecular and intermolecular FRET biosensors and relocation sensors have been reported as biosensors for visualizing the activity of Rho proteins. The latter is not as widely used as the former due to its inadequate sensitivity and specificity. Therefore, improving the Rho biosensor is very important and is needed by the community in the field of cell biology research. I believe the importance of this manuscript is that the author compared existing relocation-type Rho sensors. This is beneficial and informative.

My expertise: Cell biology, live-cell imaging, development of genetically encoded fluorescent probes

We thank the reviewer for the positive evaluation of our work.

Referees cross-commenting

I generally agree with Reviewer 2's opinion. The opinions of our three reviewers can be summarized in three points: expression level, specificity, and statistical analysis and representation. I think these should be asked to the authors as major critics that should be addressed before publication.

We agree and we have addressed the three main points (see also response to reviewer 2).

Reviewer #4 (Evidence, reproducibility and clarity (Required)):

SUMMARY:

Mahlandt and colleagues use advanced microscopy techniques to test new configurations of several Rho relocation sensors, which report on the activity of members of the endogenous RhoA GTPase family of proteins. A novel variant containing the dimericTomato fluorescent protein and a double rGBD domain shows a substantial increase in dynamic range in comparison with 2 originally published sensors and other new variants they tested.
They use a cellular assay to show that this novel variant is specific for the activity of Rho family of Rho GTPases and not the Cdc42/Rac families. Finally, the authors show that this new variant can be used to measure a specific localised increase of Rho activity at the Golgi, and during cell division and cellular morphology changes that are known to activate the RhoA family of Rho GTPases. The biosensor can be useful for the community. However, I think the paper is not well written (I was very confused by several statements). The manuscript should be thoroughly proofread, there are quite some unclear or duplicate passages (for examples, see "text comments" below). Currently this hampers the interpretation of the manuscript for the reader. The authors are very dogmatic - they make claims about the literature that I do not agree with at all. Some of these unbalanced views will confuse the non-expert readers.

MAJOR COMMENTS:

-The reported dTomato-rGBD sensor is unable to distinguish between the different members of the RhoA familiy of Rho GTPases (measures combined activity of RhoA, RhoB and RhoC), which is unclear for the reader in the current text phrasing in the introduction. The authors seemingly suggest throughout the manuscript to work with a specific RhoA biosensor, which is not the case. This strong statement is completely misleading. The authors need to refer to the biosensor being

© 2021. Published by The Company of Biologists under the terms of the Creative Commons Attribution License (https://creativecommons.org/licenses/by/4.0/).

specific for Rho (RhoA,B,C) GTPases versus Rac1/Cdc42 biosensors, and discuss what this means for the field. Some discussions about this are made in a JCB paper by Graessl et al, that the authors also cite.
We agree that the probe measures the combined activity of all three isoforms and apologize for the confusion. We have changed the name to Rho sensor and updated the manuscript.

-If the authors really want to sell that the biosensor is only specific for RhoA, then they need to make a series of experiments with RhoB and RhoC dominant positive/negative constructs, to tackle that specific point.
No, we did not intend to claim the sensor is specific for RhoA in comparison to Rho B and C.

-Did the authors consider to use the artificial GBD from Keller, 2019 to make a specific relocation sensor for RhoA? Perhaps the authors can comment on the feasibility of this approach?
We think that this might be the only way to make a specific RhoA relocation sensor. Recently, we have received the DNA and performed the histamine stimulation experiment in HeLa cells as in Figure 1B. However, the anti-RhoA nanobody did not show an efficient relocalization in our assay. The data has been added to Figure S4.

-A strong (dogmatic) statement is that Rho GTPases FRET sensors report solely on the activity of GEFs. This is not the case, these sensors report on the flux of GAP and GEF activity for Rho GTPase in cells. This is also true for relocation sensors, and has been documented in work from the Bement/Pertz/Nalbant/Dehmelt labs.

We thank the referee for this correction and we have changed the text to: "*By design, these FRET sensors report on the balance between activating guanine exchange factors (GEFs) and inactivating GTPase-activating proteins, instead of visualizing endogenous RhoA-GTP*"

-From the data in Figure 1, it seems to follow that the efficiency of PM relocation is mainly determined by the number of rGBD modules on the sensors. Could the authors speculate on how this works in practice; is the multi-rGBD sensor increasingly kinetically trapped by a single RhoA molecule, or is the sensor mostly bound to multiple RhoA molecules at the PM?
This is an interesting question to which we do not have an answer. We added some text to the discussion: "*It is currently not clear how each of the GBDs of the dimericTomato-2xrGBD sensor contribute to Rho binding and the probe may bind between 1 and 4 Rho molecules. If the probe is capable of binding multiple Rho proteins, the binding efficiency will depend on the local density of Rho in the membrane.* "

-Some form of statistical analysis should be performed on the data to give the reader a sense of robustness of the findings and its uncertainty. Either a non-parametric test on the median, confidence intervals or e.g. boxplots showing notches.

We have included 95% confidence intervals in our dot plots.

-Time-series now show single example traces (fig1C, fig2B,D, fig5B). It would be informative for the reader if the curves of all experiments were plotted, and statistical analysis would be performed on the data. It is unclear how representable the kinetics in these curves are.

We focused not primarily on the kinetics, but more on maximal relocation, therefore we do not have time lapse movies for all the shown data points (e.g. a time lapse is shown in 1C and the data for a higher number of cells is shown in 1B). However, we can provide time series for multiple cells from our existing data sets. We added additional time traces in Figure S1A, S2A and we added measurements for multiple cells in Figure 2C,F

-About the spatial patterns of Rho activity (cytokinesis, tail retraction, ...), the reviewers agree that statistical analysis is much more difficult. But maybe showing 2-3 cells instead of only one, would make the data more convincing.

We provide more examples, and these are now added as Figure S3.

© 2021. Published by The Company of Biologists under the terms of the Creative Commons Attribution License (https://creativecommons.org/licenses/by/4.0/).

MINOR COMMENTS:

-(fig4a) dTomato-2xpGBD, why is this not good? how is it possible that it binds good to nucleus, but no translocation is observed? const activity? expression levels?

We were surprised and somewhat disappointed by this as well and we do not have an explanation, besides that the binding affinity required for dynamic relocation seems to be higher than the one for binding the overexpressed active Rho GTPase.

-(fig4f) The aGBD/pGBD binding sites for RhoA show great overlap but bind to completely different sites at RhoA, is this correct? (color scheme used for the structures is not easily interpretable)

It is correct they both have two binding sites but apparently, they found crystals for one or the other. Maesaki et al. 1999 is describing the two binding site. We have changed the colors.

-(fig5) Unclear how the intensity at the specific organelles is measured? were the organelles segmented or hand-drawn ROI based? The quantified difference is very small, no statistics are performed, and it is unclear how it was measured. This is currently weak evidence for the main claim in this subsection.
ROIs are drawn by hand. We added 95% confidence intervals in our dot plots.

-(fig5) The kinetics of the response to histamine (fig1C) seems to be much faster as the rapamycin mediated increase in fig5B for the PM condition. Any explanation for this? Why does it not reach a plateau like in the histamine experiments?

It is probably the recruitment of the p63-DH that takes more time than the activation of the H1R and the downstream signaling. Both FRB and FKBP were tagged with mTurquoise2, which makes it difficult to compare recruitment kinetics of FKBP-p63 to the recruitment of the dT-2xrGBD sensor. Plus, in Fig 1C the intensity was measured in the cytosol and Fig 5B the intensity was measured at the plasma membrane, which makes the kinetics not directly comparable.

-(fig6F) Data from 6D is repeated here, 6F could potentially show aggregate time-series instead of individual cells. Would also improve interpretation if the membrane marker curve is plotted in every subfigure. Potentially membrane marker intensity could be used to normalise the (TIRF) measurements?

We added the membrane intensity to Sup Fig. 2A.

-can the authors provide scale bars on the micrographs, as is usually done in any manuscript ? It would also be useful to put time labels when images corresponding to timeseries are shown.

We now provide the scale bars for every figure.

-ratio values are dimensionless by definition, so no need to write "arbitrary units"

We changed it.

TEXT COMMENTS:

-(abstract): "Due to the improved avidity of the new biosensors for RhoA activity, cellular processes regulated by RhoA can be better understood." -> unclear what the authors mean with 'avidity' in this context? (here, and throughout rest the manuscript)
Avidity refers to "*the accumulated strength of multiple affinities*", we added this explanation to the text in the introduction. Another paper working with multiple biding domains to improve a relocation sensors also calls it avidity: A high-avidity biosensor reveals plasma membrane PI(3,4)P2 is predominantly a class I PI3K signaling product (Goulden at al. 2018 JCB).

-(introduction) "Although these three Rho GTPases may have different functions, we generally

© 2021. Published by The Company of Biologists under the terms of the Creative Commons Attribution License (https://creativecommons.org/licenses/by/4.0/).

refer to RhoA in this manuscript." -> unclear what message the authors try to convey with this sentence.
We changed to: "We will use 'Rho' throughout the manuscript, which refers to all three isoforms"

-(introduction) "Active RhoA mainly localizes at the plasma membrane, due to its prenylated C-terminus" -> where else would it be localised? Where is inactive RhoA localised?

We included: *"Active Rho mainly localizes at the plasma membrane, due to its prenylated C-terminus (Garcia-Mata et al., 2011).However, a fraction of RhoA has been found at the Golgi apparatus. Inactive RhoA, in comparison, can be extracted from the plasma membrane by Rho-specific guanine nucleotide dissociation inhibitors (RHOGDIs) (Garcia-Mata et al., 2011)".*

-(introduction) "Unimolecular Rho GTPase FRET-based biosensors consist of the Rho GTPase itself, a GBD and a FRET pair." -> a short description/explanation of what a "FRET pair" is would benefit the non-specialised audience.
We included: *"Unimolecular Rho GTPase FRET-based biosensors consist of the Rho GTPase itself, a GBD and a FRET pair, which is commonly a cyan (CFP) and a yellow fluorescent protein (YFP)."*

-(Results p9) "For the original Anillin AH+PH sensor...around 15%" -> did the authors do the experiment with G14V on this original sensor variant?
Yes, it is supposed to say AHD+PH here as well, which has been corrected. We performed the experiment with mScarlet-AHD-PH.

-(Results p9) The "mScarlet-I-AHD+PH" seems to perform quite good on the fig4D assay, but is not present in 4C analysis?
eGFP-AHD+PH was used as the original sensors for the 4C assay. Due to the color of the RhoA G14V (mTq2) we switched to the mScarlet version to exclude bleed through. We assume that the sensor performs similar with different monomeric fluorescent proteins.

-(Results p9) "mScarlet-I-AHD+PH" is the same as "AHD+PH (aGBD+C2+PH)"? descriptions unclear. Would generally advise to thoroughly check the manuscript for consistency of condition descriptions / abbreviations in both text and legends.
Changed to: AHD+PH (consisting of aGBD+C2+PH).
We mention earlier: "*Moreover, a published relocation sensor AHD+PH based on Anillin contains, next to a G protein binding domain, also a C2 and a PH domain and localizes in punctuate structures which do not represent Rho activity (Figure 2C,Supplemental Movie 4 and 5) (Munjal et al., 2015; Piekny & Glotzer, 2000). Here, we used only the G protein binding domain of Anillin (aGBD) as a basis for another sensor."*

-(Results p12) "Visualizing endogenous RhoA activity" as subsection title could potentially confuse readers, since all measured Rho activity in the manuscript is endogenous.

That could indeed be confusing. What we intending to highlight is that we did not overexpress any signaling molecules or receptors in these experiments. In the first paragraph of the results, we added: "*Upon histamine addition, it binds the activated, endogenous Rho and thereby relocalizes to the plasma membrane, where active Rho is localized, and it relocalizes to the cytosol when pyrilamine, a histamine antagonist, is added."*

minor text:

-(fig3b legend) "mScralet-I-1xrGBD"
Corrected

-(fig6H legend) "TRIF", and "cbBOEC" is same as "BOEC"?

It is a detail, but these are indeed different and we have updated the materials and methods to better reflect this: "*cord blood Blood Outgrowth Endothelial cells (cbBOEC)*" and "*Blood Outgrowth Endothelial cells from healthy adult donor blood (BOEC)*"

© 2021. Published by The Company of Biologists under the terms of the Creative Commons Attribution License (https://creativecommons.org/licenses/by/4.0/).

Reviewer #4 (Significance (Required)):

The novel "Rho" family GTPase relocation sensor that the authors present might be a significant improvement over the currently existing ones (for refs, see manuscript). This might provide a substantial technical advance in the field and increases the utilisation and the reproducibility of this tool in the field. This sensor will be of significant interest for the Rho GTPase signalling field, and more broader the cytoskeleton biology community. My expertise in Rho GTPase biology, biosensor development and advanced microscopy granted me the opportunity to judge the complete manuscript

The reviewer thinks that the new sensor will be of significant interest and we agree.

Second decision letter

MS ID#: JOCES/2021/258823

MS TITLE: Visualizing endogenous Rho activity with an improved localization-based, genetically encoded biosensor

AUTHORS: Elke Mahlandt, Janine J.G. Arts, Werner J. van der Meer, Franka H. van der Linden, Simon Tol, Jaap D van den Buul, Theodorus W.J. Gadella Jr., and Joachim Goedhart
ARTICLE TYPE: Research Article

Thank you for sending your manuscript to Journal of Cell Science through Review Commons.

I am happy to tell you that your manuscript has been accepted for publication in Journal of Cell Science, pending standard ethics checks.

© 2021. Published by The Company of Biologists under the terms of the Creative Commons Attribution License (https://creativecommons.org/licenses/by/4.0/).
